# Step towards High Power Factor in Acidic-Doped Poly(3-hexylthiophene) Systems

**DOI:** 10.1021/acsomega.5c09899

**Published:** 2025-12-15

**Authors:** Szymon Gogoc, Pawel Gnida, Anna Adamczyk, Aleksandra Wypych-Puszkarz, Krzysztof Wojciechowski, Przemyslaw Data

**Affiliations:** † Department of Inorganic Chemistry, Faculty of Materials Science and Ceramics, 49811 AGH University of Krakow, Mickiewicz30, Krakow 30-059, Poland; ‡ Centre of Polymer and Carbon Materials, 111480 Polish Academy of Sciences, Marii Sklodowskiej-Curie 34, Zabrze 41-819, Poland; § Department of Silicate Chemistry and Macromolecular Compounds, Faculty of Materials Science and Ceramics, 513364 AGH University of Kraków, Mickiewicza 30, Krakow 30-059, Poland; ∥ Department of Molecular Physics, Faculty of Chemistry, 3057 Lodz University of Technology, Zeromskiego 116, Lodz 90-924, Poland; ⊥ Department of Physics, Durham University, South Road, Durham Dh1 3le, U. K.

## Abstract

In this study, we present dodecylbenzenesulfonic acid (DBSA) as a cost-effective alternative to F4TCNQ for doping poly­(3-hexylthiophene) (P3HT). DBSA not only acts as a p-type dopant but also serves as a surfactant, influencing the material’s morphology and electronic properties. We investigated the impact of dopant concentration (ranging from 0.0001% to 20% v/v) on electrical conductivity, Seebeck coefficient, and power factor in spin-coated thin films. Seebeck coefficient values ranged from 59 μV·K^–1^ (at 12% v/v DBSA) to 352.9 μV·K^–1^ (at 0.1% v/v DBSA), while the optimal P3HT:DBSA composition (11% v/v DBSA) exhibited a conductivity of 5.58 S·cm^–1^, a Seebeck coefficient of 69.7 μV·K^–1^, and a power factor of 2.706 μW·m^–1^·K^–2^. Atomic force microscopy (AFM) revealed that higher DBSA concentrations reduced surface roughness, influencing the conductivity. The obtained thermoelectric parameters are comparable to those of sulfuric acid and F4TCNQ-doped P3HT, highlighting DBSA as a promising and less toxic alternative for organic thermoelectrics.

## Graphical abstract

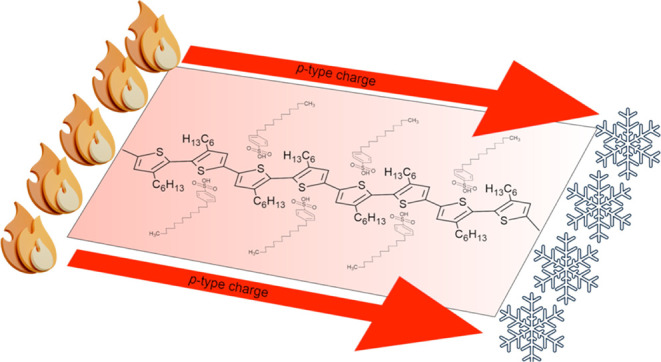

## Introduction

Thermoelectric materials have gained increasing attention due to their ability to directly convert heat into electricity, presenting a sustainable and efficient solution for power generation and waste heat recovery. The thermoelectric effect was first observed by Thomas Seebeck in 1820, who found that a temperature difference across a junction of dissimilar metals induces an electric potential.^1–3^ This effect, now known as the Seebeck effect, is the foundation of thermoelectric power generation. Conversely, in 1834, Jean Peltier discovered the Peltier effect, which describes the cooling or heating that occurs when an electric current is passed through a junction of two materials.^4,5^ These phenomena have since been utilized in various applications, including radioisotope thermoelectric generators (RTGs), which have powered space probes such as *Pioneer 10* and *11*,^6^ and compact refrigeration systems developed as early as the 1960s.^7^

While inorganic thermoelectric materials, such as bismuth telluride (Bi_2_Te_3_) and silicon–germanium alloys, have dominated the field due to their high efficiency, they are often brittle and expensive and require rare or toxic elements. Organic thermoelectric materials (OTEs) have emerged as a promising alternative due to their lightweight nature, flexibility, solution processability, and tunable electronic properties.^8–10^ These properties make the OTEs particularly attractive for wearable electronics, flexible sensors, and portable energy-harvesting devices. However, organic materials typically exhibit lower electrical conductivity than their inorganic counterparts, necessitating the use of dopants to enhance their charge transport properties. Among organic thermoelectric materials, poly­(3-hexylthiophene) (P3HT) is widely studied due to its thermal stability, high solubility in organic solvents, and tunable electronic properties. The first report on P3HT in 1988 explored its thermochromic behavior in the solid state,^8^ while its potential in organic photovoltaics was demonstrated following the introduction of tetrafluorotetracyanoquinodimethane (F4TCNQ) as a p-type dopant in 2007.^9^ This doping strategy significantly improved P3HT’s electrical conductivity and broadened its range of applications. The first use of F4TCNQ-doped P3HT in thermoelectric devices was reported in 2014, demonstrating its potential as an organic thermoelectric material.^10^ Further studies also explored device-level optimization and module design based on F4TCNQ-doped P3HT, highlighting the challenges associated with doping uniformity and contact resistance.^11^ However, F4TCNQ poses significant challenges due to its high toxicity and high cost, limiting its widespread adoption in commercial applications.^12^ To overcome the drawbacks of F4TCNQ, alternative p-type dopants have been explored. One promising candidate is 4-dodecylbenzenesulfonic acid (DBSA), which has been reported to significantly enhance the electrical conductivity of P3HT while maintaining low toxicity and lower cost compared to F4TCNQ.^13,14^ DBSA introduces proton transfer doping, in which the acid donates protons to the conjugated polymer, altering its charge carrier density and improving conductivity (Figure 1). This mechanism is similar to that observed in poly­(3,4-ethylenedioxythiophene) polystyrenesulfonate (PEDOT:PSS), where poly­(styrene sulfonic acid) acts as a p-type dopant.^15–23^ Additionally, DBSA functions as a surfactant, affecting the film’s morphology, surface roughness, and phase separation, which in turn influence its thermoelectric properties. Another potential alternative dopant is sulfuric acid (H_2_SO_4_), which has been used for chemical doping of conjugated polymers, including P3HT. Sulfuric acid doping introduces sulfate anions, which can stabilize polarons and bipolarones, leading to enhanced electrical conductivity and increased charge carrier mobility. Studies have shown that H_2_SO_4_-doped P3HT exhibits a significantly higher Seebeck coefficient and power factor compared to pristine P3HT.^24,25^ However, H_2_SO_4_ doping presents challenges, such as potential polymer degradation, instability under ambient conditions, and difficulty in controlling doping levels. Despite these issues, sulfuric acid doping remains a valuable method for enhancing thermoelectric properties, particularly when combined with processing techniques that mitigate degradation effects. While chemical doping improves electrical conductivity, it also affects the morphology and structural integrity of the polymer films. High concentrations of DBSA or H_2_SO_4_ can lead to phase separation, micelle formation, and film shrinkage, which negatively impact charge transport properties. These structural changes have been observed using atomic force microscopy (AFM), where films with excessive dopant loading exhibit increased roughness and cracks, reducing their electrical performance. Furthermore, solvent selection plays a crucial role in preventing such defects. For instance, using high-boiling-point solvents, such as chlorobenzene instead of chloroform, can reduce polymer shrinkage and improve film uniformity.^24,25^ Previous studies have reported the effects of alkylbenzenesulfonic acids, including DBSA, as surfactant dopants in P3HT.^26–29^ These works mainly established that such acids can induce conductivity increases, but the reported improvements were modest and lacked comprehensive modeling of the charge transport mechanisms. In contrast, the present study extends this field by systematically mapping the doping–morphology–performance relationships across thin films and pellets, supported by Kang–Snyder transport modeling and weighted mobility analysis. To further understand the impact of DBSA and H_2_SO_4_ doping on thermoelectric performance, our study systematically investigates the relationships between dopant concentration, film morphology, and key thermoelectric parameters, including electrical conductivity, Seebeck coefficient, and power factor. By analyzing these dependencies, we aim to determine the optimal doping conditions that maximize the thermoelectric efficiency of the P3HT-based materials.

**Figure 1:**
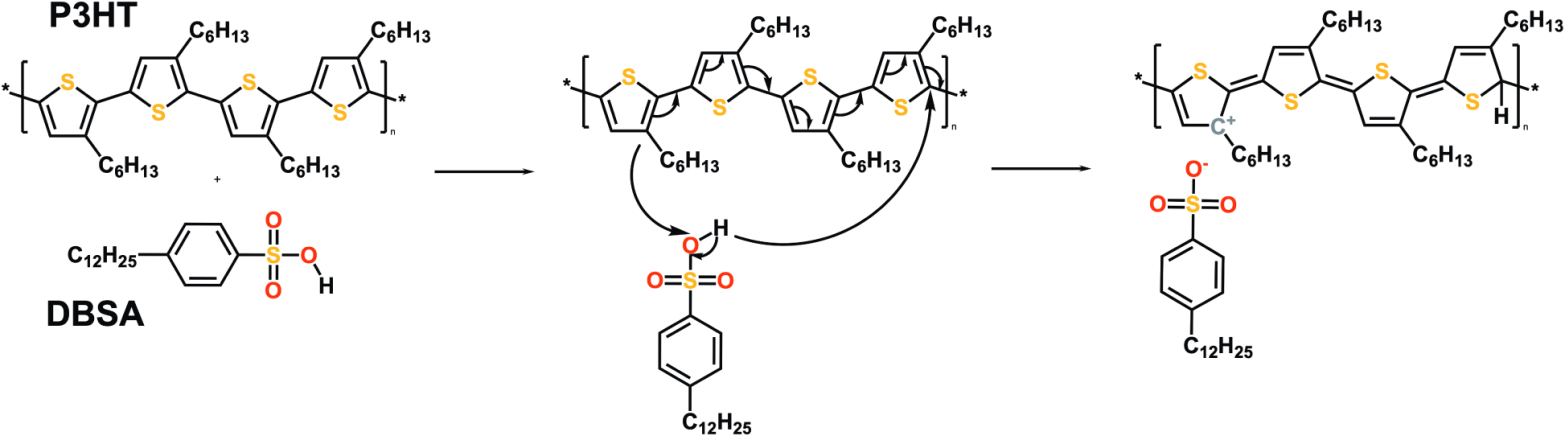
Scheme of proton transfer occurring between P3HT and DBSA.

Besides its solution and chemical routes, poly­(3-hexylthiophene) (P3HT) offers one of the most versatile platforms for electrochemical synthesis among polythiophene derivatives. The relatively low oxidation potential of 3-hexylthiophene enables smooth electropolymerization under mild conditions, giving films or nanostructures directly on the conductive substrates. This path avoids stoichiometric oxidants and allows the process to be powered by electricity, aligning with green chemistry principles. Furthermore, the electropolymerization medium can be modified with different counterions (e.g., sulfonates, perchlorates, phosphates, organic acids), producing stable doped materials in a single step. Compared with other thiophene derivatives, P3HT combines good solubility of the resulting polymer with high structural order, which is beneficial for thermoelectric and electronic applications. The same methodology also permits copolymerization with bi- or terthiophene monomers, opening the possibility to tune the regioregularity, conjugation length, and doping level in a straightforward way. Recent studies report P3HT nanowires, smooth films, and even soluble fractions synthesized electrochemically in batch or flow systems, illustrating the feasibility of this sustainable route.^30–32^

The novelty of this work lies in the integration of thin-film and pellet thermoelectric studies under controlled doping conditions, the application of advanced transport models (Kang–Snyder framework) to DBSA-doped P3HT, the demonstration that solvent engineering mitigates film cracking at high DBSA content, and the identification of DBSA as a safer and scalable alternative to toxic F4TCNQ and aggressive H_2_SO_4_ doping. Together, these contributions distinguish this study from prior reports on DBSA-doped P3HT, such as that by Alveroglu, which primarily focused on the spectroscopic and electrical aspects of acid doping rather than a comprehensive thermoelectric characterization.^13^

## Results and Discussion

### Experimental Section

The experimental setup was based on the Netzsch SBA 458 Nemesis system for thermoelectric measurements interfaced with a computer running a LabView environment for raw data acquisition. Atomic Force Microscopy (AFM) measurements were performed using a NanoSurf CoreAFM in contact mode and a Bruker AFM Multimode 8 instrument in peak force tapping mode. Scanning Electron Microscopy (SEM) images were acquired using a NOVA NANO SEM 200 instrument to analyze the surface morphology and microstructure. Polynomial Texture Mapping (PTM) photographs were taken with a Fujifilm camera equipped with automated custom lighting equipment to capture surface textures. Broadband Dielectric Spectroscopy (BDS) analysis was conducted using a Novocontrol system, which includes a high-resolution ALPHA-ANB dielectric analyzer with an active head sample cell (ZGS), an RF impedance analyzer (Agilent E4991) with an RF sample cell (BDS 2100, gold-plated electrodes, low-loss RF extension line BDS 2201), and a temperature control system (QUATRO Cryosystem) operating in the range of −160 °C to +400 °C. The data acquisition and analysis were carried out using the WinDETA-ALL, WinTEMP, WinPLOT, and WinFIT software packages.

Poly­(3-hexylthiophene) (P3HT) (M1011, Mw = 61,500) was purchased from Ossila and used as received. The dopant dodecylbenzenesulfonic acid (DBSA) (mixture of isomers, ≥95%) was obtained from Sigma-Aldrich. Both materials were stored under inert conditions to prevent the oxidation and degradation.

For sample preparation, P3HT was dissolved in chloroform at a concentration of 5 mg/mL. Different DBSA concentrations were added to the polymer solution, ranging from 0.0001% v/v to 20% v/v (DBSA:3HT molar ratio from 0.000108 to 21.6) to investigate the influence of dopant content on thermoelectric and morphological properties. Additionally, reference solutions were prepared by adding concentrated sulfuric acid (H_2_SO_4_) to the P3HT solution in chloroform with concentrations of 0.1%, 0.2%, 0.3%, 0.4%, 0.5%, 0.6%, 0.7%, and 0.8% v/v. Upon increasing the dopant concentration, a noticeable color change in the solutions was observed, indicating chemical interactions between the dopant and the polymer backbone. Glass substrates (15 × 15 mm) were cleaned using a multistep ultrasonic treatment to ensure optimal adhesion and film uniformity. The cleaning process involved:

1. Sonication in acetone for 15 min to remove organic contaminants.
2. Sonication in isopropyl alcohol (IPA) for 15 min to eliminate residual surface impurities.
3. Drying under a nitrogen stream to prevent dust contamination.

After cleaning, the glass substrates were placed on carbon electrodes and thin films were prepared via spin coating at 2000 rpm to ensure uniform film formation. The deposited films were then annealed at 120 °C for 1 min to improve film stability and remove residual solvents (Figure 2).

**Figure 2:**
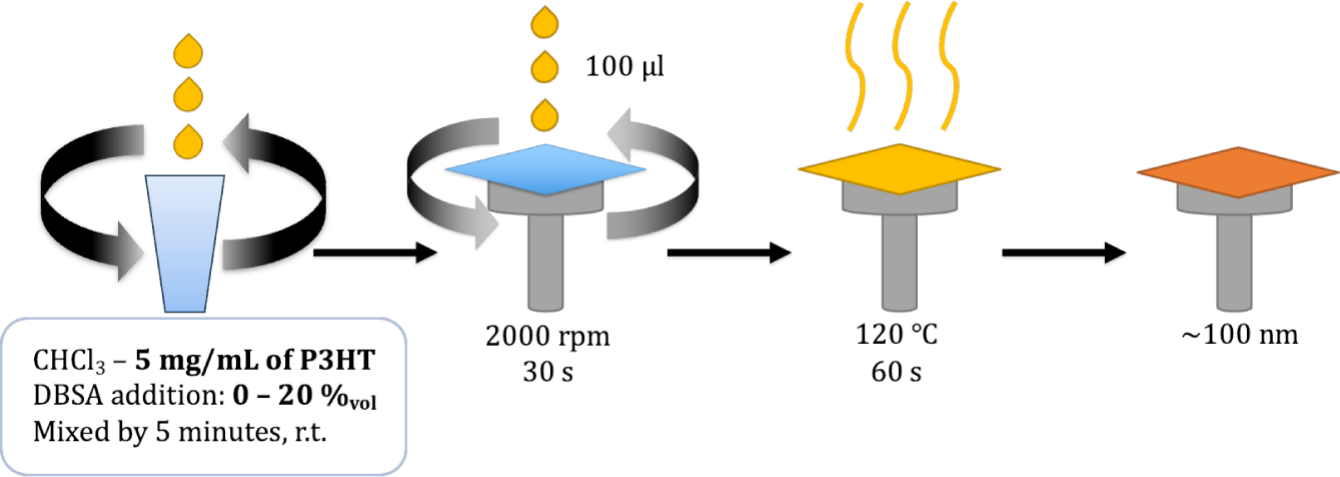
Preparation of the samples.

### Results

Atomic Force Microscopy (AFM) analysis revealed a strong correlation between the dopant concentration and film thickness. For a DBSA:3HT molar ratio of 3.24, the film thickness reached 111 nm, which is 2.5 times greater than that of the film deposited with a dopant:mer ratio of 0.1080.

The increase in film thickness with a rising DBSA content (Figure S70) can be attributed to the higher effective viscosity of the P3HT:DBSA solution during spin-coating. Since the spin parameters and solids content were kept constant, the observed trend directly reflects the relative viscosity of the coating solution. In addition to the intrinsic viscosity of DBSA, intermolecular interactions between P3HT chains and DBSA molecules likely contribute to the increased effective viscosity and thicker deposited films. However, the observed viscosity increase cannot be explained solely by the intrinsic viscosity of DBSA. Upon the addition of the acid, proton transfer and dipole formation between DBSA and the thiophene backbone occur, leading to enhanced interchain interactions and partial aggregation of P3HT chains. These molecular associations increase the effective hydrodynamic volume of the polymer in solution, thereby contributing to the overall viscosity rise. Consequently, the viscosity change reflects both the physical properties of DBSA and the chemical interactions within the P3HT:DBSA system. Such enhanced interactions within the polymer matrix are also reflected in the film morphology, as revealed by AFM imaging, which shows a clear dependence of surface structure on dopant concentration. This effect is particularly pronounced at higher DBSA concentrations, where the increased viscosity results in thicker deposited films. Additionally, AFM imaging indicated that surface roughness was higher for less-doped samples, as illustrated in Figures S1–S5. This is likely due to morphological changes occurring as DBSA interacts with the P3HT chains. At higher DBSA concentrations, the layer topography begins to resemble a wave-like structure, which suggests that DBSA not only functions as a dopant but also modifies the polymer film’s morphology, thereby affecting electrical conductivity.

Despite the positive effects of DBSA doping on film thickness and conductivity, some challenges were observed at higher DBSA concentrations (≥15% v/v DBSA). One major issue was a loss of adhesion to the glass substrate, making contact-based electrical measurements difficult. Additionally, the formation of cracks in the polymer layer was observed, which is crucial for charge transport. Scanning Electron Microscopy (SEM) images (Figure 3a) provided further evidence of undissolved DBSA aggregates at ≥15% v/v DBSA doping. This phase segregation occurs due to the nonpolar nature of the solvent, which promotes DBSA self-association and the formation of DBSA-rich aggregates rather than uniform molecular-level doping. As a result, a significant portion of DBSA remains inactive, leading to a decline in the thermoelectric performance.

**Figure 3:**
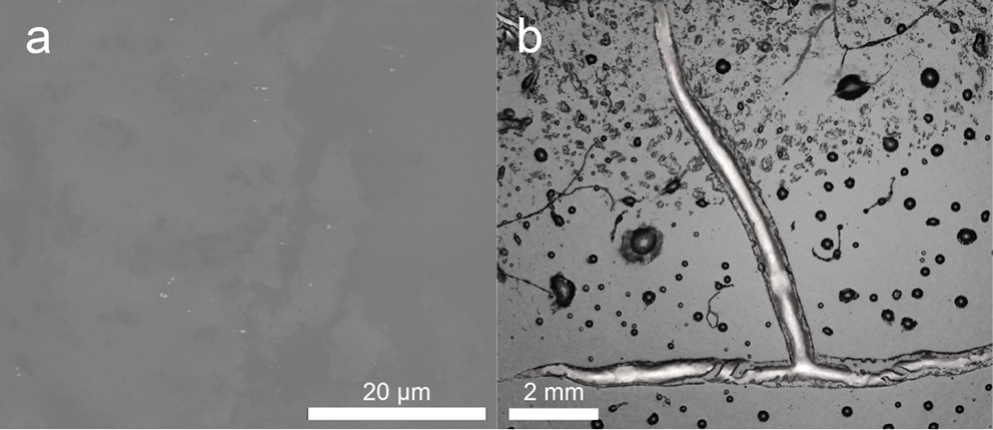
SEM image of the P3HT layer with 12.96 of DBSA:3HT, with undissolved DBSA in the P3HT matrix (a) and Polynomial Texture Mapping photo of the same layer showing a crack on the layer (b).

To investigate this issue further, Polynomial Texture Mapping (PTM) imaging was used to examine defects in DBSA-doped films. PTM is a powerful technique that captures 50 images under varying lighting conditions, allowing for detailed defect visualization.^29^ As shown in Figure 3b, PTM imaging clearly reveals a large crack spanning the entire film width, which compromises charge transport and significantly reduces the electrical conductivity. The formation of such defects is attributed to shrinkage during film drying, which alters the polymer chain structure. This shrinkage is further examined in AFM images in Figure 4, where the presence of phase separation is observed at a 6% v/v DBSA concentration. The dark regions in Figure 4b indicate phase separation, an effect that becomes even more pronounced as DBSA concentration increases.^33^

**Figure 4:**
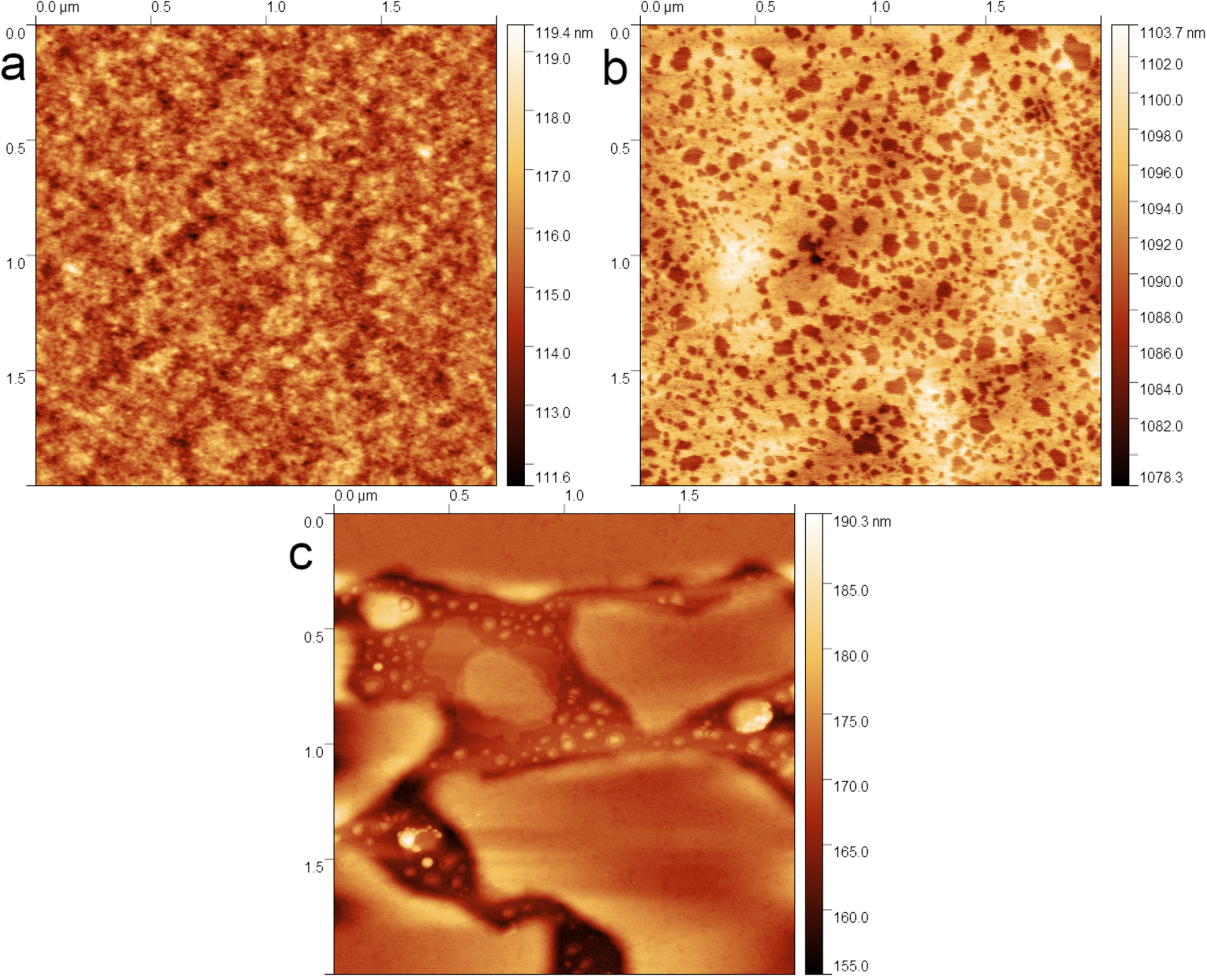
AFM images of P3HT:DBSA (a—0, b—6, c—12%_v_) film, with undissolved DBSA in the P3HT matrix.

The addition of DBSA significantly alters the morphology of the P3HT films through a combination of chemical interactions and structural reorganization. Infrared spectroscopy confirmed the presence of hydroxyl and sulfonic groups associated with DBSA within the polymer matrix, indicating protonation and partial incorporation of the acid into the P3HT backbone. This protonation induces local polarization along the conjugated chain and facilitates charge delocalization through the formation of polarons and bipolarons, as supported by Raman spectra showing a red shift and subsequent blue-shift of the C=C stretching band with increasing DBSA concentration.

Atomic force microscopy (AFM) and scanning electron microscopy (SEM) analyses further revealed that DBSA affects the surface continuity and internal microstructure of the films. At lower doping levels, DBSA acts as a surfactant, reducing surface roughness and promoting more homogeneous film formation. However, as the DBSA content increases beyond approximately 12% v/v, phase separation and micelle-like aggregation occur due to the limited solubility of DBSA in chloroform. These effects lead to local shrinkage during solvent evaporation, resulting in the formation of cracks and a two-phase morphology composed of polymer-rich and DBSA-rich domains.

Such morphological transitions correlate strongly with the electrical transport properties. Moderate DBSA addition improves ordering and charge carrier delocalization, enhancing conductivity, whereas excessive doping introduces structural defects that interrupt percolation pathways and decrease the charge transport efficiency. The overall mechanism is consistent with previous reports on acid-doped conjugated polymers, where the balance between protonation-induced ordering and acid-induced phase segregation determines the final film morphology and performance.

One possible strategy to prevent these defects is to use higher-boiling-point solvents that evaporate more slowly, thereby reducing film shrinkage and allowing for a more uniform dopant distribution. Another potential solution is to modify the fabrication process by employing alternative techniques, such as drop-casting or vapor-phase doping, which could minimize structural defects.

The Seebeck coefficient (α) was determined by direct voltage measurement under an applied temperature gradient using eq 1:

1 $$\alpha =-dV/dT\;\left[V\cdot K^{-1}\right]$$

Since the relationship between temperature and voltage is linear, the derivative was calculated numerically.^34,35^ Measurements were conducted at room temperature under controlled conditions. Figure 5a,b, and c illustrates the relationship between hydrogen ion concentration and key thermoelectric parameters, including (a) electrical conductivity, (b) Seebeck coefficient, and (c) power factor. The highest Seebeck coefficient (352.9 μV·K^–1^) was observed at 0.1% v/v DBSA, but this sample exhibited high resistivity (∼1 GΩ) (Table 1), making it impractical for thermoelectric applications. A similar trend was reported by Zhang and Park, where highly doped P3HT films showed an increase in conductivity at the cost of Seebeck coefficient reduction.^36^ To optimize the thermoelectric efficiency, a compromise between charge carrier density and carrier mobility must be achieved. Figure 5a illustrates the relationship between the hydrogen ion concentration and key electrical properties. Our results closely match those of Kroon et al., who demonstrated that acid-doped polythiophenes exhibit a charge transport behavior dictated by localized states.^37^

**Figure 5:**
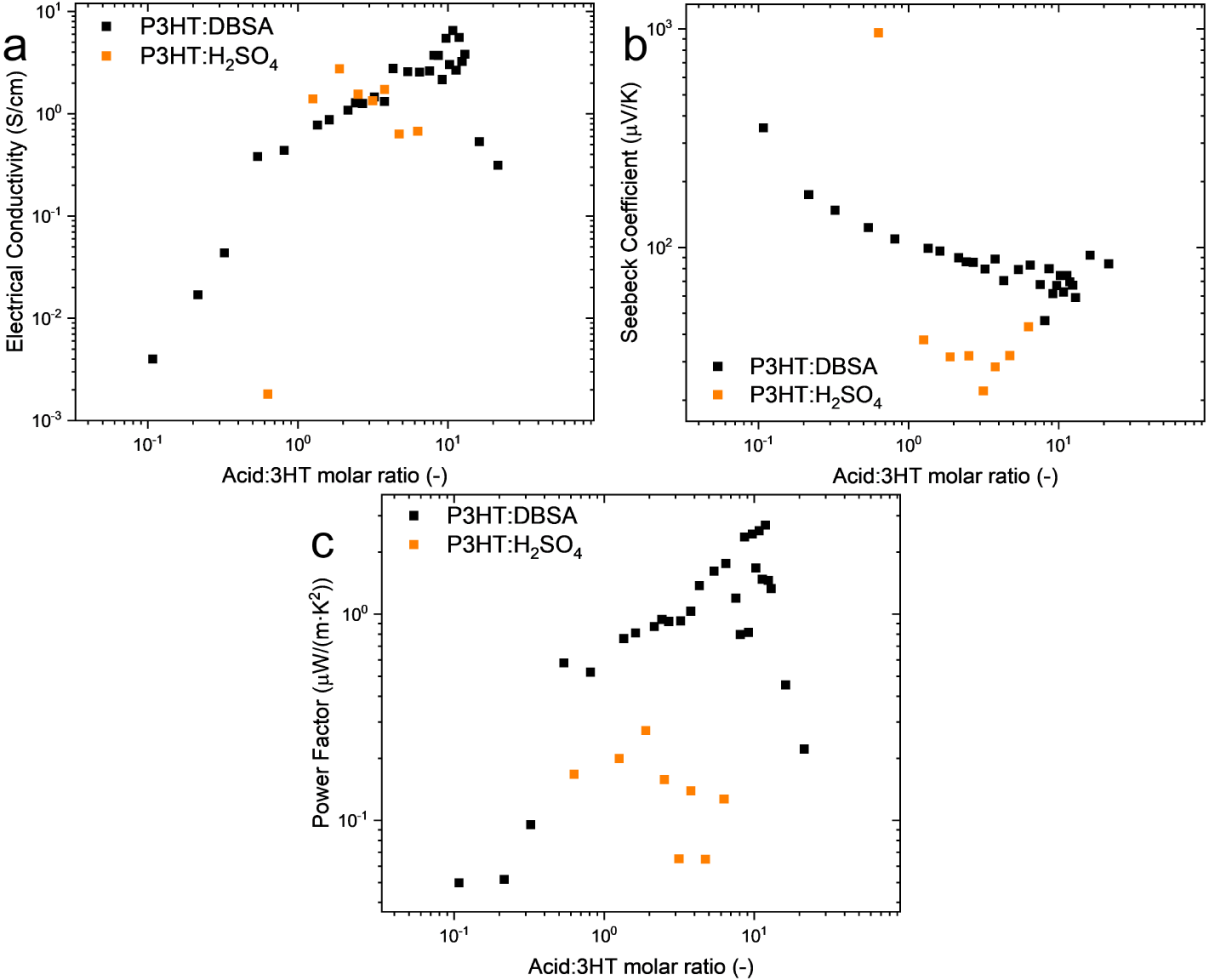
Dependence between hydrogen ions and (a) conductivity, (b) Seebeck coefficient, and (c) Power Factor.

**Table 1:** Results of Thermoelectric Measurements of P3HT:DBSA-Based Devices and Reference Devices with Sulfuric Acid Addition

Sample (3HT:dopant molar ratio)	Conductivity ***σ*** [S·cm^–1^]	Seebeck coefficient [μV·K^–1^]	Power factor ***σ* *α*** ^2^ [μW·m^–1^·K^–2^]
P3HT + DBSA (1:21.6)	0.31	82.1	0.22
P3HT + DBSA (1:16.2)	0.53	92.08	0.45
P3HT + DBSA (1:12.96)	3.82	59.0	1.33
P3HT + DBSA (1:11.88)	5.58	69.7	2.706
P3HT + DBSA (1:10.80)	6.51	62.4	2.54
P3HT + DBSA (1:8.64)	3.72	79.9	2.37
P3HT + DBSA (1:6.48)	2.55	83.08	1.76
P3HT + DBSA (1:4.32)	2.77	70.4	1.38
P3HT + DBSA (1:3.24)	1.46	79.7	0.93
P3HT + DBSA (1:2.70)	1.26	85.5	0.92
P3HT + DBSA (1:2.16)	1.08	89.7	0.87
P3HT + DBSA (1:1.62)	0.88	96.2	0.81
P3HT + DBSA (1:0.8099)	0.44	109.3	0.52
P3HT + DBSA (1:0.324)	0.044	147.9	0.095
P3HT + DBSA (1:0.216)	0.017	174.5	0.052
P3HT + DBSA (1:0.1080)	0.0040	352.9	0.050
P3HT + DBSA (1:0.000)^31^	9.67 × 10^–6^	1550	0.0023
P3HT + H_2_SO_4_ (1:0.6308)	0.026	263	0.18
P3HT + H_2_SO_4_ (1:1.89)	0.093	443	1.82
P3HT + H_2_SO_4_ (1:3.15)	0.38	93	0.33
P3HT + H_2_SO_4_ (1:3.78)	0.045	259	0.30
P3HT + H_2_SO_4_ (1:6.308)	0.066	206	0.28

Using eq 2, the thermopower (α) was modeled as a function of conductivity (σ):^38–44^

2 $$\alpha =k_{B}e^{-1}\sigma^{-0.25}\sigma_{\alpha }^{0.25}\;\left[\mu V\cdot K^{-1}\right]$$

Physically, the fourth-root dependence captures hopping/percolation transport in disordered conjugated polymers: as doping increases, the percolation network densifies, and the typical hopping energy (hence the entropy per carrier entering the Mott relation for *α*) scales with (σ/σ_α_)^1/4^. Over our σ range, this is equivalent to a Jonker-type trend and compactly represents the reduction of *α* with increasing carrier concentration.

In the equation *k*_B_ *e* ^–1^ is considered as the natural unit of thermopower, which is 86.17 μV·K^–1^, while σ_α_ is the free parameter set on 1 S·cm^–1^.^10,35^ Power factor can be calculated using eq 2 and the Power Factor formula (σα^2^), resulting with eq 3:

3 $$PF=k_{B}^{2}e^{-2}\sigma^{0.5}\sigma_{\alpha }^{0.5}\;\left[\mu W\cdot m^{-1}\cdot K^{-2}\right]$$

Conductivity measurements were performed for all samples to assess the impact of the DBSA doping levels. A clear increase in conductivity was observed as the dopant concentration increased. However, for samples with DBSA concentrations below 0.1% v/v, it was not possible to measure the Seebeck coefficient due to the significant increase in resistivity, which exceeded the detection limit of the measurement system. Despite the deterioration of certain thermoelectric parameters at high doping levels, the overall correlation between the conductivity and thermoelectric performance remained consistent.

The electrical conductivity of all P3HT samples was measured using the four-probe method. The resistance of each sample was determined immediately before the thermoelectric measurements to ensure accuracy. Conductivity (σ) was calculated using the following equation:

4 $$\sigma =I/\left(2\pi V\right)\left[\left(1/s_{1}-1/s_{2}\right)-\left(1/s_{3}-1/s_{4}\right)\right]\;\left[S\cdot m^{-1}\right]$$

Where *I* is the current flowing between outer probes, *V* is the potential difference between the inner probes, and *s*_1_, *s*_2_, *s*_3_ and *s*_4_ are the distances between probes.^45^

All sample parameters are shown in Table S2, when in main article we will show results of the most important samples. Table 1 presents the thermoelectric performance of P3HT:DBSA thin films, confirming that optimal performance occurs at 11.88% v/v DBSA, yielding Seebeck coefficient: 69.7 μV·K^– 1^, Electrical conductivity: 5.58 S·cm^– 1^, and Power factor: 2.706 μW·m^–1^·K^–2^. These values are comparable to sulfuric acid-doped P3HT and exceed the performance of traditional polyaniline-based thermoelectrics, as reported by Yusupov and Vomiero.^46^ Unlike previous DBSA studies that focused exclusively on thin films, our inclusion of pelletized systems provides insight into bulk thermoelectric behavior with ZT values approaching 10^–3^ at elevated temperatures. This extension highlights DBSA’s potential beyond thin-film devices. The thermal conductivity (κ) values presented in the Supporting Information (Figures S58–S60) were obtained for pelletized P3HT:DBSA samples using a Netzsch LFA 457 MicroFlash in a through-plane configuration. In contrast, electrical conductivity and Seebeck coefficient were measured in-plane using the Netzsch SBA 458 Nemesis system. This distinction highlights that the κ data reflect bulk transport properties, whereas the main text focuses on the in-plane behavior of thin films.

Figure 6a and b illustrates the relationship between conductivity and Seebeck coefficient, as well as the correlation between power factor and conductivity. Based on the measured conductivity, the power factor of the devices was calculated, as it is a crucial parameter for assessing their thermoelectric performance. The correlation between the power factor and conductivity is illustrated in Figure 6b. Both relationships align well with the trends described in eq 2 and eq 3.

**Figure 6:**
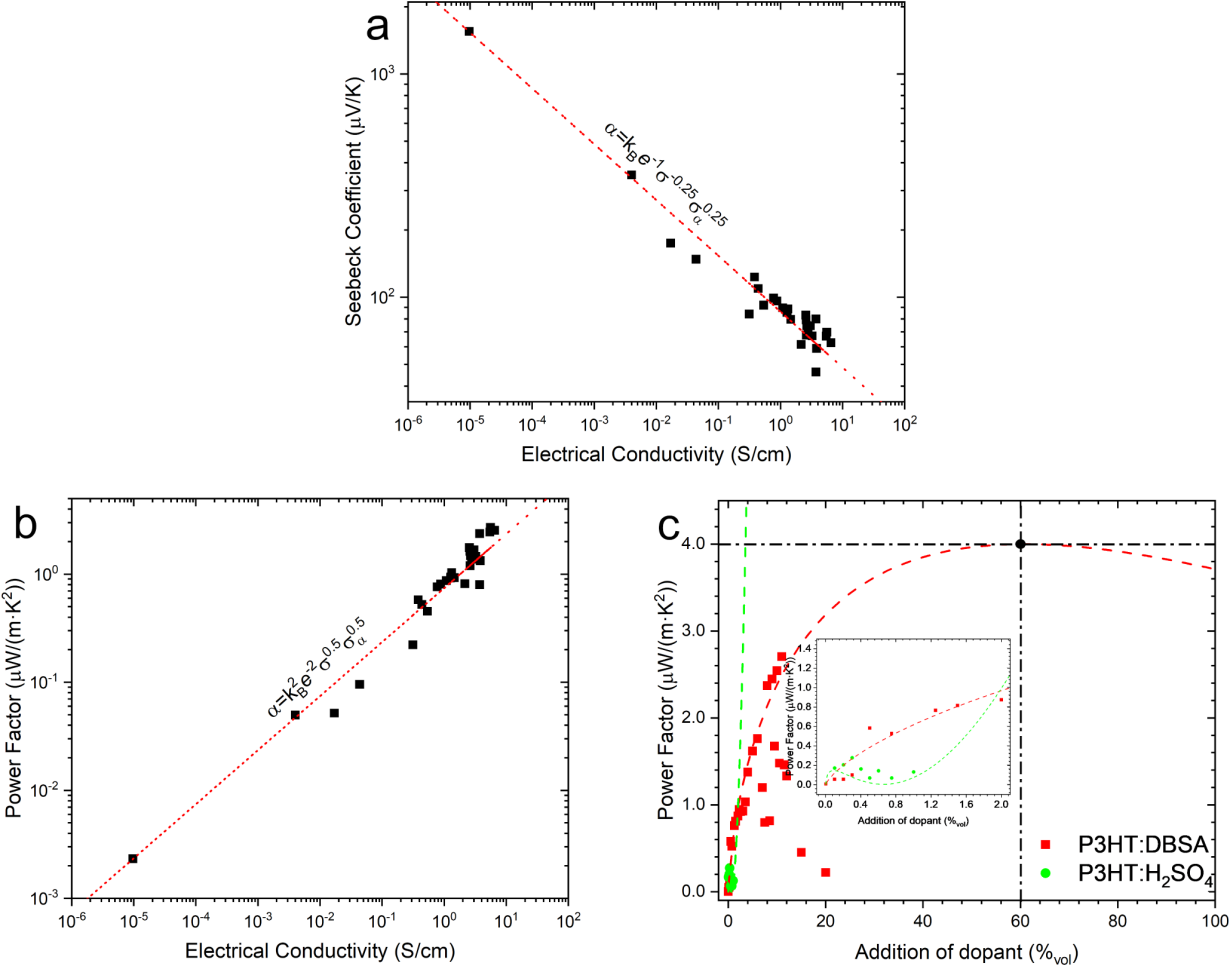
Correlation between the electrical conductivity of the P3HT:DBSA system and (a) Seebeck Coefficient, (b) Power Factor with math relationship for polythiophenes, and (c) Fitting of the model with the calculated maximum point for P3HT:dopant systems.

To determine the optimal DBSA doping level for maximizing the thermoelectric power factor (PF), we employed the following empirical equation, commonly used for conducting polymers such as polyacetylenes, polypyrroles, and polyanilines:^47^

5 $$\alpha =-k_{B}/e\cdot 1/\beta \cdot \ln\left(\sigma /\sigma_{max}\right)\;\left[\mu V\cdot K^{-1}\right]$$

Where α is the Seebeck coefficient, *k*_B_ is Boltzmann’s constant, *e* is the elementary charge, β represents an empirical transport prefactor related to the ratio of conductivities (or mobilities) of charge carriers of opposite signs, and *σ/σ*_max_ represents the normalized electrical conductivity.

The fitted β value obtained from eq 5 was 4.83, which is consistent with the empirical correlation proposed by Mateeva et al. and indicates partially ambipolar charge transport in DBSA-doped P3HT. This moderate β value suggests that hole conduction remains dominant, while a minor contribution from electron-like carriers may reduce the overall Seebeck coefficient. The Mateeva model was applied here as an empirical benchmark to describe the σ–α correlation, while the subsequent Kang–Snyder approach provides a physically grounded framework for extracting transport coefficients and carrier energetics in doped P3HT.

This equation was originally developed to model the charge transport behavior in conjugated polymers, where the power factor does not exhibit a monotonic trend with doping but instead follows a peak-like dependence due to the competing effects of charge carrier concentration and mobility. Figure 6c illustrates the fitted curve derived from eq 5, which accurately captures the relationship between the DBSA concentration and the resulting thermoelectric performance. Our calculations indicate that the maximum power factor (4.00 μW·m^–1^·K^–2^) is achieved at 60% v/v DBSA addition (which corresponds to a 1:64.8 DBSA:3HT molar ratio), closely matching literature reports for acid-doped P3HT systems. In our initial modeling, we assumed a linear relationship between conductivity and dopant concentration, based on the experimental data. However, as reported by Kroon et al., this assumption is an oversimplification because charge carrier mobility and doping efficiency do not scale linearly with dopant addition. Instead, at higher dopant concentrations, the formation of insulating phase-separated regions (micelles) reduces charge carrier delocalization, leading to a deviation from linear behavior.

To account for these nonlinear effects, we compared our results to alternative charge transport models from the literature. A comparison of our thermoelectric generator (TEG) performance with previously reported polythiophene-based devices is shown in Figure 7. The observed trends are consistent with Glaudell et al.’s empirical model for conducting polymers, confirming that DBSA-doped P3HT follows a behavior typical of acid-doped organic thermoelectrics.

**Figure 7:**
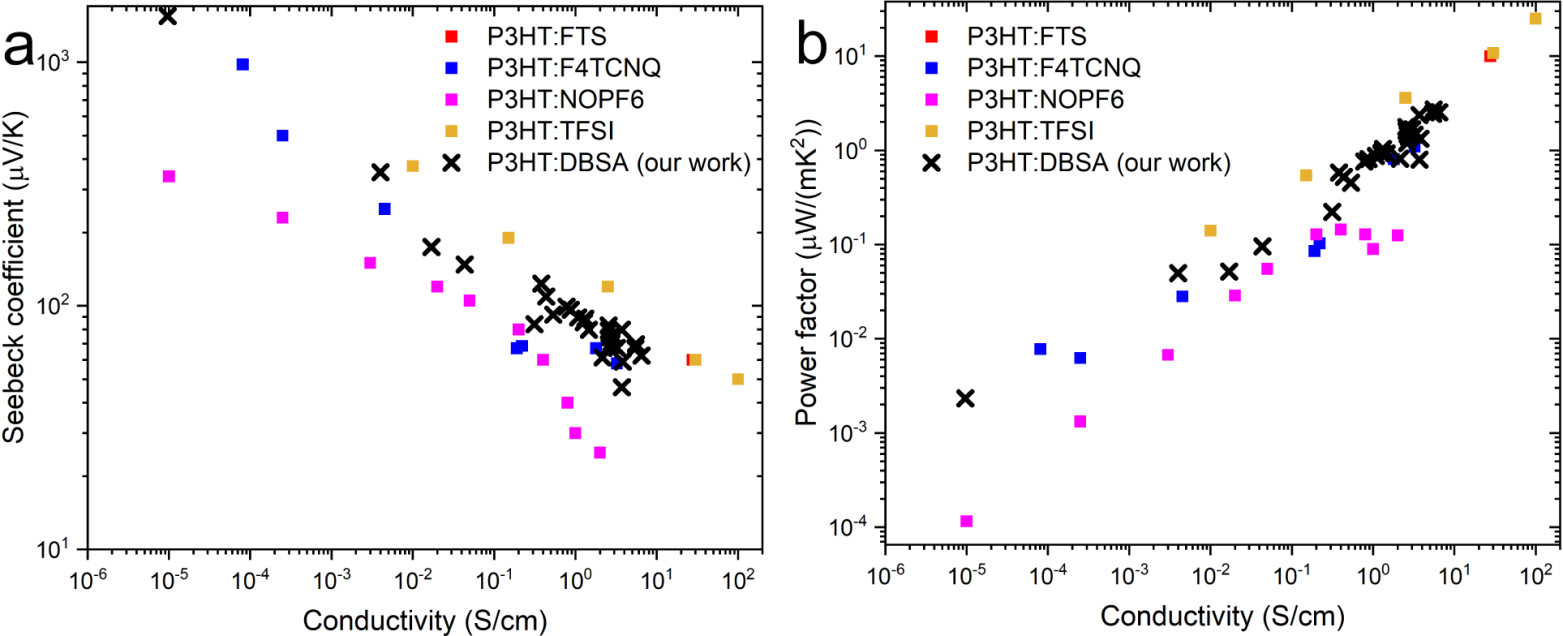
Comparison of (a) Seebeck Coefficients and (b) Power Factors of our samples and results from the literature.^31–37^

Although our power factor values appear comparable to those reported in earlier acid-doped P3HT studies,^13,26^ the combination of detailed morphology analysis and transport modeling uniquely allows us to predict the maximum achievable PF and clarify the role of structural defects in limiting performance. To compare the validity of different charge transport models, we selected the Kang et al. model for conducting polymers. This model provides a more accurate charge transport simulation, as it accounts for carrier mobility, density of states, and polaron hopping effects, unlike the equation of Mateeva et al., which assumes a linear relationship between dopant concentration and conductivity. Since the relationship between conductivity and dopant concentration in organic semiconductors is highly nonlinear, the Mateeva model does not sufficiently capture the complex transport phenomena occurring in DBSA-doped P3HT. The Kang et al. model is closely related to the Glaudell et al. model, as both emphasize the strong coupling between conductivity (σ) and the Seebeck coefficient (α) in conducting polymers. In our study, P3HT:DBSA thin films were analyzed to extract their transport coefficient using the following equation:^48^

6 $$\alpha \left(1/\sigma \right)=86,17\cdot 4\cdot F_{3}\left(\eta \right)\cdot \sigma_{E_{0}}\left(T\right)\cdot 1/\sigma -86,17\eta$$

Where *F*_3_(*η*) is the Fermi integral function for a given chemical potential η, $\sigma_{E_{0}}(T)$ is the conductivity at the transport energy level *E*_0_, and the prefactor *k*_B_ */e =* 86.17 μV·K^– 1^ represents the natural thermopower unit, corresponding to the thermal energy per elementary charge. Expressing *α* in units of *k*_B_ */e* allows direct comparison of thermopower values with the characteristic energy scale of charge transport in disordered semiconductors.

By applying eq 6, we determined that the transport coefficient for our P3HT:DBSA series is 1.51 × 10^–3^ S·cm^–1^, a value consistent with the theoretical expectations for thermally activated transport in acid-doped conjugated polymers. However, this value was obtained by considering all experimental results, including those from defective layers. When recalculating the transport coefficient, excluding defective layers (e.g., cracked films with lower conductivity), we observed a significant increase in the transport coefficient value, aligning better with the predicted values for highly conducting organic thermoelectric materials. This discrepancy suggests that structural inhomogeneities in thin films strongly influence the extracted transport parameters, highlighting the importance of the film integrity in thermoelectric optimization.

As a result of these measurement limitations, the calculated model curve deviates from our experimental data points, as shown in Figure 8. This deviation is expected because eq 6 is derived under the assumption of uniform carrier distribution, which is not the case in phase-separated or defective polymer layers. In materials with low electrical conductivity, the transport edge lies significantly above the Fermi level, meaning that only a small fraction of charge carriers participate in transport. The Kang–Snyder model accounts for this by incorporating a transport coefficient-dependent broadening effect, improving the accuracy of conductivity predictions in thermoelectric polymers. For P3HT:DBSA films, this effect explains the nonlinear scaling of conductivity with dopant addition, further justifying the use of the Kang et al. model over simpler linear approximations.

**Figure 8:**
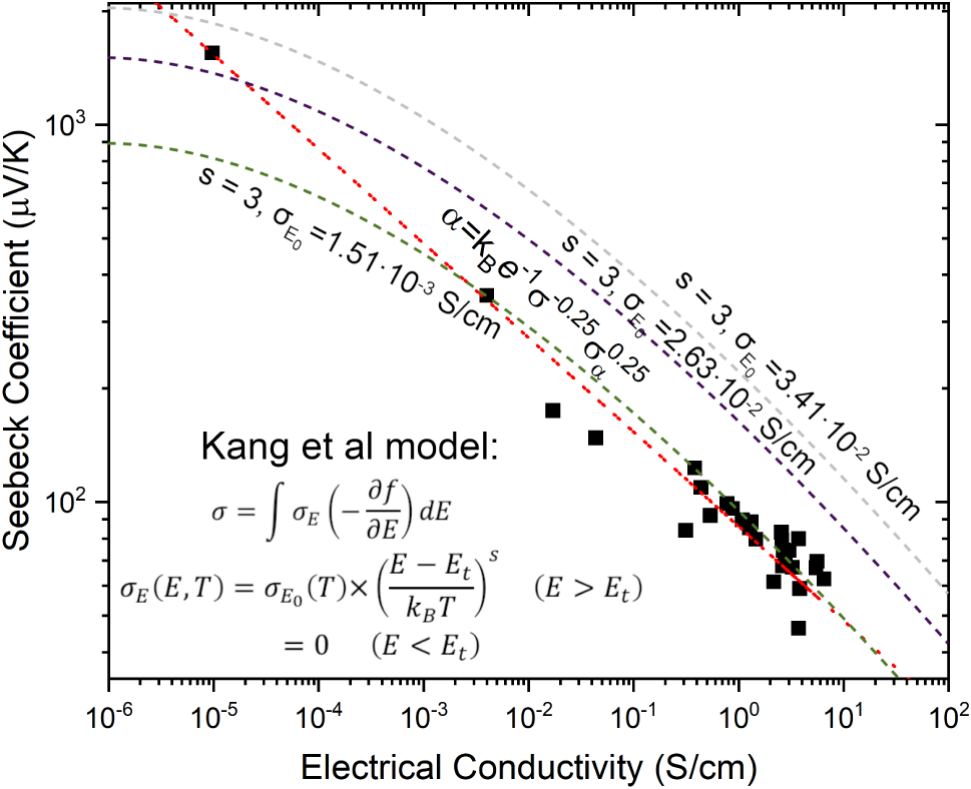
Calculation results of Kang et al. model (green, violet, and gray dashed lines) in comparison with Glaudell et al. model (red dashed line).

The Kang et al. model provides a powerful framework for predicting the optimal thermoelectric performance of conducting polymers by relating the transport coefficient, reduced chemical potential (η), and conductivity (σ) to the figure of merit (ZT). Since each P3HT:DBSA layer exhibits a different reduced chemical potential, the optimal ZT must be determined individually for each sample.

To accurately compute the maximum ZT for our system, we followed a multistep approach:

1. Experimental data for electrical conductivity (σ) and Seebeck coefficient (α) were used to determine the reduced chemical potential (η).
2. Seebeck coefficients were computed for η values ranging from −7 to 4, using a step size of 0.2.
3. A polynomial fit of the η­(α) function was applied, enabling us to extract precise reduced chemical potential values for each experimental point.
4. Transport coefficients $\left(\sigma_{E_{0}}\right)$ were computed using the following equation:^48^
7 $$\sigma_{E_{0}}=\sigma \cdot s^{-1}\cdot F_{s-1}^{-1}\left(\eta \right)$$

Where $F_{s-1}^{-1}\left(\eta \right)$ represents the inverse Fermi integral function for a given reduced chemical potential, *s* is the charge transport exponent, which distinguishes hopping vs band-like transport.

Once the transport coefficient was obtained, we computed the quality factor (*B*):^48^

8 $$B={\left(k_{B}/e\right)}^{2}\cdot \sigma_{E_{0}}\cdot T\cdot \kappa_{l}^{-1}$$

Where *T* is the room temperature (300 K), and κ_*l*_ is the lattice thermal conductivity, assumed as 0.2 W·m^–1^·K^–1^, a typical value for conjugated polymers.

The quality factor (*B*) is a crucial parameter in thermoelectrics, as it enables the calculation of optimal ZT values:^48^

9 $$ZT=\alpha^{2}\cdot \left({\left(k_{B}/e\right)}^{2}/\left(B\cdot s\cdot F_{s}\left(\eta \right)\right)+L\right)$$

where *L* is the Lorenz number, given by^48^

10 $$L={\left(k_{B}/e\right)}^{2}\cdot \left(s\left(s+2\right)F_{s-1}\left(\eta \right)F_{s+1}\left(\eta \right)-{\left(s+1\right)}^{2}F_{s}^{2}\left(\eta \right)\right)/\left(s^{2}F_{s-1}^{2}\left(\eta \right)\right)$$

For P3HT:DBSA, our calculations yielded an optimal ZT of 0.0045, corresponding to a 12.96:1 DBSA:3HT molar ratio. This suggests that the ideal thermoelectric performance is achieved at moderate DBSA doping levels, where the balance between carrier mobility and density of states broadening is optimized. Using the Kang et al. model, we were able to derive the relationship between Seebeck coefficient and conductivity, producing a curve similar to Figure 8, which distinguishes between degenerate and nondegenerate charge transport. The charge transport exponent (*s*) plays a crucial role in determining how well the model fits the experimental data. As shown in Figure 9, the fit improves as s increases, suggesting that our system transitions from hopping transport (low *s*) to delocalized transport (high *s*) as conductivity increases. It must be noted that the best fitting (*s* = 4) is only for degenerated transport.

**Figure 9:**
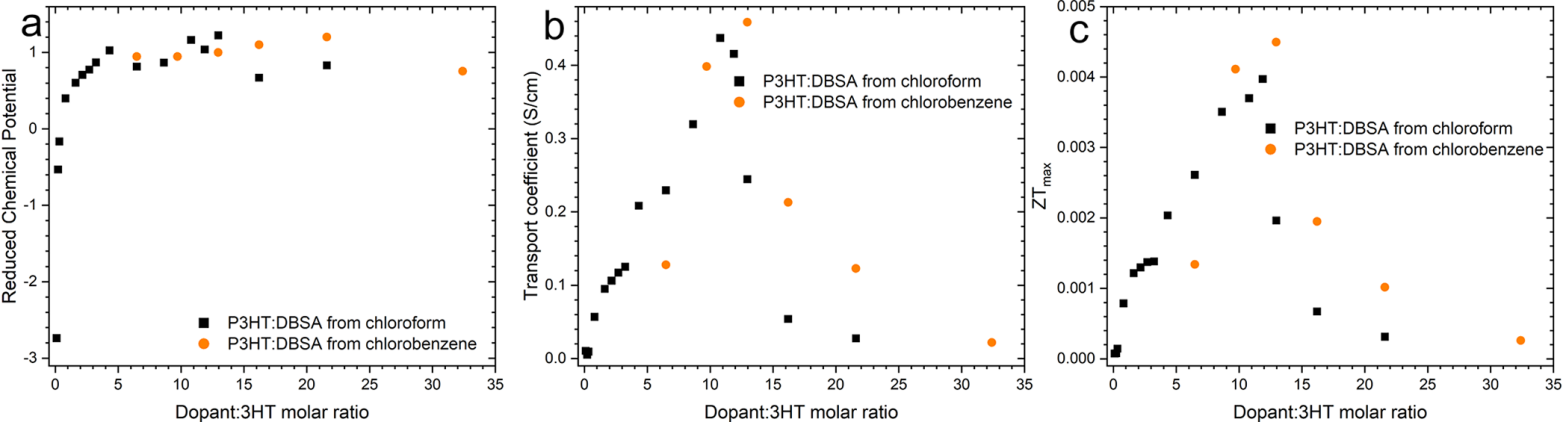
Calculation results with Kang et al. model of reduced chemical potential (a), transport coefficient (b), and optimal figure of merit (c) of our samples.

For degenerate transport, the Seebeck coefficient is as follows:

11 $$\alpha =k_{B}/e\cdot \pi^{2}/3\cdot s\cdot {\left(\sigma /\sigma_{E_{0}}\right)}^{-1/s}$$

By comparing Glaudell et al.‘s empirical model with Kang et al.‘s transport function, we converted eqs 2 and 11, leading to the conclusion that the best theoretical fit is obtained when $\sigma_{E_{0}}$ = 3.33 × 10^–5^ S·cm^–1^. Our experimental data align well with this prediction, confirming the validity of the Kang et al. model for DBSA-doped P3HT.

To address issues related to film cracking in spin-coated P3HT:DBSA layers, we investigated the use of chlorobenzene as an alternative solvent. Due to its higher boiling point (132 °C vs 61 °C for chloroform), chlorobenzene should reduce solvent evaporation rates, mitigating film shrinkage during drying. We conducted additional thermoelectric measurements on chlorobenzene-processed P3HT:DBSA films, with the results summarized in Table 2. Although this approach improved film uniformity, its impact on thermoelectric performance was minimal, suggesting that dopant-polymer interactions play a more dominant role in determining transport properties than solvent choice.

**Table 2:** Results of Thermoelectric Measurements of P3HT:DBSA-Based Devices Made from Chlorobenzene

Sample (3HT:dopant molar ratio)	Conductivity ***σ*** [S·cm^–1^]	Seebeck coefficient [μV·K^–1^]	Power factor ***σα* ^2^** [μW·m^–1^·K^–2^]
P3HT + DBSA (1:32.40)	0.23	86.8	0.17
P3HT + DBSA (1:21.60)	1.88	60.3	0.69
P3HT + DBSA (1:16.20)	3.014	66.1	1.32
P3HT + DBSA (1:12.96)	5.96	71.9	3.081
P3HT + DBSA (1:9.72)	4.95	75.2	2.80
P3HT + DBSA (1:6.48)	1.59	75.2	0.90

The substitution of chlorobenzene for chloroform as a solvent led to notable improvements in thin-film properties, particularly at higher DBSA doping levels. Unlike chloroform-processed films, which frequently exhibited cracking and phase separation at high acid concentrations, the chlorobenzene-based films remained intact, allowing for higher DBSA incorporation without film deterioration. The higher boiling point of chlorobenzene (132 °C vs 61 °C for chloroform) slows the solvent evaporation rate during spin coating, promoting better molecular ordering and reduced internal film stress. This effect has been observed in other solution-processed conjugated polymers, where solvent evaporation dynamics directly influence the crystallinity and phase purity. Additionally, studies by Vijayakumar et al. demonstrated that P3HT films cast from chlorobenzene exhibit higher crystallinity and better charge carrier mobility compared to those processed with chloroform. A similar trend was observed in our experiments, where the improved film morphology enabled higher doping levels without significant degradation. However, despite these morphological enhancements, the thermoelectric properties did not show significant improvement at the optimized P3HT:DBSA ratio. This suggests that solvent selection primarily affects the mechanical integrity and film stability, while thermoelectric efficiency is more dependent on the intrinsic electronic interactions between DBSA and P3HT chains. The improved film uniformity upon using chlorobenzene was confirmed by the absence of cracks and phase-separated regions during and after coating. This observation is consistent with previous reports showing that high-boiling-point solvents promote improved chain ordering and reduce morphological defects in P3HT films (Niefind et al., *Nanoscale Adv.*, 2019; Hynynen et al., *RSC Adv.*, 2018).^39,49^ A comparison of the electrical and thermoelectric properties of the different solvent-processed layers is presented in Figure 10.

**Figure 10:**
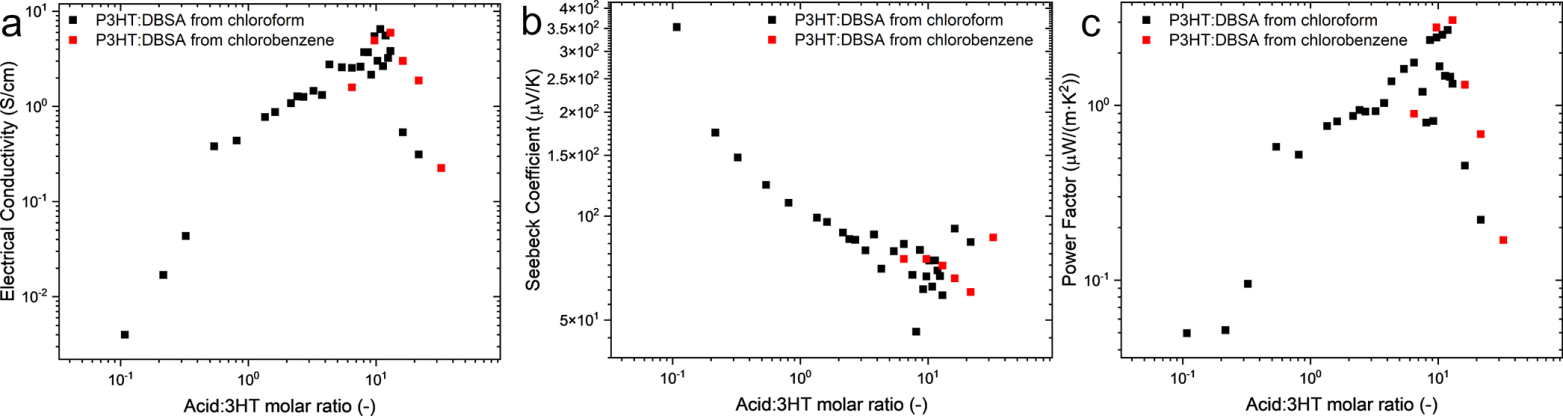
Thermoelectric parameter comparison between layers made from chloroform (black points) and chlorobenzene (red points). (a) Electrical conductivity, (b) Seebeck Coefficients, and (c) Power Factors per acid:3HT ratio in solid samples.

This is the first demonstration that solvent substitution directly mitigates DBSA-induced cracking, providing a simple processing strategy absent in earlier alkylbenzenesulfonic acid doping studies.

To complement the thin-film results, Table 3 summarizes the thermoelectric parameters of pelletized P3HT:DBSA samples fabricated by PECS and measured at 298 K. The general trend follows that of the thin films—an increase in σ with moderate DBSA addition, accompanied by a decrease in S, leading to an optimal power factor for intermediate doping levels. These results confirm that the charge transport and doping mechanisms observed in spin-coated films are preserved in bulk-like, consolidated samples (see Supporting Information, Tables S1–S3, for complete temperature-dependent data).

**Table 3:** Thermoelectric Parameters of P3HT:DBSA Pellets Prepared by Pulsed Electric Current Sintering (PECS), Measured at298 K

Pellet (mass ratio, sputtering time, and temperature)	Conductivity ***σ*** [S·cm^–1^]	Seebeck coefficient [μV·K^–1^]	Power factor ***σα* ^2^** [μW·m^–1^·K^–2^]	Thermal conductivity κ [μW·m^–1^·K^–1^]	ZT
P3HT:DBSA 1:0, 30 min, 120 °C	4.98 × 10^–5^	745	0.0028	0.259	3.208 × 10^–6^
P3HT:DBSA 10:1, 30 min, 120 °C	6.64 × 10^–4^	558	0.0207	0.226	9.62 × 10^–6^
P3HT:DBSA 2:1, 30 min, 120 °C	0.0079	357	0.101	0.186	1.63 × 10^–4^
P3HT:DBSA 1:1, 30 min, 120 °C	0.016	263	0.108	0.183	1.78 × 10^–4^
P3HT:DBSA 2:3, 30 min, 120 °C	0.103	145	0.22	0.180	3.62 × 10^–4^
P3HT:DBSA 1:5, 30 min, 120 °C	0.0029	235	0.016	0.157	3.063 × 10^–5^
P3HT:DBSA 1:10, 30 min, 120 °C	0.0022	265	0.016	0.142	3.303 × 10^–5^
P3HT:DBSA 2:3, 30 min, 90 °C	0.035	204	0.103	0.162	1.908 × 10^–4^
P3HT:DBSA 2:3, 30 min, 150 °C	0.0022	366	0.029	0.1708	5.16 × 10^–5^
P3HT:DBSA 2:3, 30 min, 180 °C	9.907 × 10^–4^	357	0.013	0.194	1.96 × 10^–5^
P3HT:DBSA 2:3, 10 min, 120 °C	6.23 × 10^–4^	534	0.018	0.117	4.56 × 10^–5^
P3HT:DBSA 2:3, 45 min, 120 °C	0.0106	270	0.078	0.238	9.805 × 10^–5^
P3HT:DBSA 2:3, 60 min, 120 °C	0.022	219	0.105	0.182	1.74 × 10^–4^

The overall consistency between the film and pellet results suggests that the electrical transport in both cases is governed by similar mechanisms, dominated by polaronic hopping and protonation-induced carrier generation.

To further quantify charge transport efficiency, we conducted a comparative analysis of the weighted mobility (μ_*w*_)^38–44^ of our P3HT:DBSA layers, using the methodology established by Snyder et al.^50^ This metric is crucial for evaluating how effectively charge carriers contribute to electrical conductivity, particularly in organic thermoelectric materials.

The weighted mobility (μ_*w*_) was calculated using

12 $$\mu_{w}=\sigma /e\cdot n_{eff}$$

Where σ is the electrical conductivity, *e* is the elementary charge, and *n*_eff_ is the effective charge carrier density.

A comparative analysis of our weighted mobility values compared to those reported in the literature is shown in Figure 11. Notably, our P3HT:DBSA layers exhibited higher weighted mobility than standard P3HT:PCBM (phenyl-C61-butyric acid methyl ester) blends, widely used in organic photovoltaics (OPVs). For reference, charge mobility in P3HT:PCBM systems typically ranges from 10^–5^ and 10^–3^ cm^2^·V^–1^·s^–1^, while our P3HT:DBSA layers demonstrated significantly higher values.^51^

**Figure 11:**
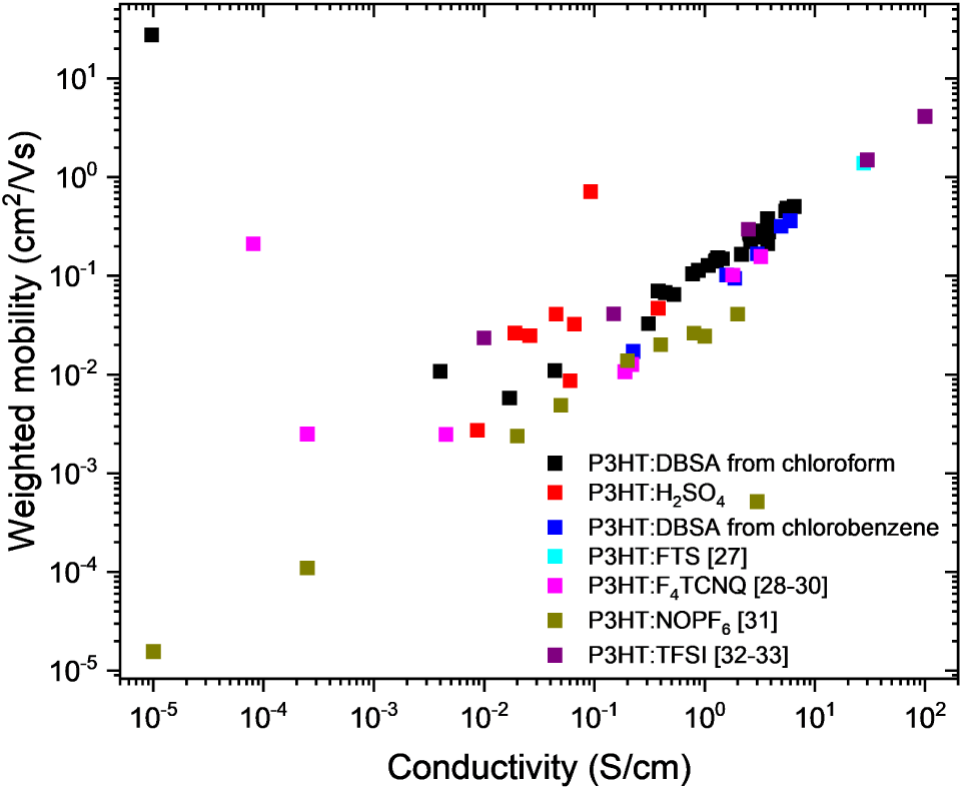
Comparison of P3HT-based materials’ weighted mobilities.

This improvement is attributed to enhanced carrier delocalization and reduced trap-state density, which result from DBSA’s ability to improve polymer chain ordering. The observed increase in the weighted mobility underscores the effectiveness of DBSA as a doping agent and highlights its potential for organic thermoelectric applications.

## Conclusion

We demonstrated the efficiency of dodecylbenzenesulfonic acid (DBSA) as an effective p-type dopant for poly­(3-hexylthiophene) (P3HT), offering an economical and less toxic alternative to the widely used F4TCNQ. Our comprehensive thermoelectric analysis highlighted the crucial role of the DBSA concentration in key thermoelectric properties, including the Seebeck coefficient, electrical conductivity, and power factor. The optimal P3HT:DBSA ratio was identified at 11.88% v/v (DBSA:3HT molar ratio of 12.96) DBSA, where the Seebeck coefficient, electrical conductivity, and power factor were 69.7 μV·K^–1^, 5.58 S·cm^–1^, and 2.706 μW·m^–1^·K^–2^, respectively. However, at higher DBSA concentrations (>15% v/v—DBSA:3HT molar ratio of ca. 16), film degradation occurred due to DBSA aggregation and phase separation, adversely affecting thermoelectric performance. Despite this, theoretical modeling suggested a maximum power factor of 4.00 μW·m^–1^·K^–2^ at a DBSA:3HT molar ratio of 12.96.

Morphological analysis using Atomic Force Microscopy (AFM) and Scanning Electron Microscopy (SEM) confirmed that DBSA doping reduced surface roughness, promoting more uniform thin-film formation. However, excessive doping introduced structural defects and charge transport disruptions, leading to a diminished performance at higher concentrations. The use of chlorobenzene instead of chloroform as a solvent improved the mechanical stability of the films, allowing for higher DBSA incorporation without significant cracking. Nevertheless, thermoelectric properties remained largely unchanged, indicating that solvent choice primarily influences film stability rather than the charge transport efficiency. To better understand charge transport mechanisms in DBSA-doped P3HT, we applied the charge transport model by Kang et al., which provided a more comprehensive theoretical fit compared to the empirical Mateeva et al. correlation, as the latter assumes a linear relationship between dopant concentration and conductivity. The calculated transport coefficient of 1.51 × 10^–3^ S·cm^–1^ aligned well with experimental results. Additionally, comparison with Glaudell et al.‘s empirical model further confirmed that DBSA-doped P3HT exhibits expected transport behavior, consistent with other acid-doped conducting polymers.

The findings of this study confirm that DBSA is a scalable and effective p-type dopant for P3HT, offering thermoelectric performance comparable to that of conventional dopants while being more cost-effective and less toxic. By addressing the remaining challenges in film stability and charge transport, DBSA-doped P3HT could become a promising candidate for next-generation flexible and sustainable thermoelectric applications. Future research should focus on enhancing the charge carrier mobility, optimizing thin-film deposition techniques, and integrating P3HT:DBSA into scalable thermoelectric modules. By reframing DBSA as not only an alternative dopant but also a platform for process optimization and transport analysis, this work establishes a methodological benchmark for future studies of acid-doped organic thermoelectrics. These advancements could pave the way for practical organic thermoelectric devices for energy harvesting and wearable electronics.

## Supplementary Material



## Notes

Experimental details; Scanning Electron Microscopy (SEM) images of P3HT and P3HT:DBSA pellets; Thermoelectric measurement data tables at 298, 343, and 388 K; Figures showing temperature-dependent electrical conductivity, Seebeck coefficient, and power factor; Additional plots illustrating effects of sputtering temperature, sputtering time, and DBSA concentration on thermoelectric performance (PDF)

## References

[1] Velmre E. (2007). Thomas Johann
Seebeck (1770–1831). Proc. Est. Acad.
Sci., Eng.

[2] Seebeck, T. J. Akademie der Wissenschaftten aus den Jahren 1820–1821 Academy of Sciences 1822 289

[3] Seebeck T. J. (1826). Ueber die
magnetische Polarisation der Metalle und Erze durch Temperatur-Differenz. Ann. Phys.

[4] Peltier, J. C. A. Nouvelles Expériences sur la Caloricité des courans électriques Annales de Chimie et de Physique 1834 371–386

[5] Webster, J. G. ; Gurevich, Y. U. G. ; Velazquez-Perez, J. E. Peltier Effect in Semiconductors. In Wiley Encyclopedia of Electrical and Electronics Engineering; Wiley, 2014; pp. 1–21.

[6] Skrabek, E. A. ; McGrew, J. W. Pioneer 10 and 11 RTG performance update Space Nuclear Power Systems, Transactions of the Fourth Symposium on Space Nuclear Power Systems New Mexico Univ 1987 201–204

[7] Ioffe, A. F. Semiconductor Thermoelements and Thermo-Electric Cooling; Infosearch Limited: London, 1957; p 184.

[8] Salaneck W. R., Inganäs O., Thémans B., Nilsson J. O., Sjögren B., Österholm J. E., Brédas J. L., Svensson S. (1988). Thermochromism in poly­(3-hexylthiophene)
in the solid state: A spectroscopic study of temperature-dependent
conformational defects. J. Chem. Phys..

[9] Aziz E. F., Vollmer A., Eisebitt S., Eberhardt W., Pingel P., Neher D., Koch N. (2007). Localized
charge transfer
in a molecularly doped conducting polymer. Adv.
Mater..

[10] Glaudell A. M., Cochran J. E., Patel S. N., Chabinyc M. L. (2015). Impact of the Doping
Method on Conductivity and Thermopower in Semiconducting Polythiophenes. Adv. Energy Mater..

[11] Hwang S., Potscavage W. J. J., Nakamichi R., Adachi C. (2016). Processing and Doping
of Thick Polymer Active Layers for Flexible Organic Thermoelectric
Modules. Org. Electron..

[12] Ossila MSDS of F4TCNQ https://downloads.ossila.com/msds/f4tcnq.pdf (Accessed Feb 13, 2025).

[13] Alveroglu E. (2015). Doping effect
of dodecyl benzene sulphonic acid in poly­(3-hexylthiophene)-P3HT-films. J. Mol. Struct..

[14] Sigma-Aldrich. Safety Data Sheet of 4-Dodecylbenzenesulfonic acid https://www.sigmaaldrich.com/PL/en/sds/aldrich/44198. (Accessed Feb 13, 2025).

[15] Bonardd S., Morales N., Gence L., Saldias C., Angel F. A., Kortaberria G., Leiva A. (2020). Doped Poly­(3-hexylthiophene) Coatings
onto Chitosan: A Novel Approach for Developing a Bio-Based Flexible
Electronic. ACS Appl. Mater. Interfaces.

[16] Shahrim N. A., Ahmad Z., Wong Azman A., Fachmi Buys Y., Sarifuddin N. (2021). Mechanisms for doped PEDOT: PSS electrical conductivity
improvement. Mater. Adv..

[17] Ouyang J. (2013). “Secondary
doping” methods to significantly enhance the conductivity of
PEDOT: PSS for its application as transparent electrode of optoelectronic
devices. Displays.

[18] Zhang L., Yang K., Chen R., Zhou Y., Chen S., Zheng Y., Li M., Xu C., Tang X., Zang Z. (2020). The Role of Mineral
Acid Doping of PEDOT: PSS and Its
Application in Organic Photovoltaics. Adv. Electron.
Mater..

[19] Hu Z., Zhang J., Hao Z., Zhao Y. (2011). Influence of doped
PEDOT: PSS on the performance of polymer solar cells. Sol. Energy Mater. Sol. Cells.

[20] Wu F., Li P., Sun K., Zhou Y., Chen W., Fu J., Li M., Lu S., Wei D., Tang X. (2017). Conductivity
Enhancement of PEDOT: PSS via Addition of Chloroplatinic Acid and
Its Mechanism. Adv. Electron. Mater..

[21] Chin Y., Daboczi M., Henderson C., Luke J., Kim J. (2022). Suppressing
PEDOT: PSS Doping-Induced Interfacial Recombination Loss in Perovskite
Solar Cells. ACS Energy Lett..

[22] Li J., Liu J., Gao C., Zhang J., Sun H. (2009). Influence of MWCNTs
doping on the structure and properties of PEDOT: PSS films. Int. J. Photoenergy.

[23] Mukherjee S., Singh R., Gopinathan S., Murugan S., Gawali S., Saha B., Biswas J., Lodha S., Kumar A. (2014). Solution-processed
poly­(3,4-ethylenedioxythiophene) thin films as transparent conductors:
Effect of p-toluenesulfonic acid in dimethyl sulfoxide. ACS Appl. Mater. Interfaces.

[24] Suh E. H., Oh J. G., Jung J., Noh S. H., Lee T. S., Jang J. (2020). Brønsted Acid
Doping of P3HT with Largely Soluble Tris­(pentafluorophenyl)­borane
for Highly Conductive and Stable Organic Thermoelectrics Via One-Step
Solution Mixing. Adv. Energy Mater..

[25] Villalva D. R., Haque M. A., Nugraha M. I., Baran D. (2020). Enhanced Thermoelectric
Performance and Lifetime in Acid-Doped PEDOT: PSS Films Via Work Function
Modification. ACS Appl. Mater. Interfaces.

[26] Patel S. N., Glaudell A. M., Kiefer D., Chabinyc M. L. (2016). Increasing the Thermoelectric
Power Factor of a Semiconducting Polymer by Doping from the Vapor
Phase. ACS Macro Lett..

[27] Tumova Š., Maleckova R., Kubac L., Akrman J., Enev V., Kalin L., Vojtkova E., Peskova M., Vitecek J., Vala M. (2023). Novel highly stable conductive polymer composite PEDOT:
DBSA for bioelectronic applications. Polym.
J..

[28] Horta-Romaris L., González-Rodríguez M. V., Lasagabaster A., Rivadulla F., Abad M.-J. (2018). Thermoelectric properties
and intrinsic
conduction processes in DBSA and NaSIPA doped polyanilines. Synth. Met..

[29] Malzbender, T. ; Gelb, D. ; Wolters, H. Polynomial Texture Maps. https://www.hpl.hp.com/research/ptm/papers/ptm.pdf. (Accessed Sep 1, 2025).

[30] Gáspár S., Ravasenga T., Munteanu R.E., David S., Benfenati F., Colombo E. (2021). Electrochemically Synthesized Poly (3-hexylthiophene)
Nanowires as Photosensitive Neuronal Interfaces. Materials.

[31] Perry S., Arumugam S., Beeby S., Nandhakumar I. S. (2023). Template-Free
Nanostructured Poly­(3-hexylthiophene) Films via Single Pulse-Nucleated
Electrodeposition. J. Electroanal. Chem..

[32] Mizuno M., Tateno H., Matsumura Y., Atobe M. (2017). Synthesis and Molecular
Weight Control of Poly­(3-hexylthiophene) via Electrochemical Polymerization
in a Flow Microreactor. React. Chem. Eng..

[33] Ma B. S., Lee J. W., Park H., Kim B. J., Kim T. S. (2022). Thermomechanical
Behavior of Poly­(3-hexylthiophene) Thin Films on the Water Surface. ACS Omega.

[34] Bonetti M., Nakamae S., Roger M., Guenoun P. (2011). Huge Seebeck coefficients
in nonaqueous electrolytes. J. Chem. Phys..

[35] Iwanaga S., Toberer E. S., LaLonde A., Snyder G. J. (2011). A high temperature
apparatus for measurement of the Seebeck coefficient. Rev. Sci. Instrum..

[36] Zhang Y., Park S.-J. (2019). Flexible Organic
Thermoelectric Materials and Devices
for Wearable Green Energy Harvesting. Polymers.

[37] Kroon R., Mengistie D. A., Kiefer D., Hynynen J., Ryan J. D., Yu L., Muller C. (2016). Thermoelectric plastics: From design to synthesis,
processing and structure-property relationships. Chem. Soc. Rev..

[38] Zou Y., Huang D., Meng Q., Di C., Zhu D. (2018). Correlation
between Seebeck coefficient and transport energy level in poly­(3-hexylthiophene). Org. Electron..

[39] Hynynen J., Kiefer D., Müller C. (2018). Influence
of crystallinity on the
thermoelectric power factor of P3HT vapour-doped with F4TCNQ. RSC Adv..

[40] Hamidi-Sakr A., Biniek L., Bantignies J. L., Maurin D., Herrmann L., Leclerc N., Lévêque P., Vijayakumar V., Zimmermann N., Brinkmann M. (2017). A Versatile
Method to Fabricate Highly
In-Plane Aligned Conducting Polymer Films with Anisotropic Charge
Transport and Thermoelectric Properties: The Key Role of Alkyl Side
Chain Layers on the Doping Mechanism. Adv. Funct.
Mater..

[41] Kroon R., Ryan J. D., Kiefer D., Yu L., Hynynen J., Olsson E., Müller C. (2017). Bulk Doping
of Millimeter-Thick Conjugated
Polymer Foams for Plastic Thermoelectrics. Adv.
Funct. Mater..

[42] Zhang Q., Sun Y., Xu W., Zhu D. (2014). What to expect from conducting polymers
on the playground of thermoelectricity: Lessons learned from four
high-mobility polymeric semiconductors. Macromolecules.

[43] Qu S., Yao Q., Wang L., Chen Z., Xu K., Zeng H., Shi W., Zhang T., Uher C., Chen L. (2016). Highly anisotropic
P3HT films with enhanced thermoelectric performance via organic small
molecule epitaxy. NPG Asia Mater..

[44] Zhang Q., Sun Y., Xu W., Zhu D. (2012). Thermoelectric energy from flexible
P3HT films doped with a ferric salt of triflimide anions. Energy Environ. Sci..

[45] Miccoli I., Edler F., Pfnür H., Tegenkamp C. (2015). The 100th
anniversary of the four-point probe technique: the role of probe geometries
in isotropic and anisotropic systems. J. Phys.:
Condens. Matter.

[46] Yusupov K., Vomiero A. (2020). Polymer-Based Low-Temperature
Thermoelectric Composites. Adv. Funct. Mater..

[47] Mateeva N., Niculescu H., Schlenoff J., Testardi L. R. (1998). Correlation of Seebeck
coefficient and electric conductivity in polyaniline and polypyrrole. J. Appl. Phys..

[48] Kang S. D., Snyder G. J. (2017). Charge-transport
model for conducting polymers. Nat. Mater..

[49] Niefind F., Karande S., Frost F., Abel B., Kahnt A. (2019). Solvent Influence
on the Surface Morphology of P3HT Thin Films Revealed by Photoemission
Electron Microscopy. Nanoscale Adv..

[50] Snyder G.
J., Snyder A. H., Wood M., Gurunathan R., Snyder B. H., Niu C. (2020). Weighted Mobility. Adv. Mater..

[51] Kaku K., Williams A. T., Mendis B. G., Groves C. (2015). Examining charge transport
networks in organic bulk heterojunction photovoltaic diodes using
1/f noise spectroscopy. J. Mater. Chem. C.

