# Supplementary material for: Step towards High Power Factor in Acidic-Doped Poly(3-hexylthiophene) Systems
Source: ACS Omega. 2025 Dec 15;10(51):63336–48. doi: 10.1021/acsomega.5c09899 (PMC12756752; doi:10.1021/acsomega.5c09899)
Supplement: Supplementary file 1 [file ao5c09899_si_001.pdf]

Step Towards High Power Factor in Acidic Doped Poly(3-hexylthiophene) Systems

Szymon Gogoc,[a] Pawel Gnida,[b] Anna Adamczyk, [c] Aleksandra Wypych-Puszkarz,[d] Krzysztof Wojciechowski *[a] and Przemyslaw Data *[d,e]*

[a] Department of Inorganic Chemistry, Faculty of Materials Science and Ceramics, AGH University of Krakow, Mickiewicza 30, 30-059 Kraków, Poland, E-mail: sgogoc@agh.edu.pl; wojciech@agh.edu.pl

[b] Centre of Polymer and Carbon Materials, Polish Academy of Sciences, Marii Sklodowskiej-Curie 34, 41-819 Zabrze, Poland

[c] Department of Silicate Chemistry and Macromolecular Compounds, Faculty of Materials Science and Ceramics, AGH University of Kraków, Mickiewicza 30, 30-059 Cracow, Poland

[d] Department of Molecular Physics, Faculty of Chemistry, Lodz University of Technology, Zeromskiego 116, 90-924 Lodz, Poland, E-mail: przemyslaw.data@p.lodz.pl

[e] Department of Physics, Durham University, South Road, Durham, UK, E-mail: przemyslaw.r.data@durham.ac.uk

Supporting information for this article is given via a link at the end of the document.

Table of Contents

1. Experimental part

2. Scanning Electron Microscopy results

3. Measurements of ZT

4. Thermoelectric properties

1. Experimental part

Materials and Sample Preparation

Poly(3-hexylthiophene) (P3HT) (M1011, Mw = 61,500) was purchased from Ossila and used as received without further purification. The dopant dodecylbenzenesulfonic acid (DBSA) (mixture of isomers, purity ≥95%) was obtained from Sigma-Aldrich. Both materials were stored in an inert environment to prevent degradation before use.

To prepare the polymer solutions, P3HT was dissolved in chloroform at a concentration of 20 mg/mL. Various amounts of DBSA were then added to the solutions to achieve different P3HT:DBSA mass ratios, ranging from pristine P3HT (undoped) to a 1:10 P3HT:DBSA ratio. The solutions were stirred for at least 1 hour to ensure homogeneity before further processing.

After thorough mixing, the solutions were dried under vacuum conditions at 50°C for 24 hours to remove residual solvent and promote film formation. The structures of both P3HT and DBSA are shown in Figure 2, illustrating the chemical interactions that occur upon doping.

Pellet Fabrication via Pulsed Electron Current Sintering (PECS)

The dried polymer films were subjected to Pulsed Electron Current Sintering (PECS) to enhance their structural integrity and thermoelectric properties. The sintering process was conducted under an inert argon (Ar) atmosphere to prevent oxidation and degradation of the polymer.

To evaluate the effects of temperature and duration of sintering, the process was performed under the following conditions:

- Sintering temperatures: 90°C, 120°C, 150°C, and 180°C.
- Sintering durations: 10, 30, 45, and 60 minutes.
- Applied pressure: 35 MPa.

After sintering, the thickness of the pellets was measured using a micrometer, ensuring consistency across all samples before characterization.

Structural and Morphological Characterization

Scanning Electron Microscopy (SEM) Analysis

To investigate the morphology and microstructural changes induced by DBSA doping and PECS treatment, Scanning Electron Microscopy (SEM) images were obtained using a NOVA NANO SEM 200. Cross-sectional imaging was performed using both the Everhart–Thornley Detector (ETD) and the T2 in-lens detector to analyze the internal structure and uniformity of the films. Differences in morphology between pristine P3HT and DBSA-doped samples were observed, particularly regarding surface roughness and phase separation at high dopant concentrations.

Infrared Spectroscopy (IR) Analysis

The chemical interactions between P3HT and DBSA were analyzed using Fourier Transform Infrared Spectroscopy (FTIR) with a BIO-RAD FTS6000 instrument. The presence of sulfonic acid groups from DBSA and their interaction with the P3HT backbone were confirmed by identifying characteristic absorption peaks. These data provide insight into the molecular-level modifications caused by DBSA doping.

Thermoelectric Measurements

Electrical Conductivity and Seebeck Coefficient

The electrical conductivity (σ) and Seebeck coefficient (S) of the P3HT:DBSA samples were measured using a Netzsch SBA 458 Nemesis setup. The measurements were conducted under an argon atmosphere over a temperature range of 25°C to 115°C to analyze the temperature dependence of the thermoelectric properties. The power factor ($PF = \sigma S^2$) was calculated for each sample to determine the optimal doping conditions.

Thermal Conductivity and Figure of Merit (ZT)

The thermal conductivity (κ) of the samples was measured using a Netzsch LFA 457 Micro Flash instrument. Based on these measurements, the thermoelectric figure of merit ($ZT = (\sigma S^2 T)/\kappa$) was determined for each P3HT:DBSA ratio at various temperatures. The resulting data were used to compare the thermoelectric performance of DBSA-doped P3HT with other commonly used dopants, such as sulfuric acid and F4TCNQ.

Optimization of P3HT:DBSA Thermoelectric Performance

To identify the most effective doping conditions, the influence of DBSA concentration, sintering temperature, and processing time on electrical conductivity, Seebeck coefficient, and power factor was systematically studied. Higher doping concentrations resulted in an increase in electrical conductivity but also introduced morphological changes that influenced charge transport. The correlation between these structural modifications and thermoelectric efficiency was examined using AFM, SEM, and FTIR analysis.

Ultimately, the thermoelectric performance of P3HT:DBSA was evaluated against existing literature values for sulfuric acid and F4TCNQ-doped P3HT, highlighting the potential of DBSA as a cost-effective, scalable, and environmentally friendly alternative.

Experimental setup was based on Netzsch SBA 458 Nemesis setup for thermoelectric measurements and computer with LabView environment for obtaining raw data. Scanning Electron Microscopy (SEM) graphs were obtained by NOVA NANO SEM 200. Infrared Spectroscopy was made with BIO-RAD FTS6000 with proper software. Thermal conductivity has been measured with Netzsch LFA 457 Micro Flash. P3HT (M1011, Mw = 61,500 bought from Ossila) was dissolved in chloroform, concentration of solution was 20 mg/mL. DBSA (mixture of isomers, ≥95%, bought from Sigma-Aldrich) was added into prepared solution. Structures of mentioned compound are shown on **Figure 2**. Additions of dopant in solution were between pure P3HT and 1:10 P3HT:DBSA mass ratio. After mixing the solution, mixture has dried in vacuum dryer for 24 hours in 50 ºC of temperature. After drying process, sample has been sputtered with pulsed electron current sintering (PECS) technique by 10, 30, 45 and 60 minutes in 90, 120, 150 and 180 ºC with 35 MPa of pressure. All PECS synthesis were made in inert gas (argon). Thickness of samples have been measured with micrometer. After measurement, devices were undertaken morphology and electrical analysis with Infrared Spectroscopy (IR), and I-V thermoelectric technique. Thermoelectric measurements were made between 25 and 115 ºC of temperature in argon.

2. Scanning Electron Microscopy results

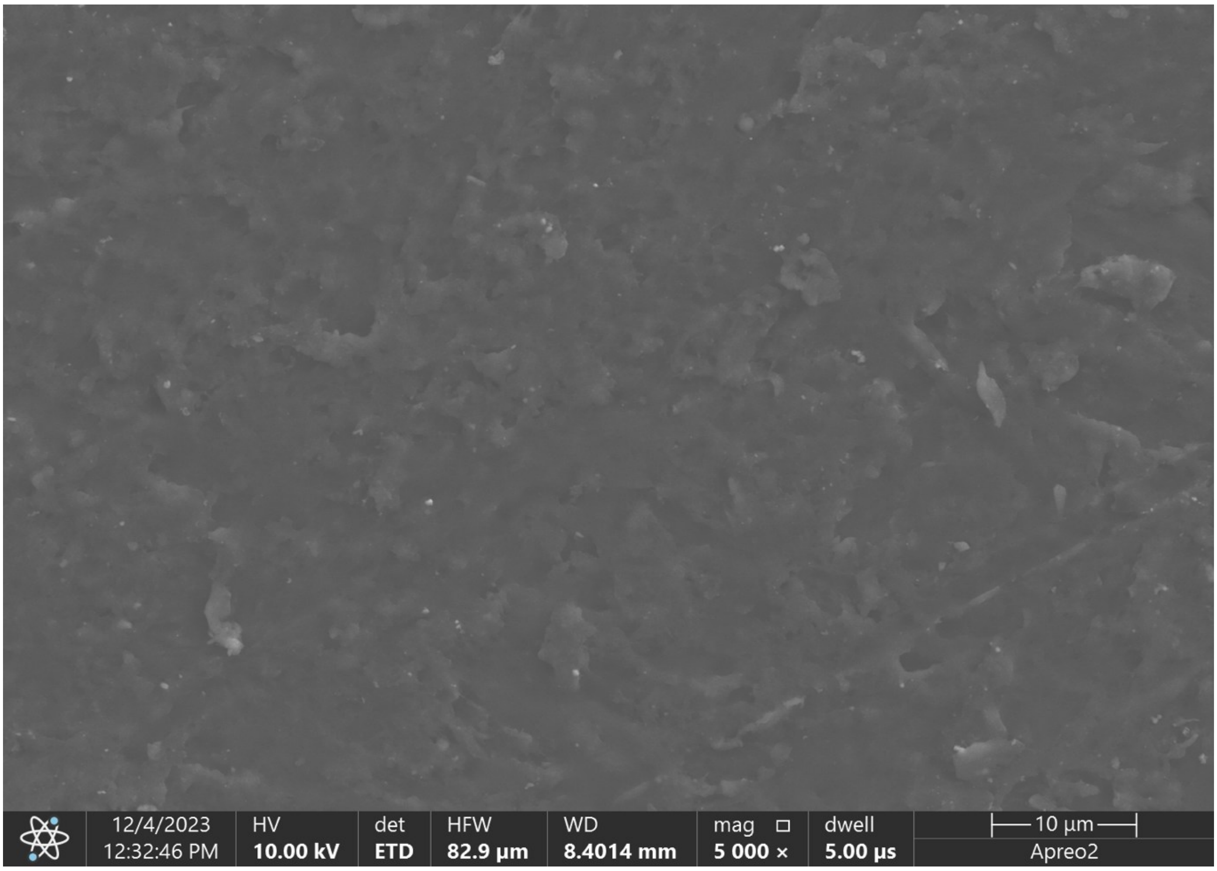

Figure S1. SEM graph of P3HT pellet cross-section (Everhart–Thornley detector – ETD)

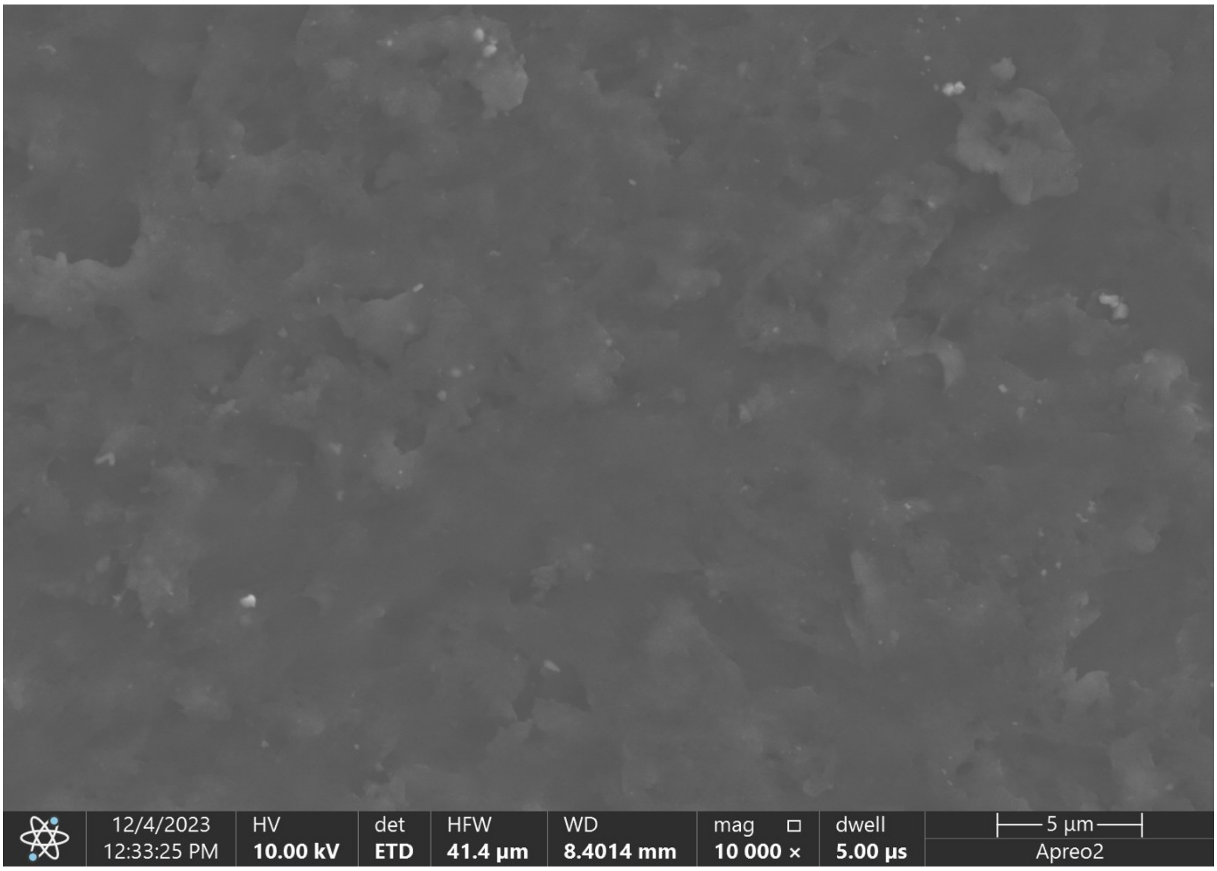

Figure S2. SEM graph of P3HT pellet cross-section (ETD)

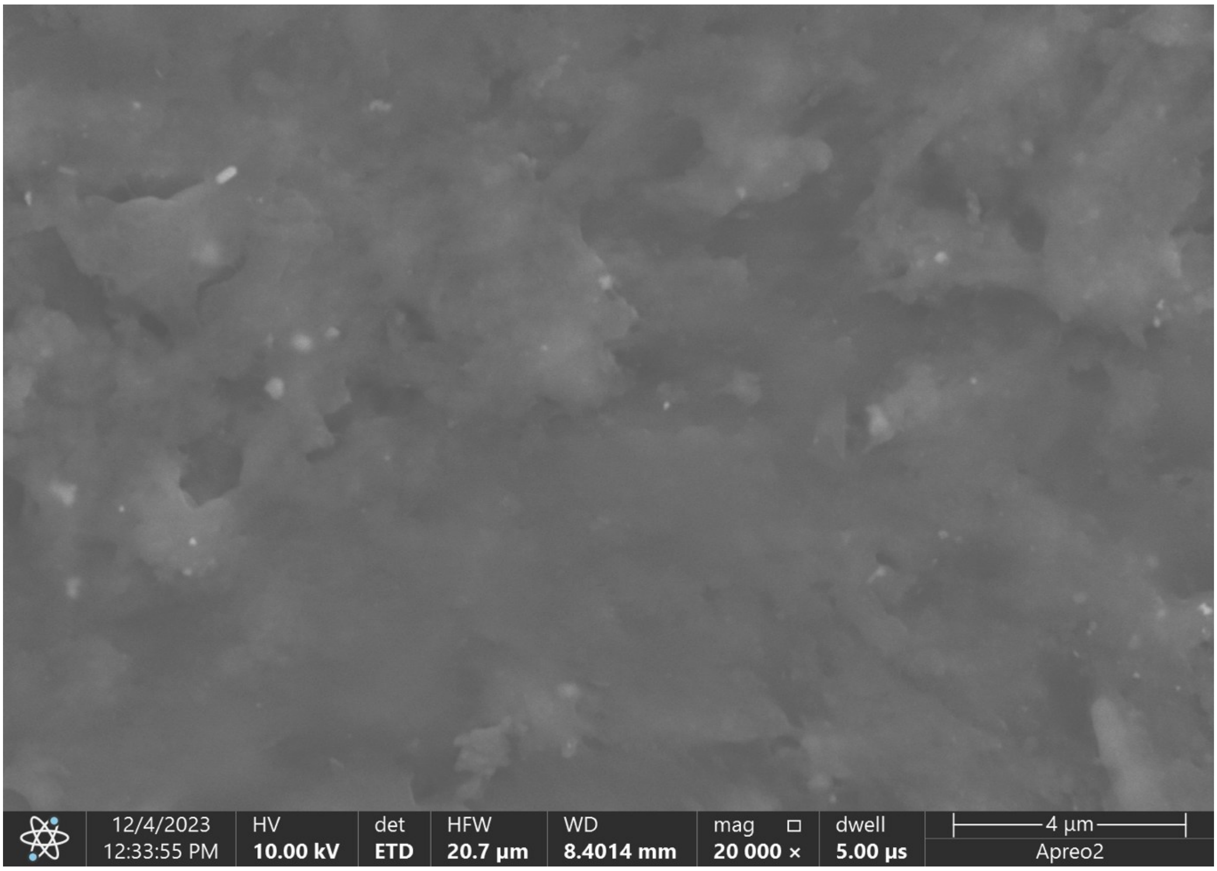

Figure S3. SEM graph of P3HT pellet cross-section (ETD)

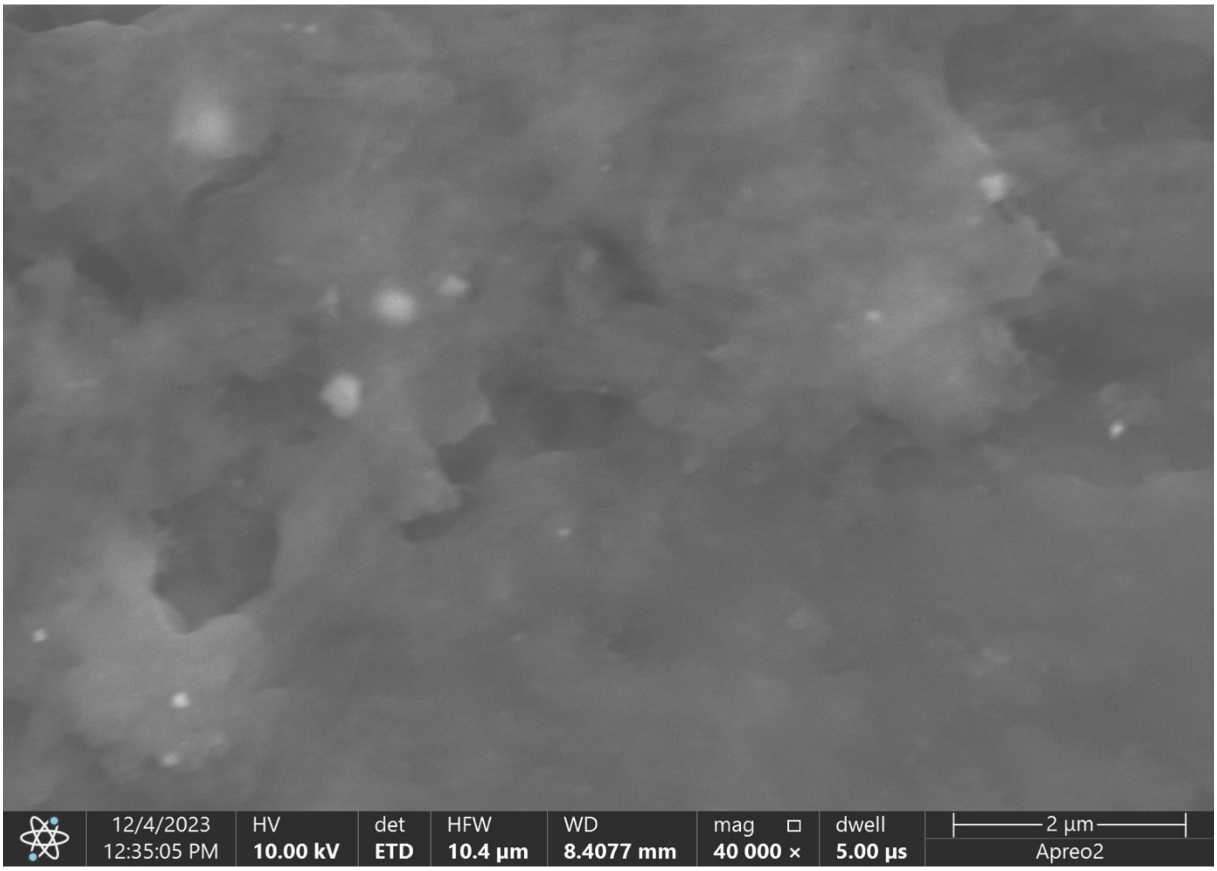

Figure S4. SEM graph of P3HT pellet cross-section (ETD)

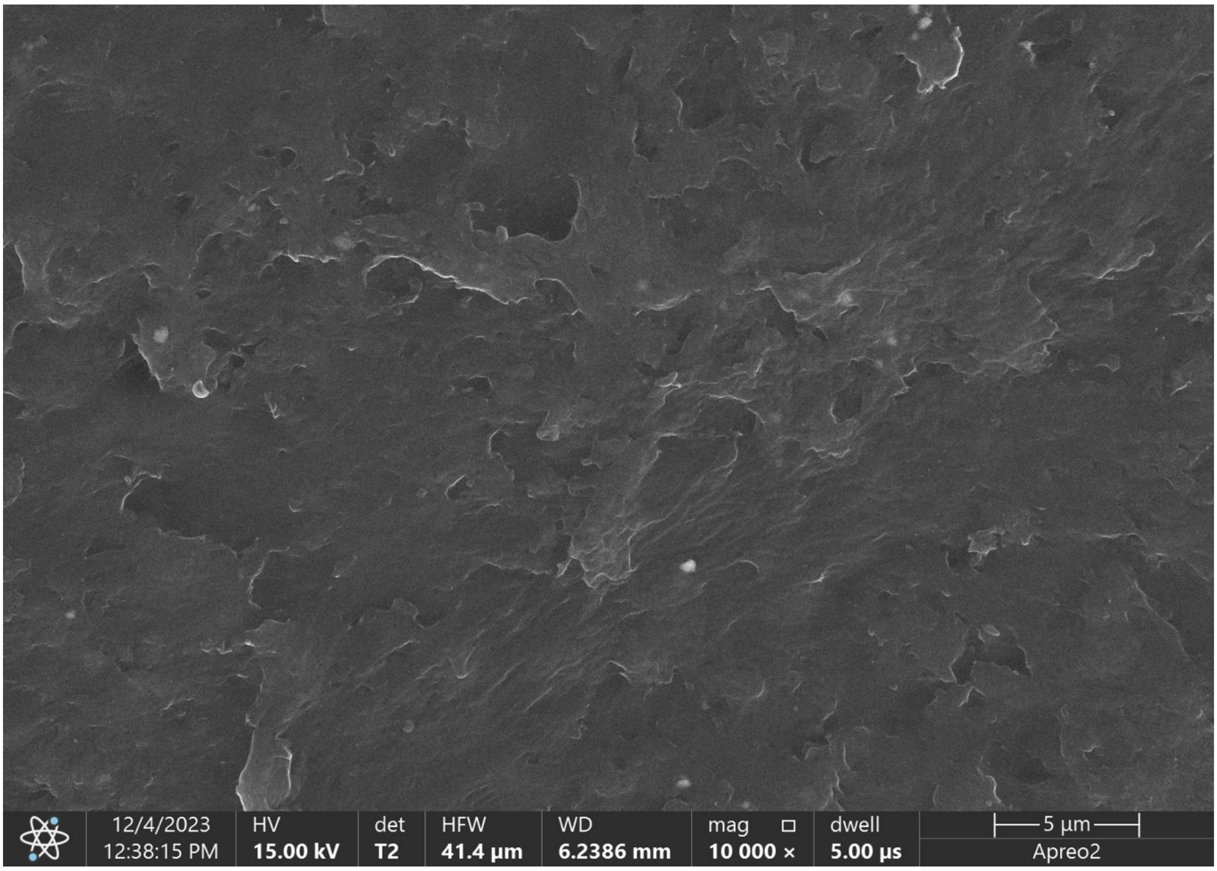

Figure S5. SEM graph of P3HT pellet cross-section (T2 in-lens detector)

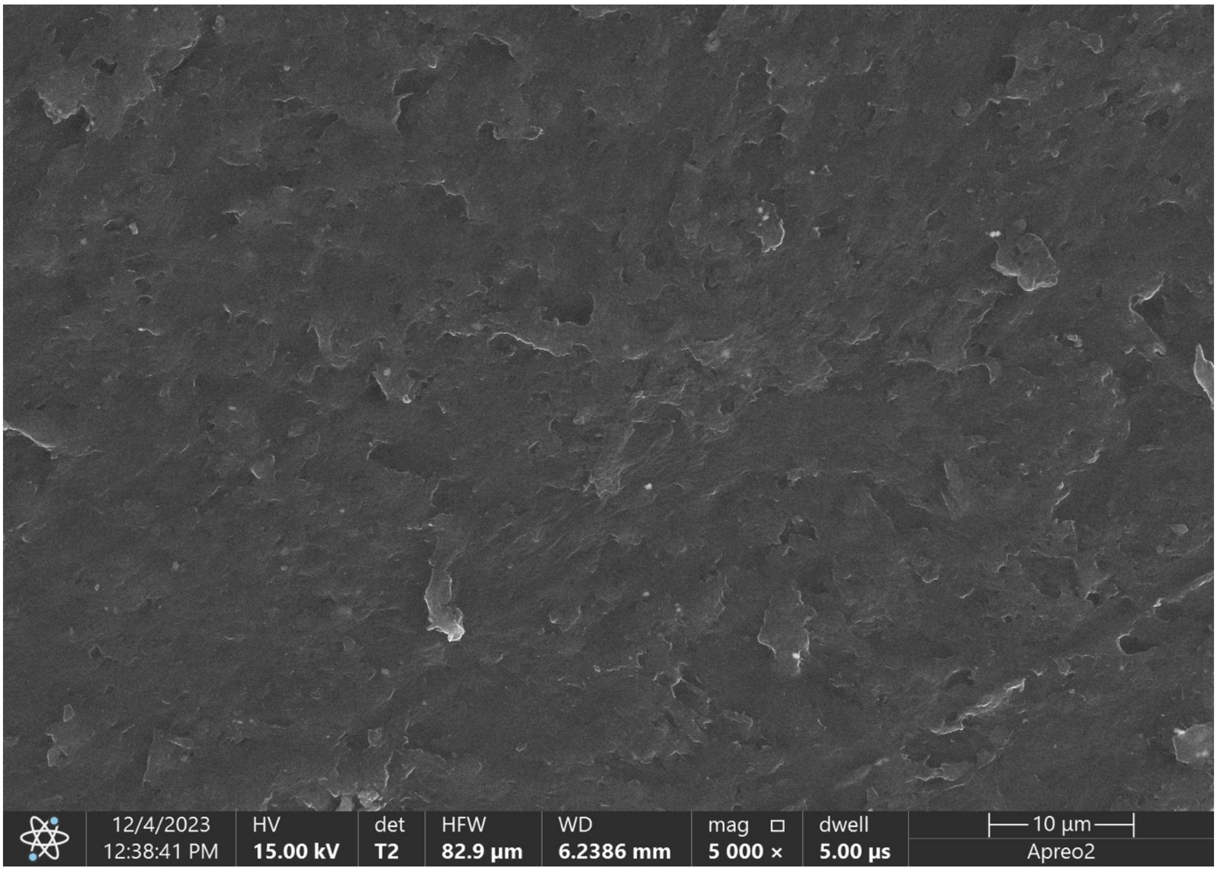

Figure S6. SEM graph of P3HT pellet cross-section (T2)

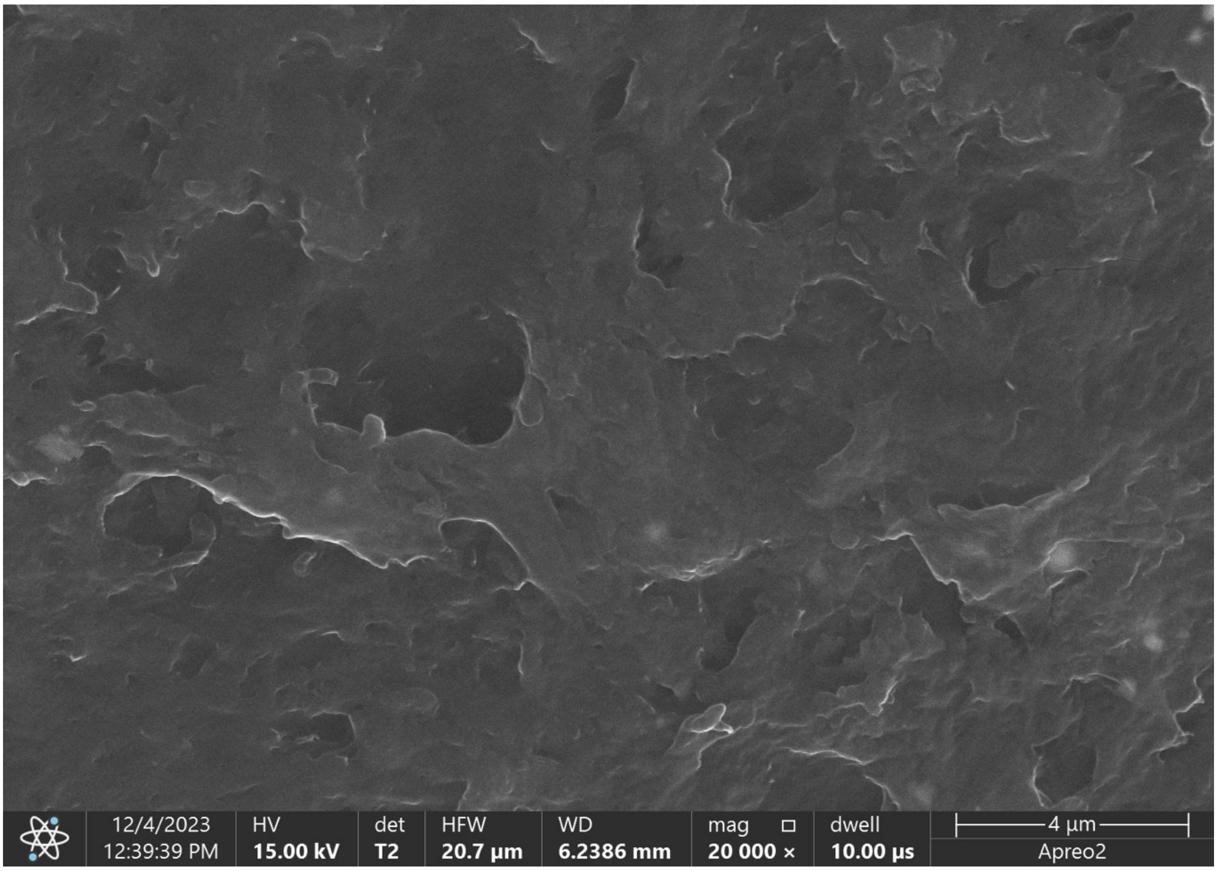

Figure S7. SEM graph of P3HT pellet cross-section (T2)

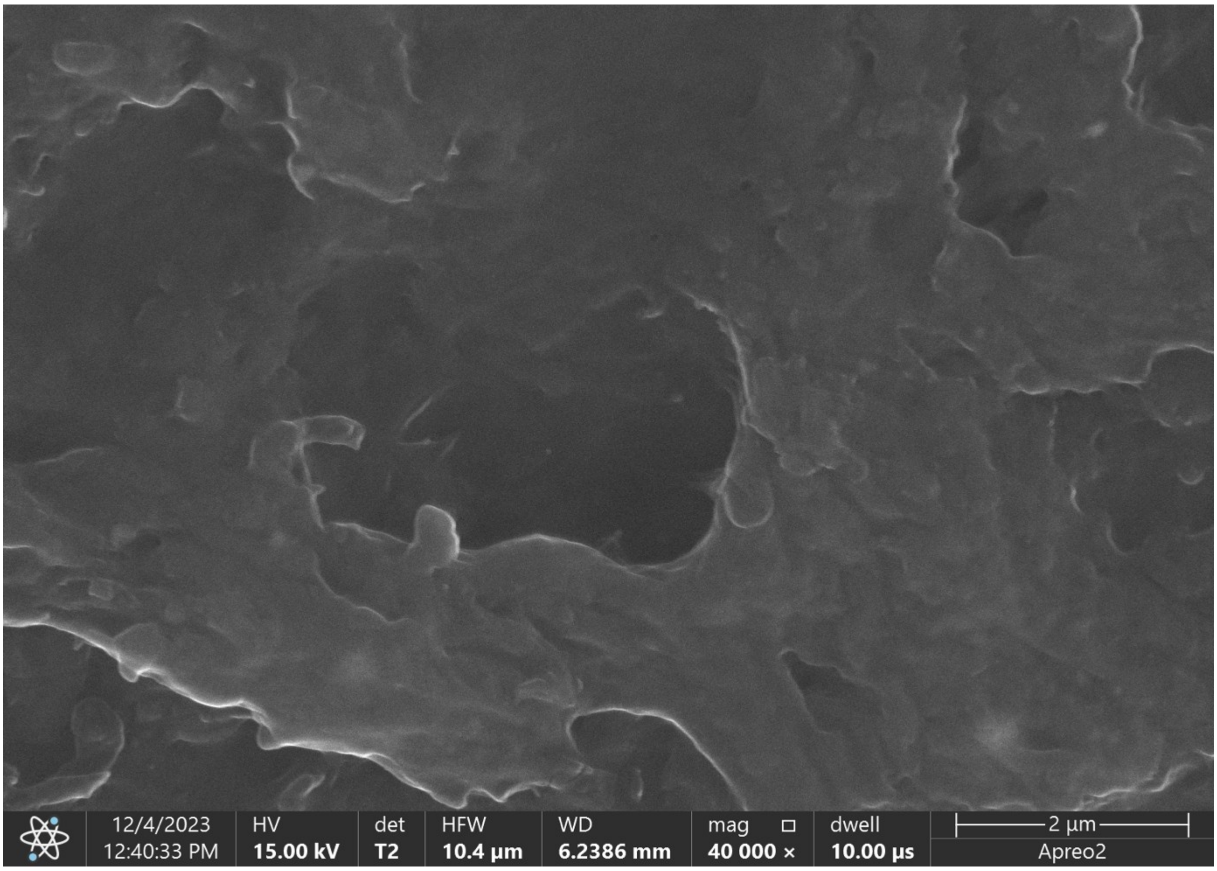

Figure S8. SEM graph of P3HT pellet cross-section (T2)

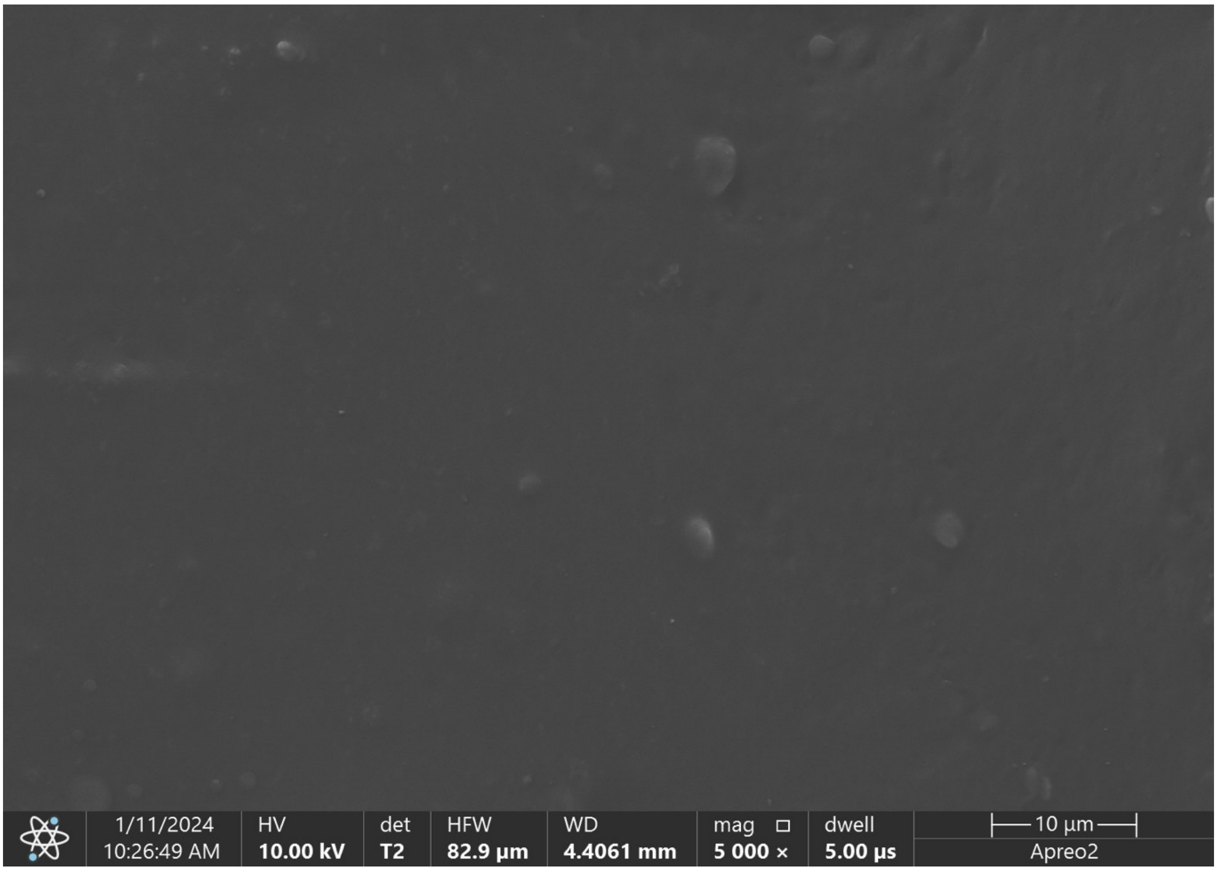

Figure S9. SEM graph of P3HT:DBSA 2:3 mass ratio pellet cross-section (T2)

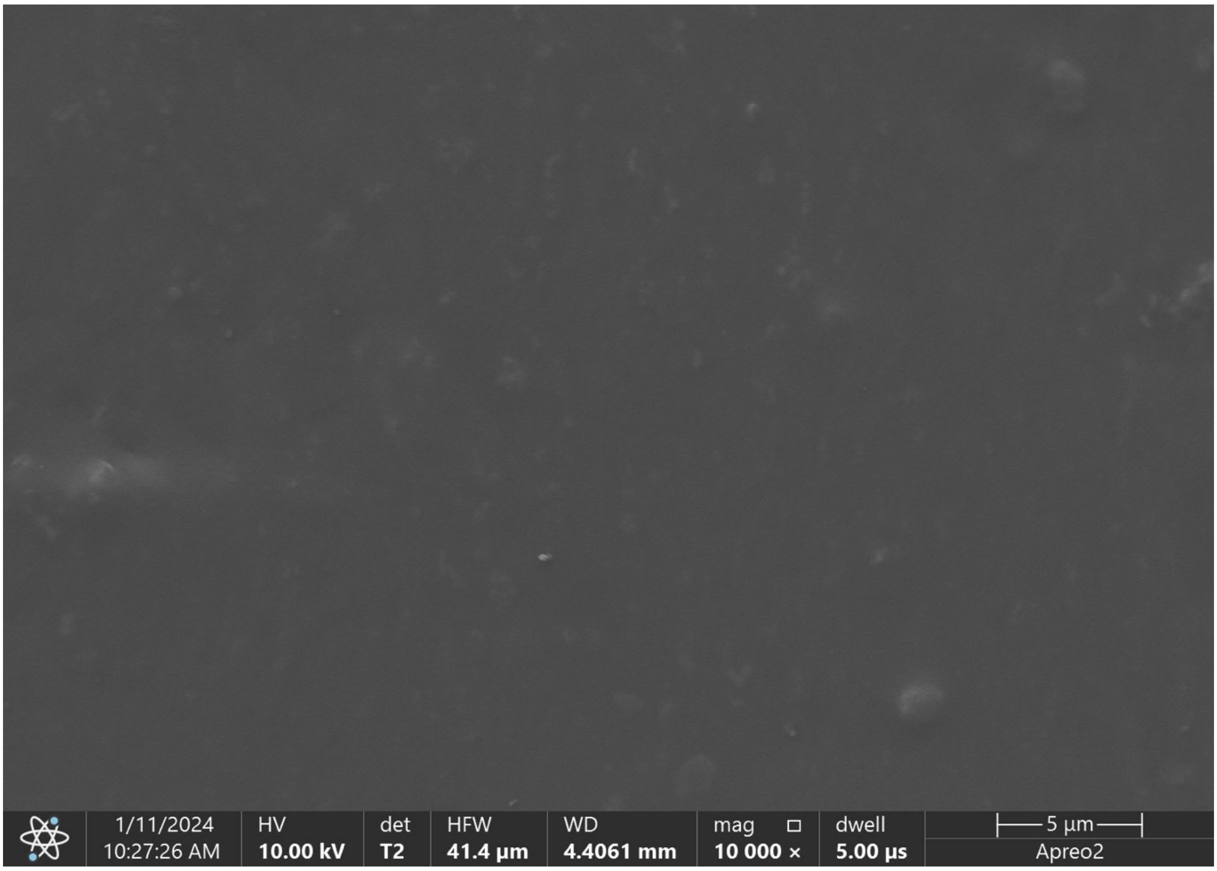

Figure S10. SEM graph of P3HT:DBSA 2:3 mass ratio pellet cross-section (T2)

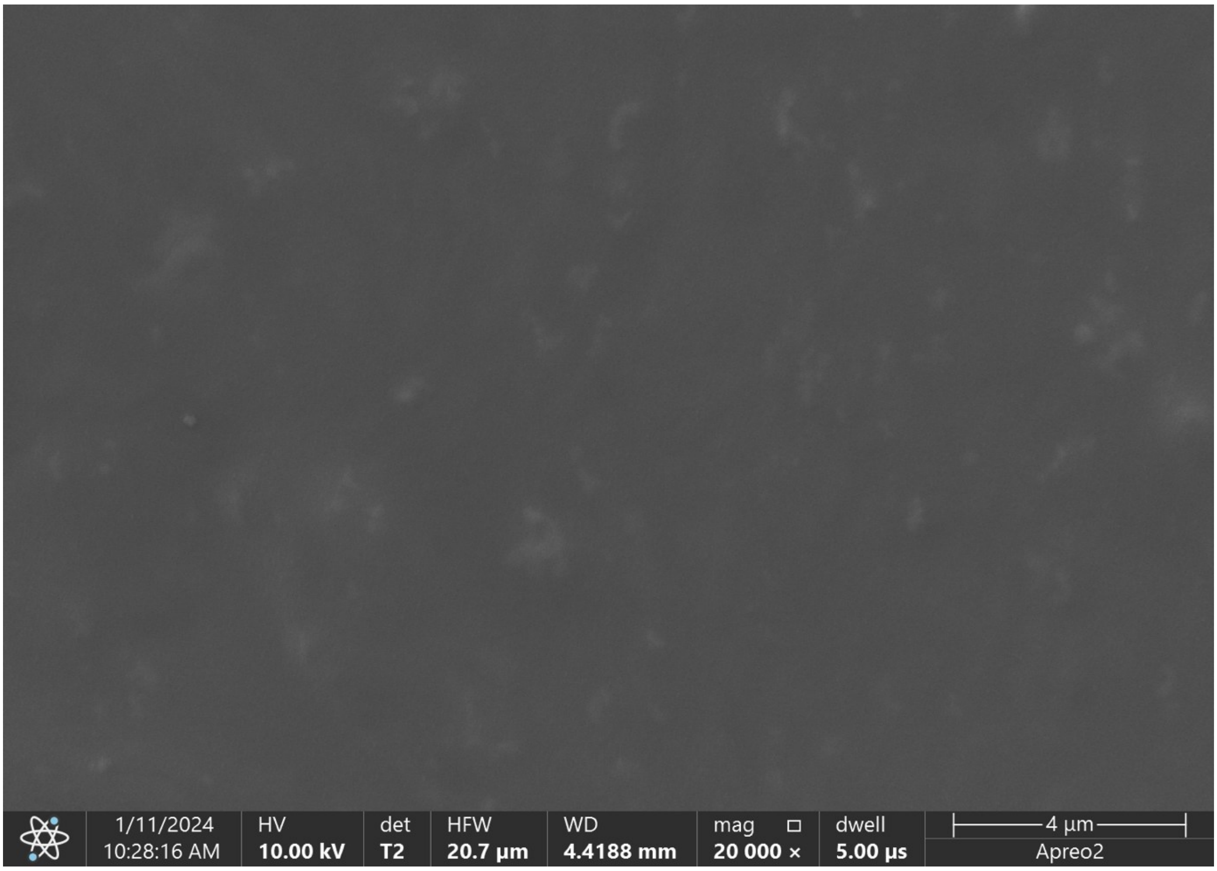

Figure S11. SEM graph of P3HT:DBSA 2:3 mass ratio pellet cross-section (T2)

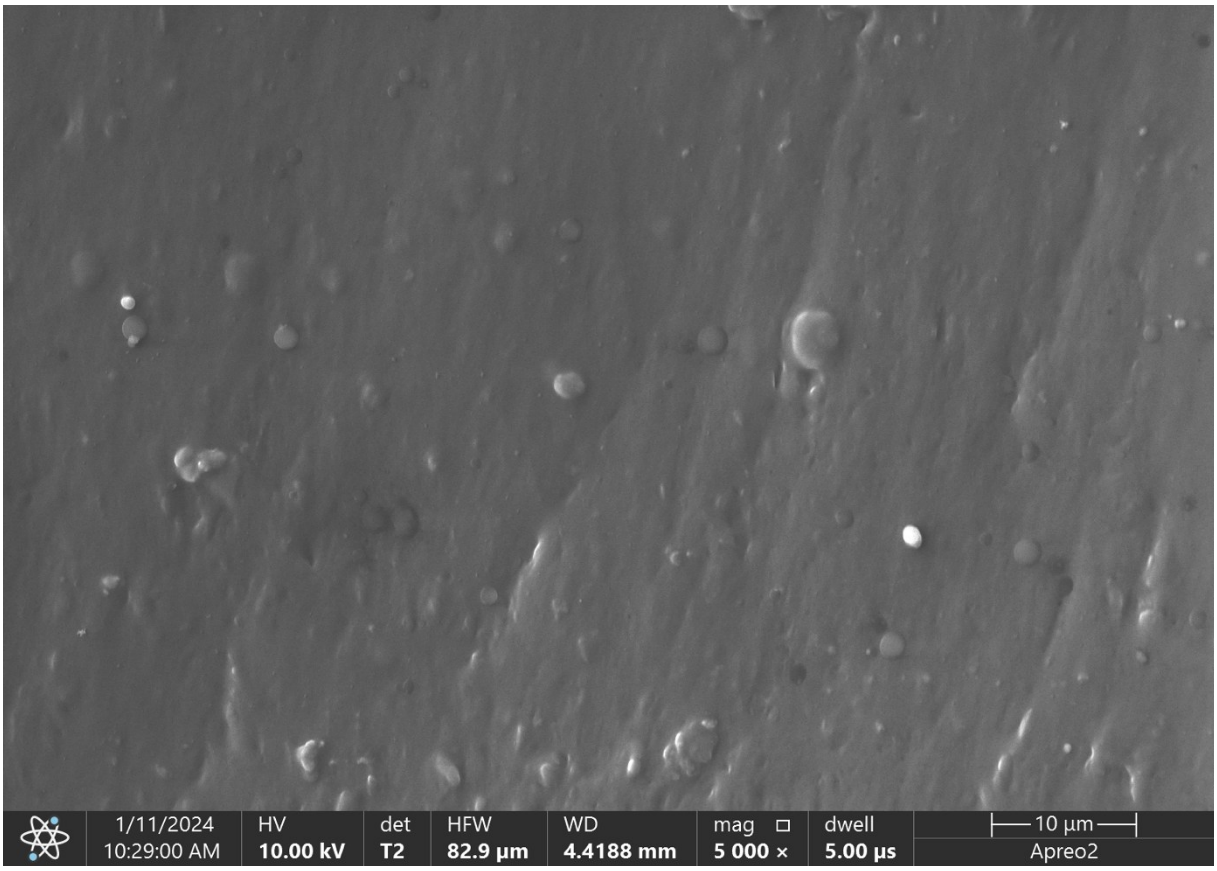

Figure S12. SEM graph of P3HT:DBSA 2:3 mass ratio pellet cross-section (T2)

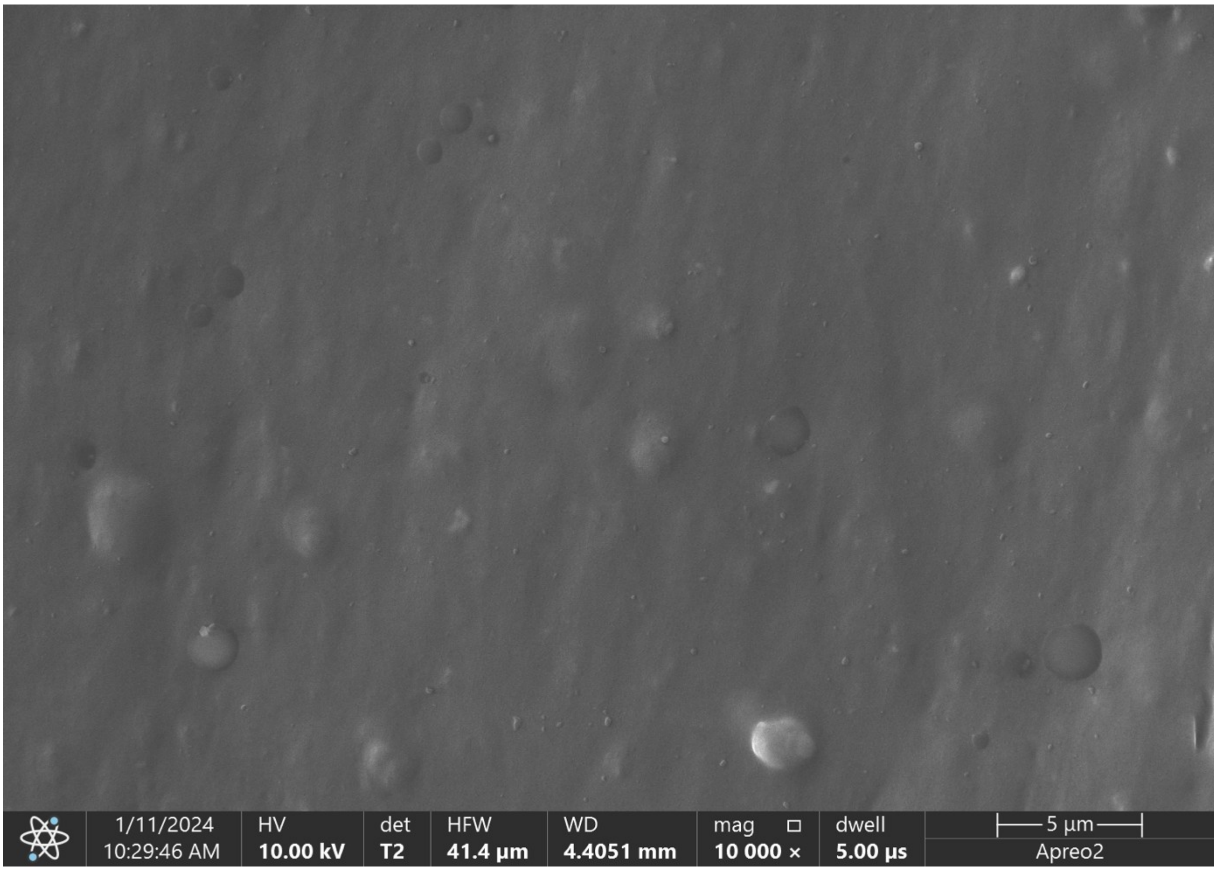

Figure S13. SEM graph of P3HT:DBSA 2:3 mass ratio pellet cross-section (T2)

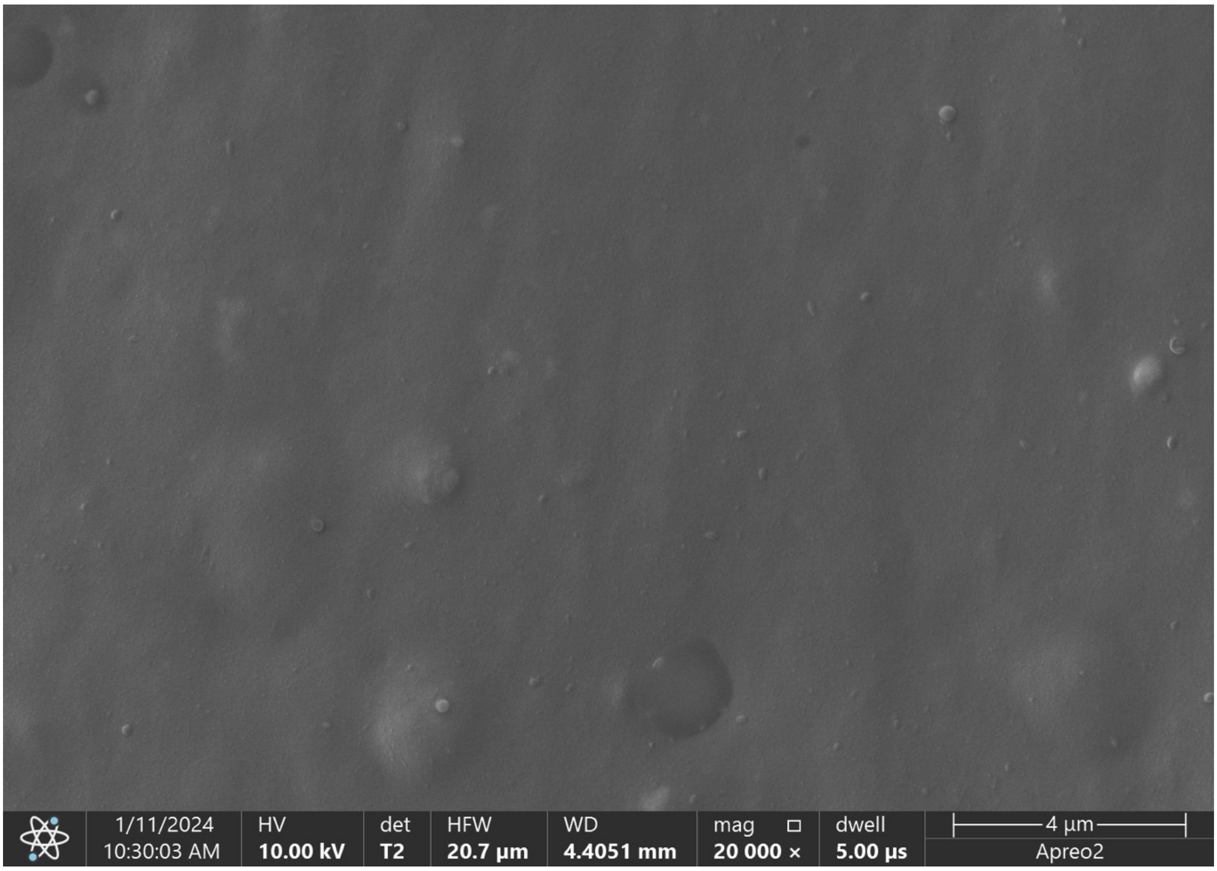

Figure S14. SEM graph of P3HT:DBSA 2:3 mass ratio pellet cross-section (T2)

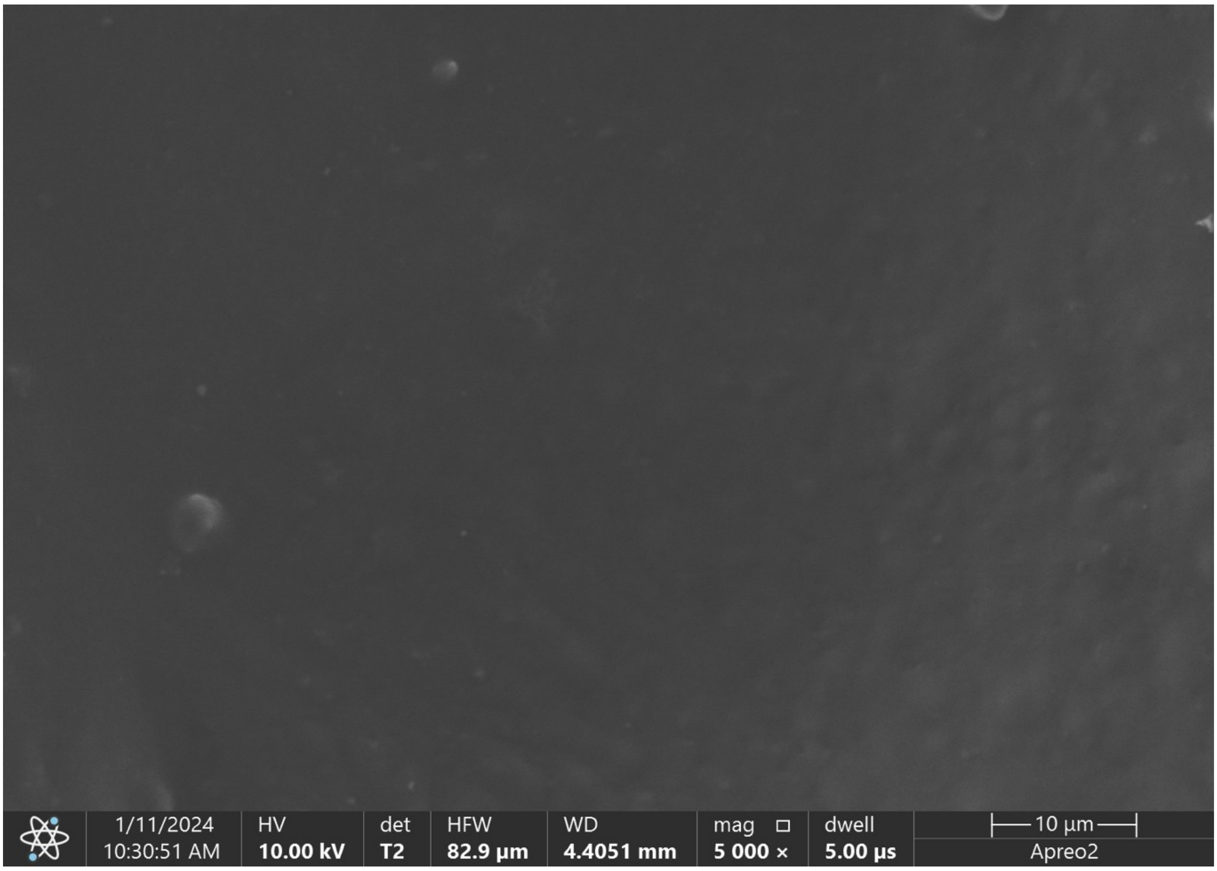

Figure S15. SEM graph of P3HT:DBSA 2:3 mass ratio pellet cross-section (T2)

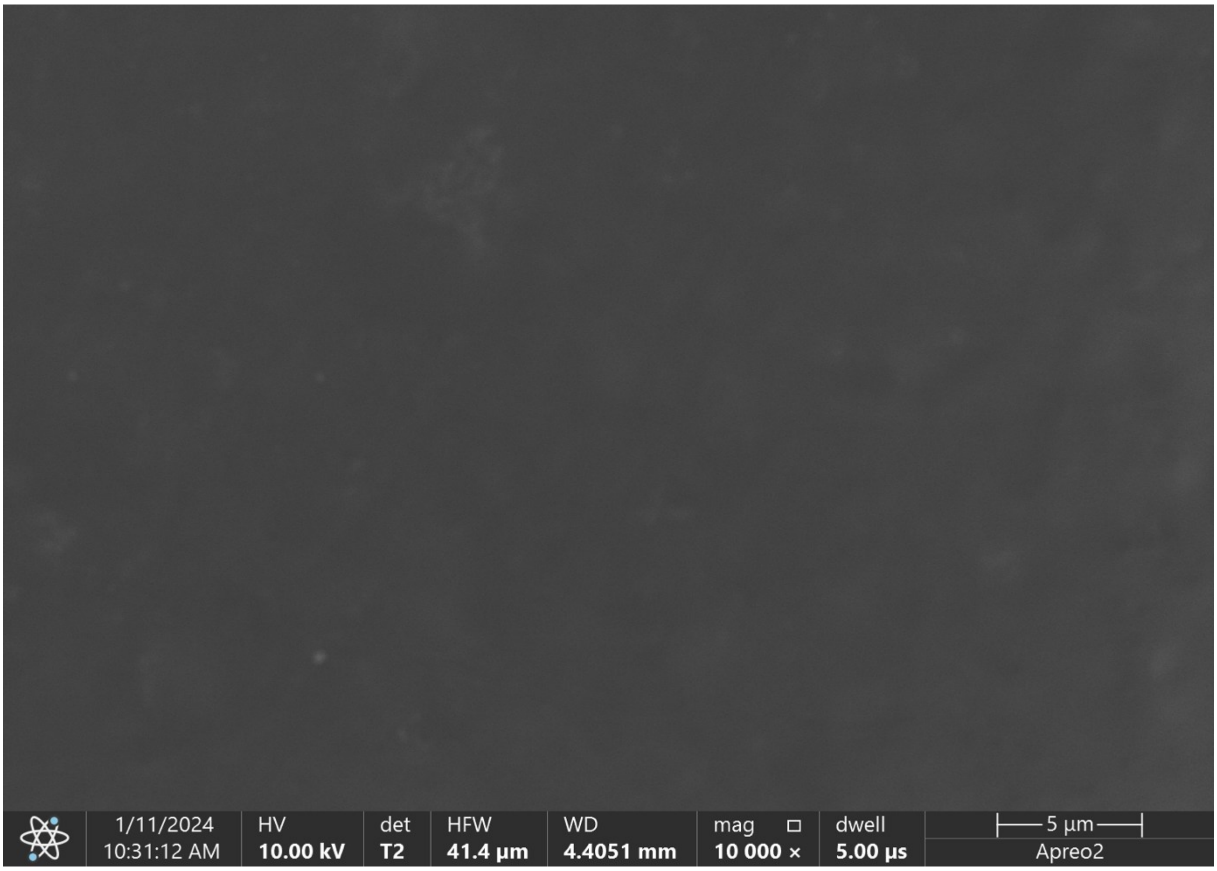

Figure S16. SEM graph of P3HT:DBSA 2:3 mass ratio pellet cross-section (T2)

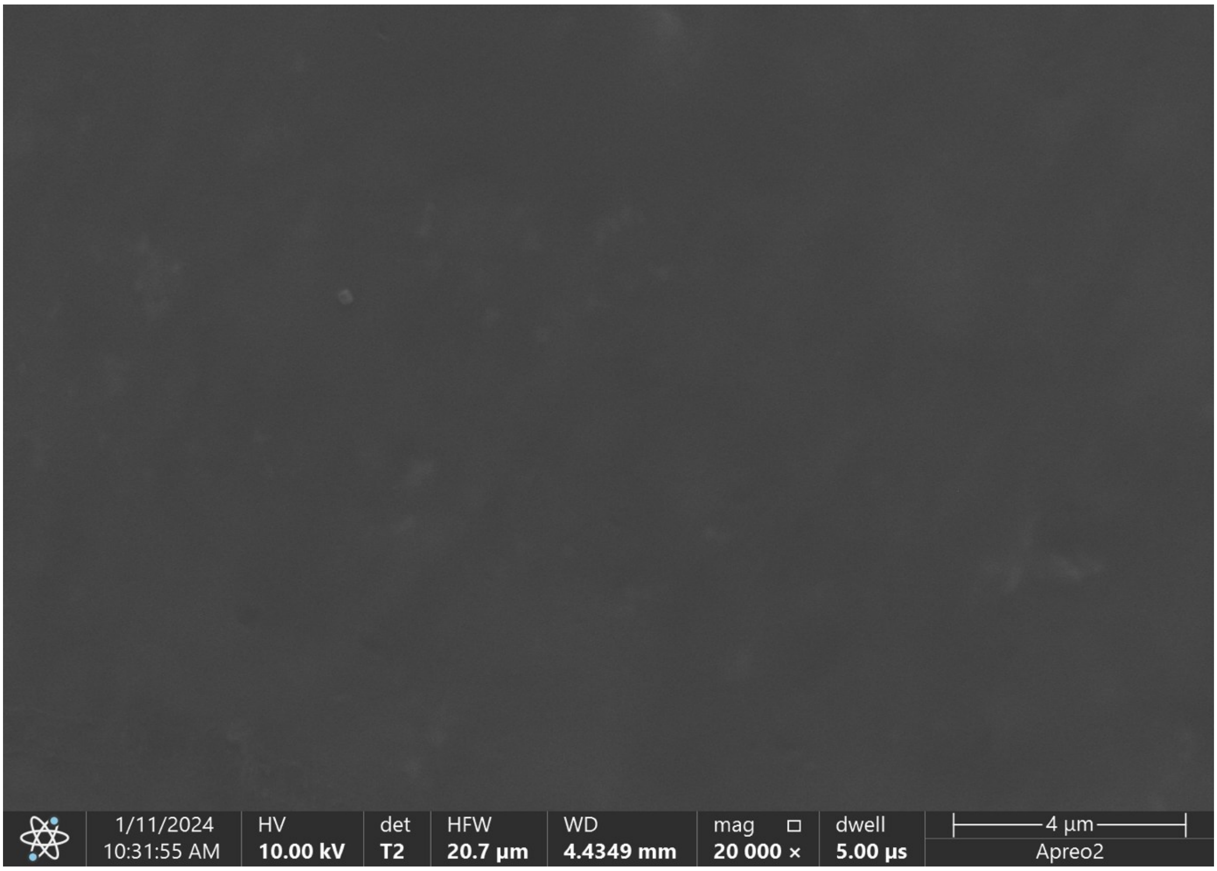

Figure S17. SEM graph of P3HT:DBSA 2:3 mass ratio pellet cross-section (T2)

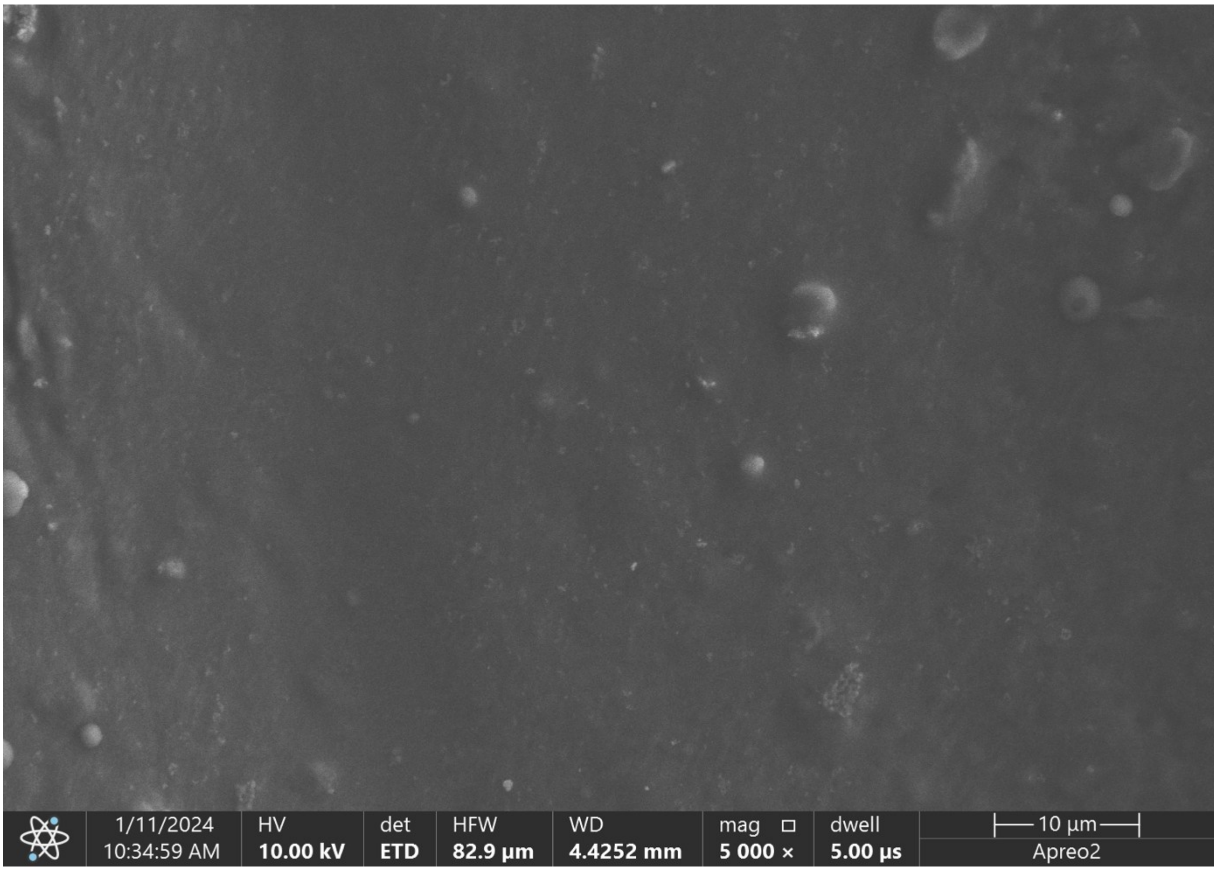

Figure S18. SEM graph of P3HT:DBSA 2:3 mass ratio pellet cross-section (ETD)

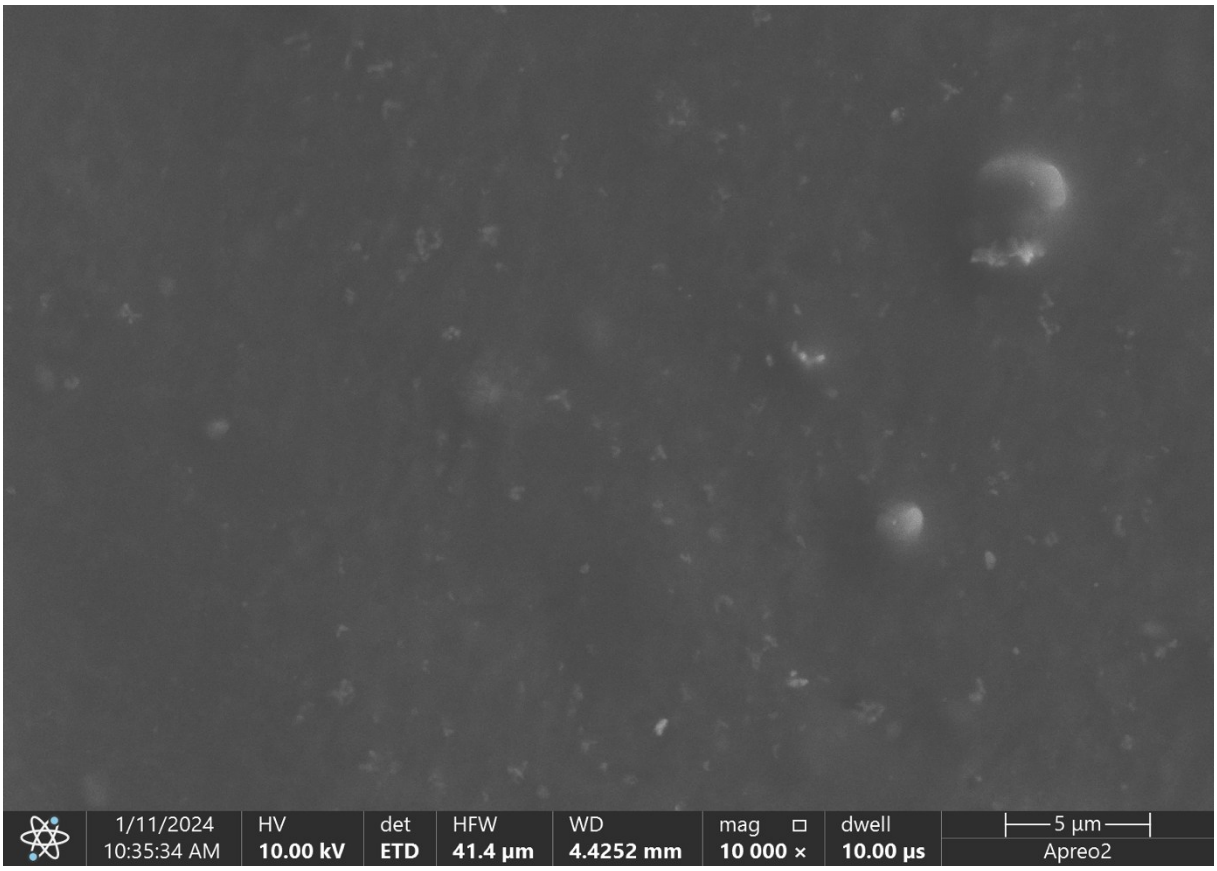

Figure S19. SEM graph of P3HT:DBSA 2:3 mass ratio pellet cross-section (ETD)

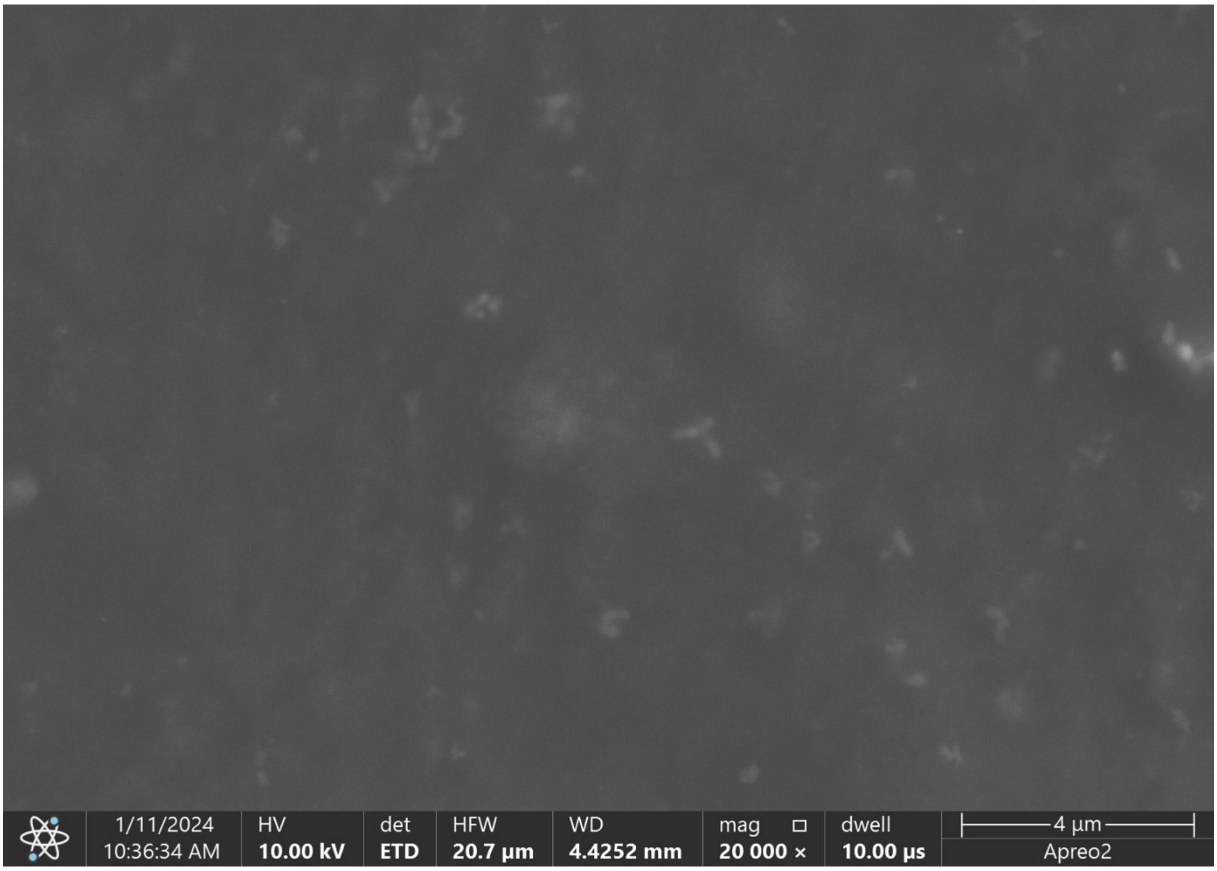

Figure S20. SEM graph of P3HT:DBSA 2:3 mass ratio pellet cross-section (ETD)

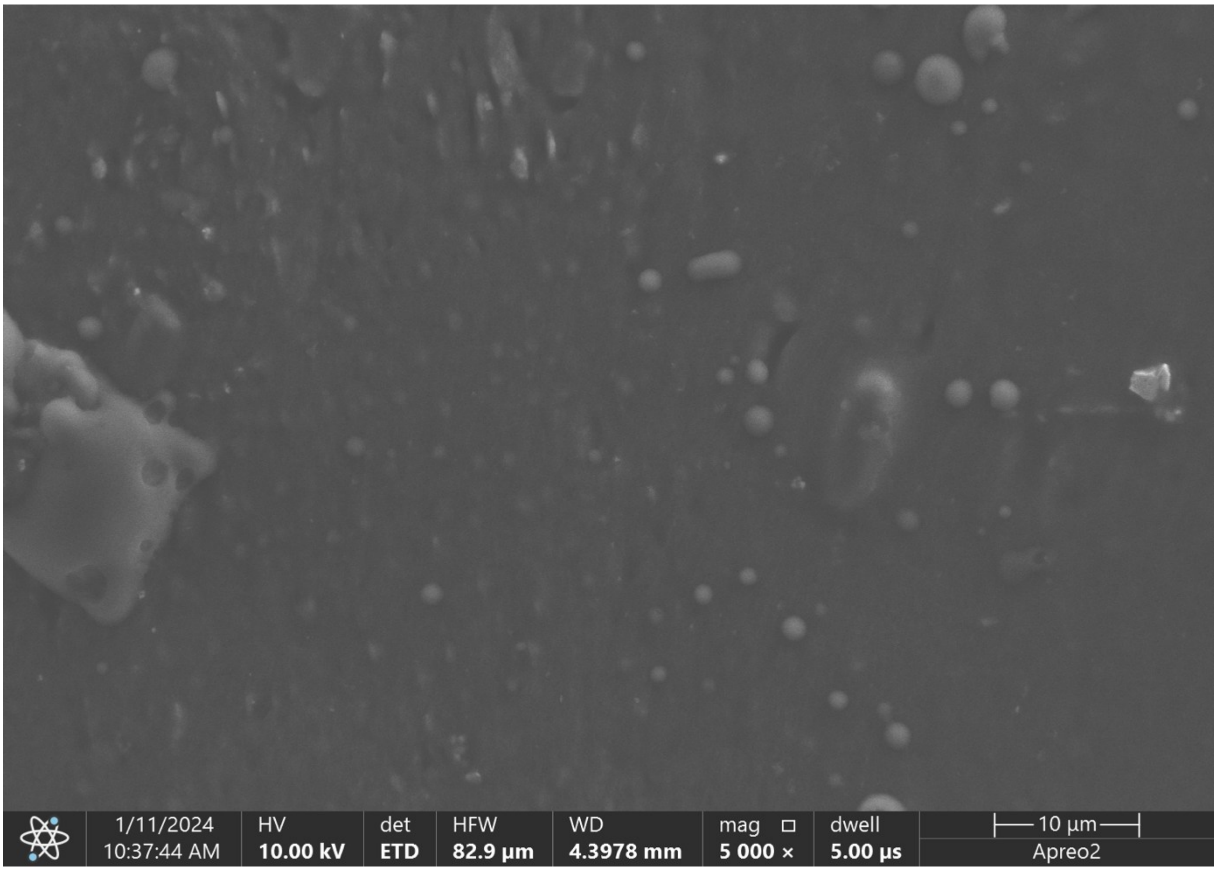

Figure S21. SEM graph of P3HT:DBSA 2:3 mass ratio pellet cross-section (ETD)

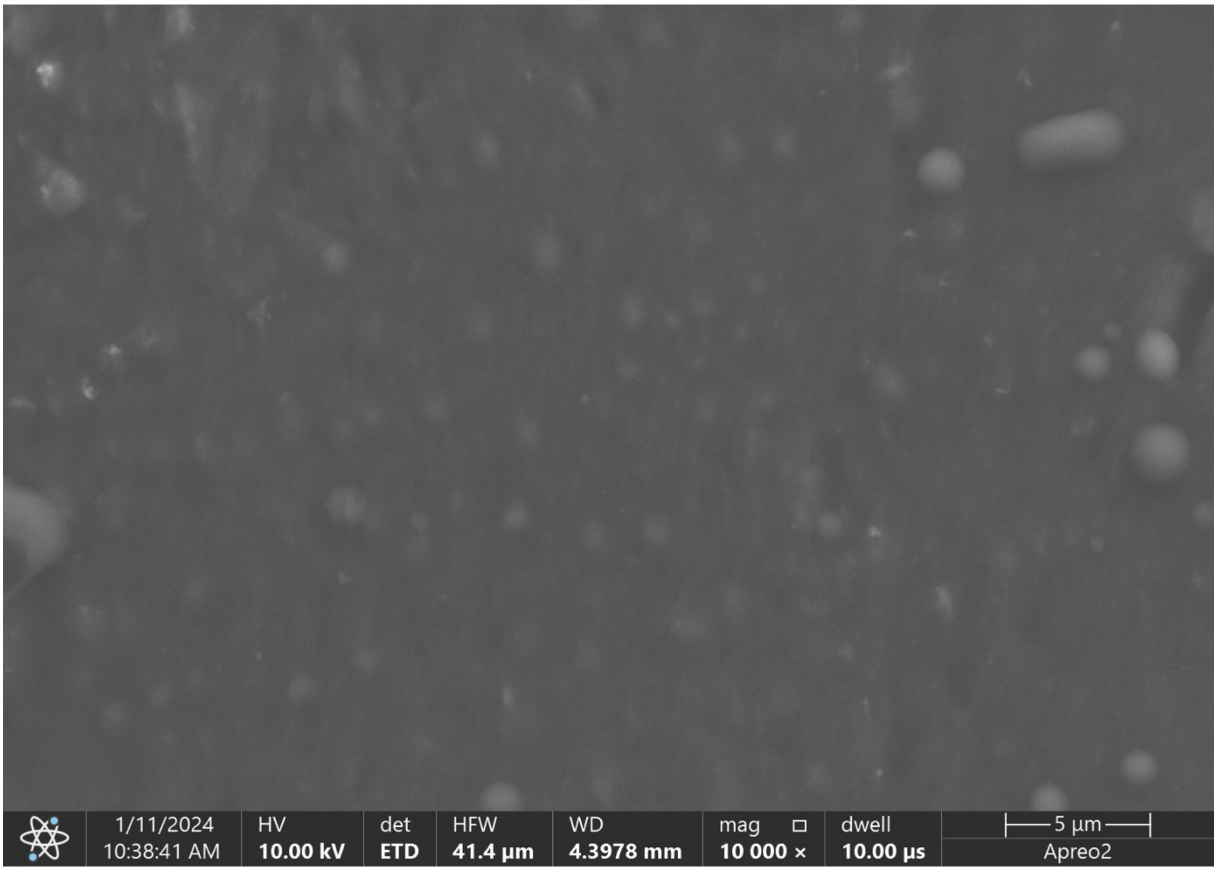

Figure S22. SEM graph of P3HT:DBSA 2:3 mass ratio pellet cross-section (ETD)

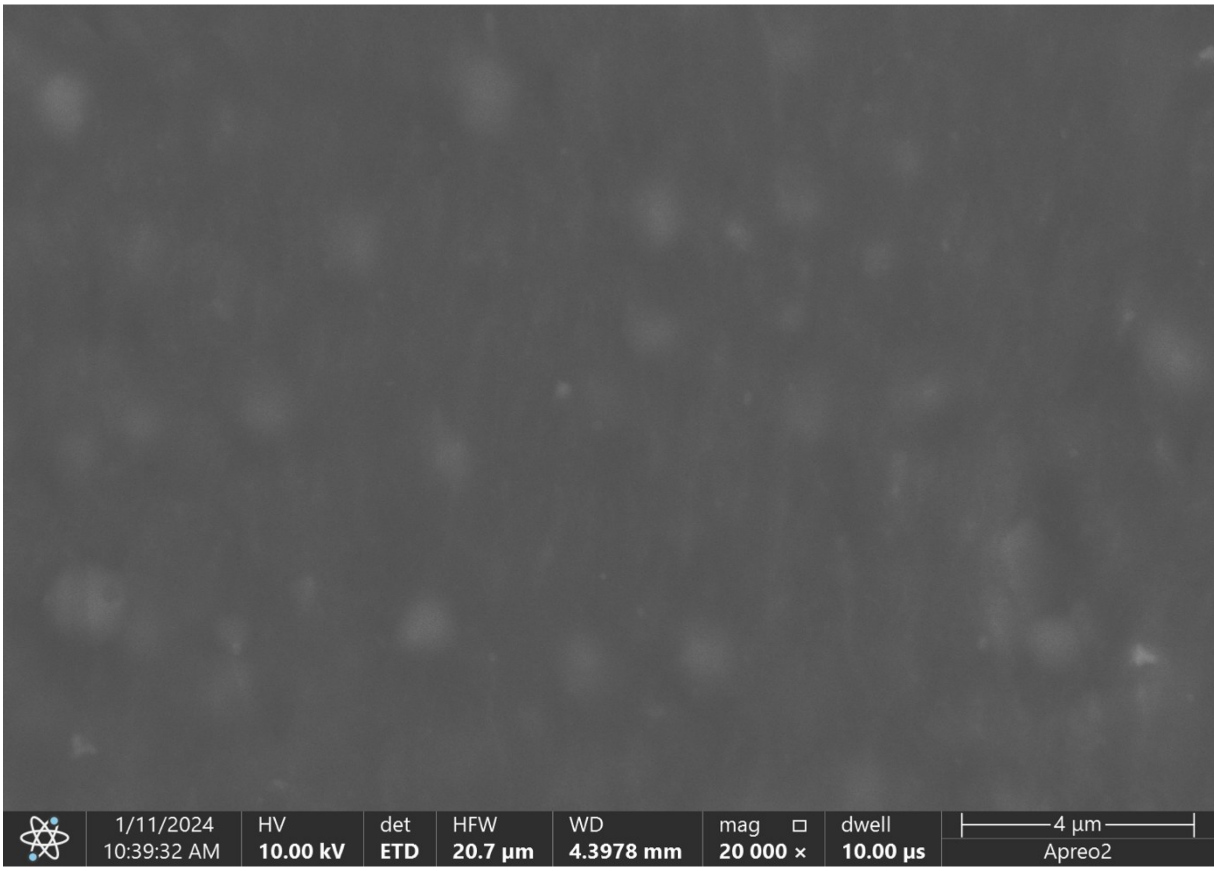

Figure S23. SEM graph of P3HT:DBSA 2:3 mass ratio pellet cross-section (ETD)

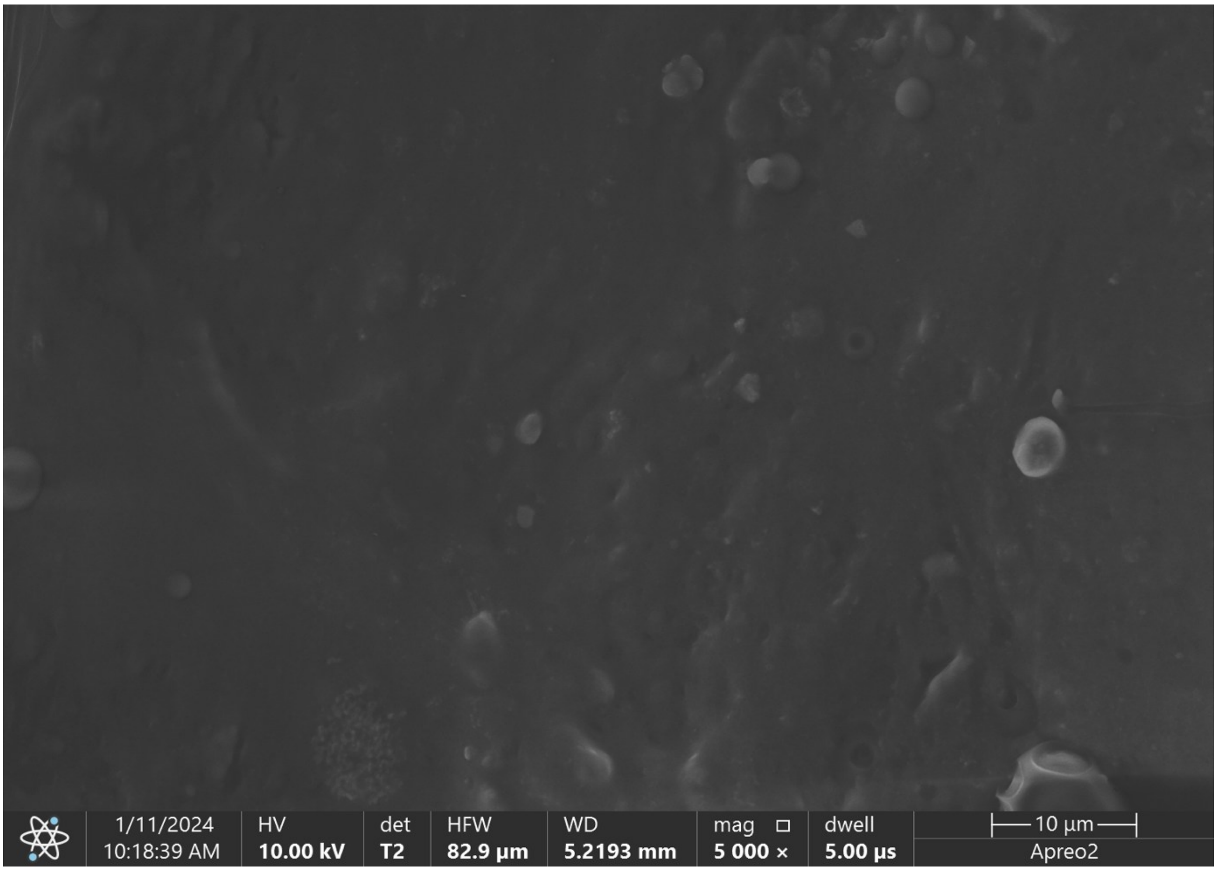

Figure S24. SEM graph of P3HT:DBSA 1:10 mass ratio pellet cross-section (T2)

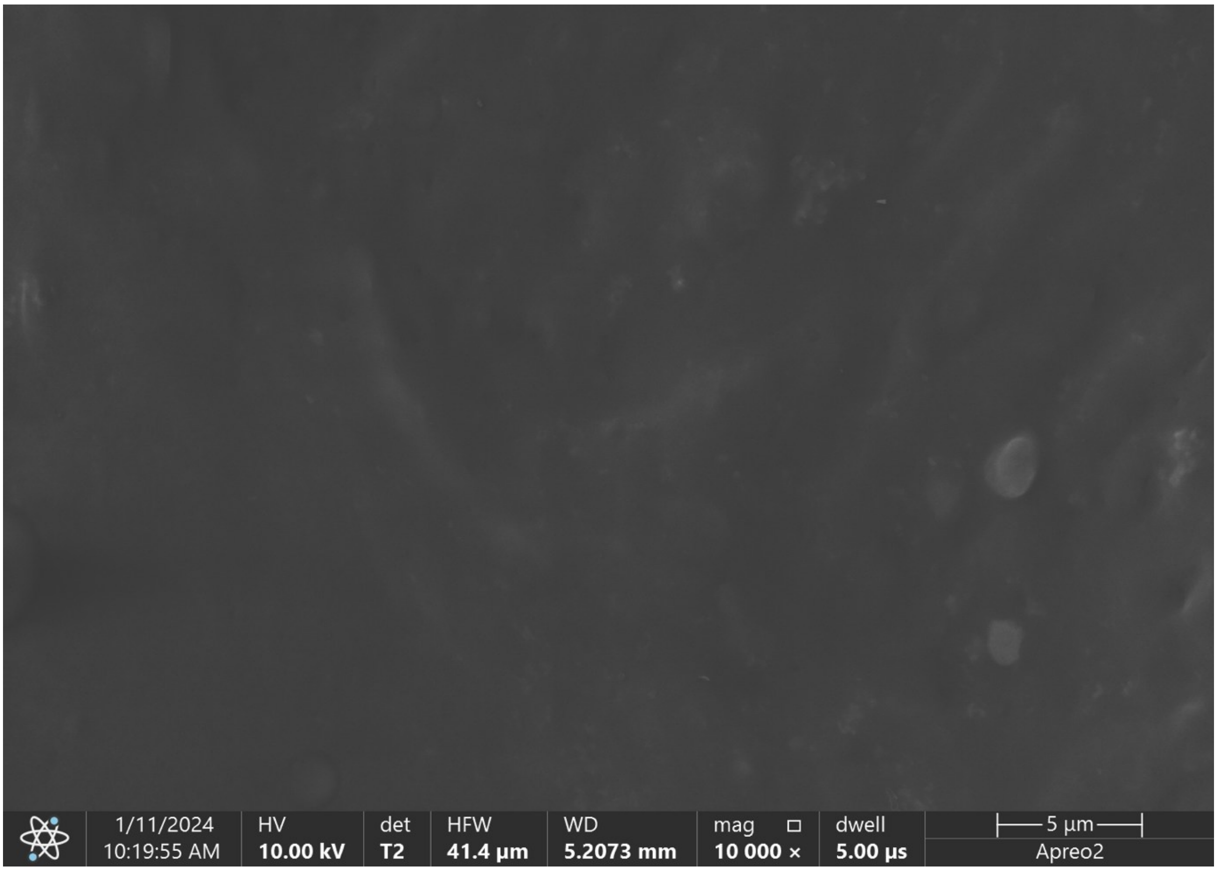

Figure S25. SEM graph of P3HT:DBSA 1:10 mass ratio pellet cross-section (T2)

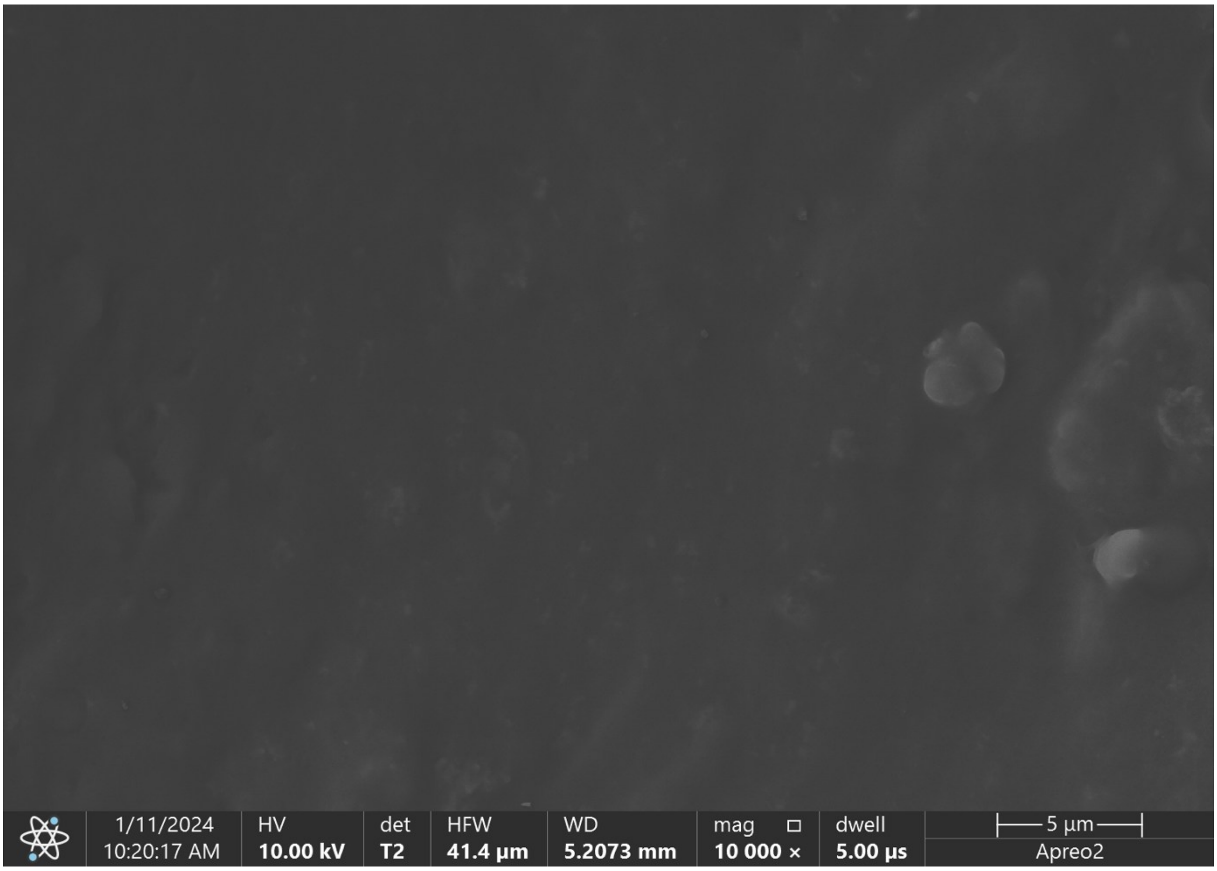

Figure S26. SEM graph of P3HT:DBSA 1:10 mass ratio pellet cross-section (T2)

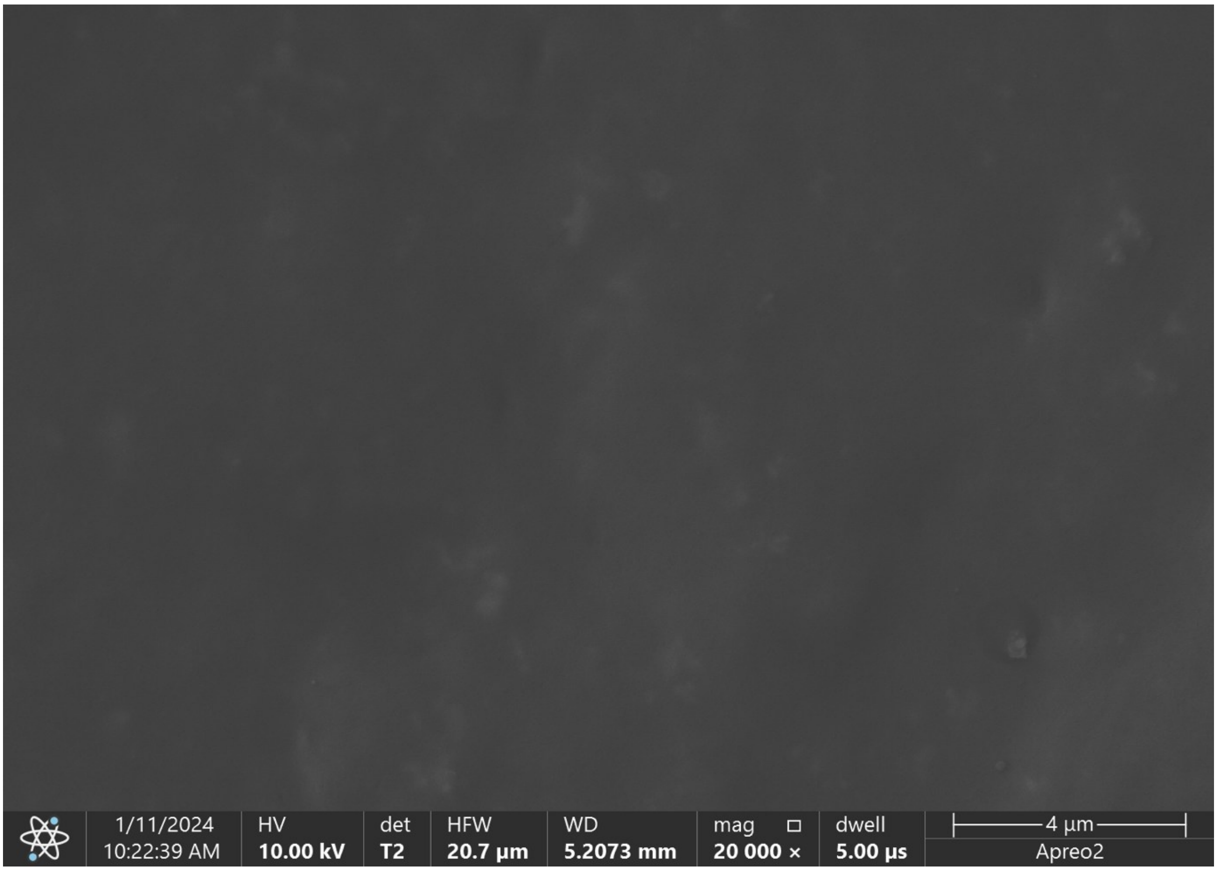

Figure S27. SEM graph of P3HT:DBSA 1:10 mass ratio pellet cross-section (T2)

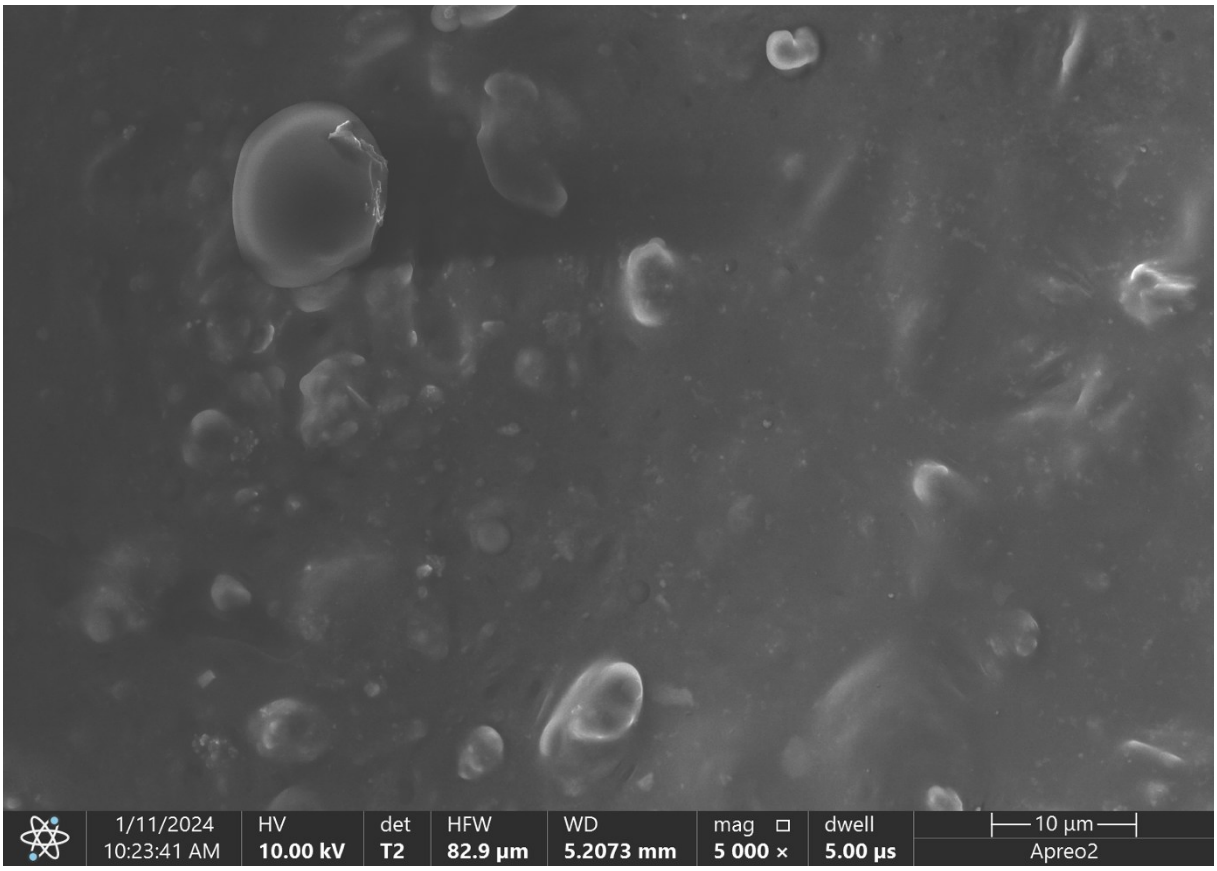

Figure S28. SEM graph of P3HT:DBSA 1:10 mass ratio pellet cross-section (T2)

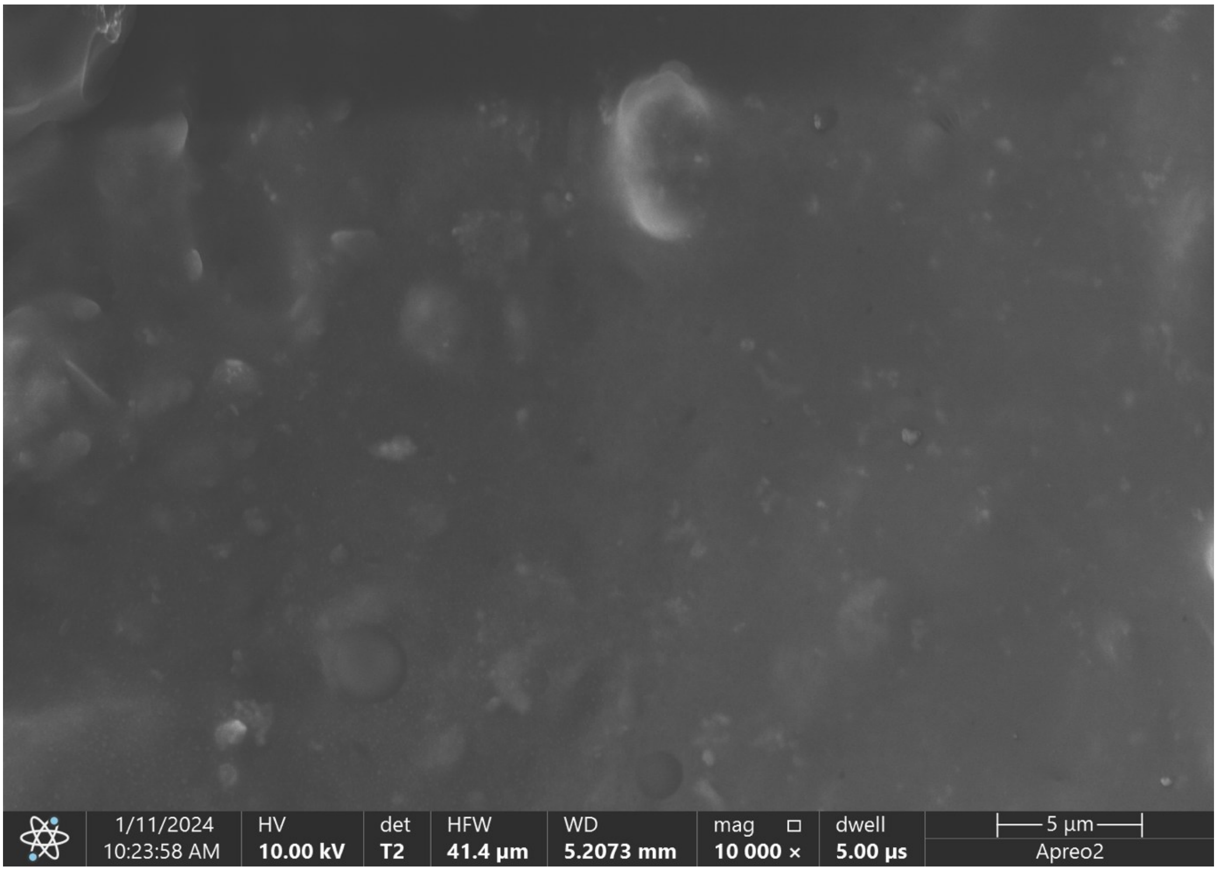

Figure S29. SEM graph of P3HT:DBSA 1:10 mass ratio pellet cross-section (T2)

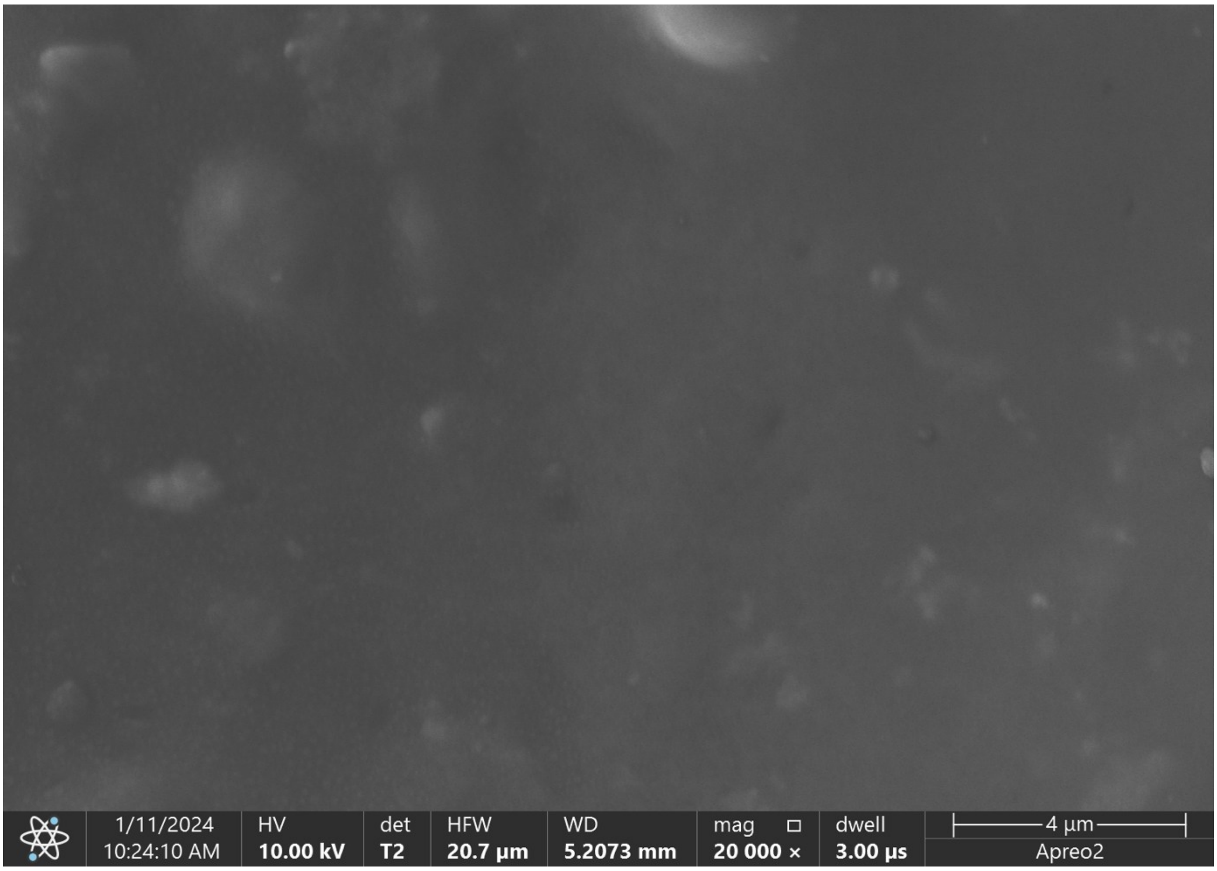

Figure S30. SEM graph of P3HT:DBSA 1:10 mass ratio pellet cross-section (T2)

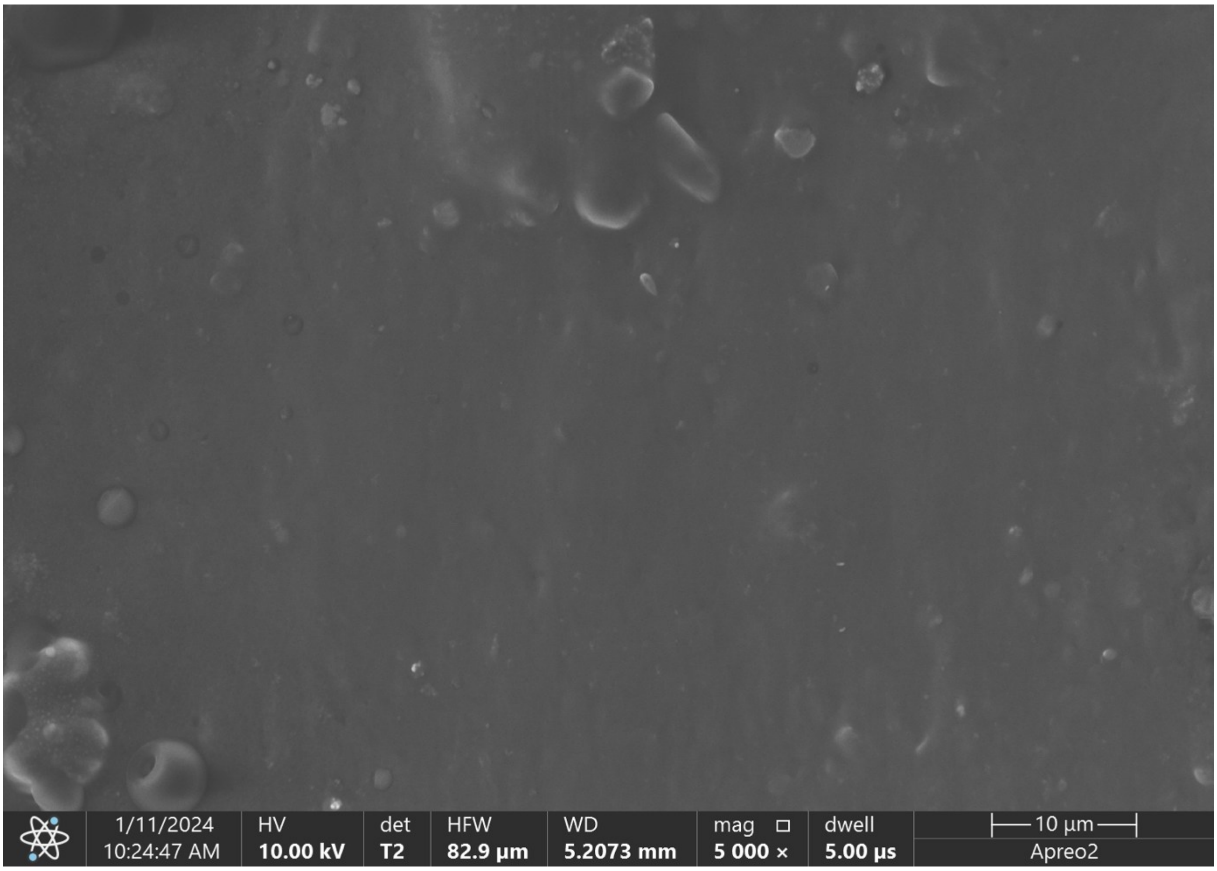

Figure S31. SEM graph of P3HT:DBSA 1:10 mass ratio pellet cross-section (T2)

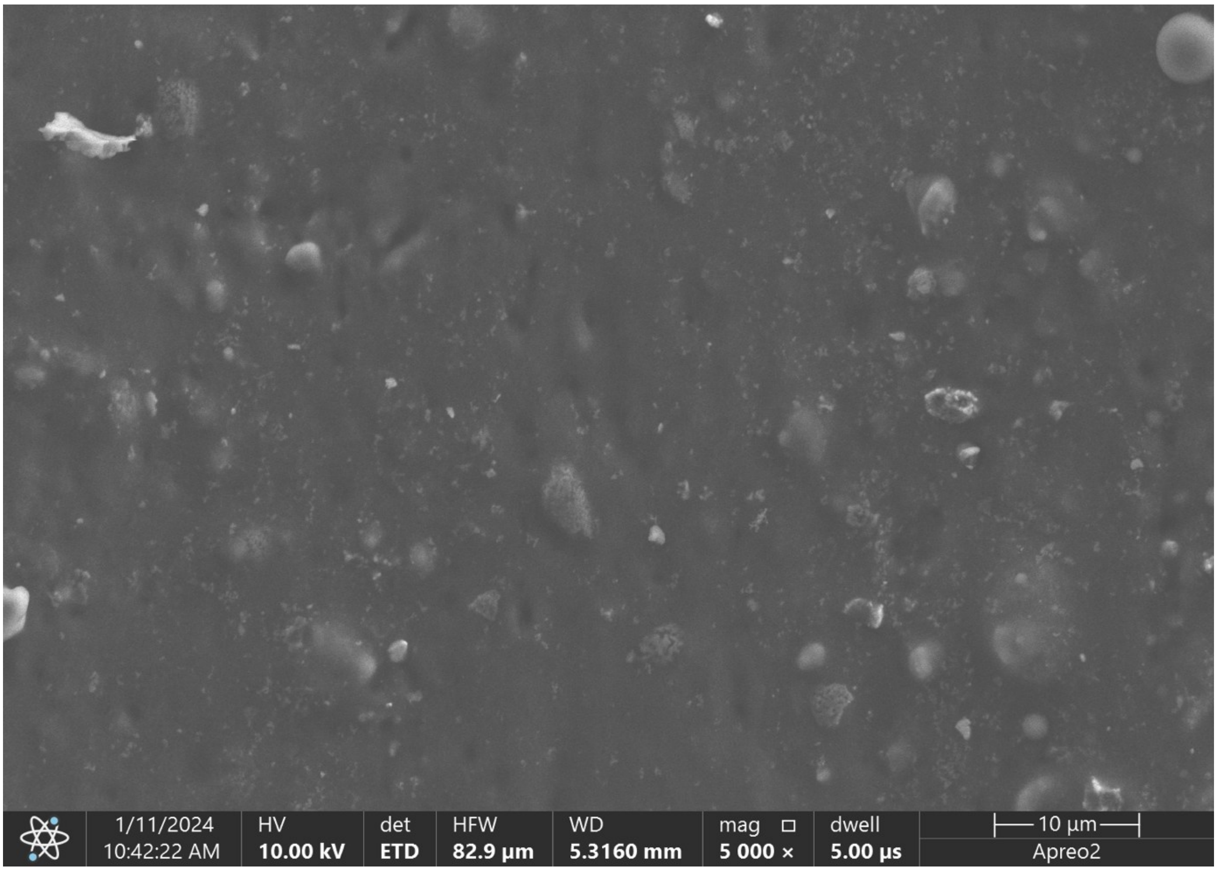

Figure S32. SEM graph of P3HT:DBSA 1:10 mass ratio pellet cross-section (ETD)

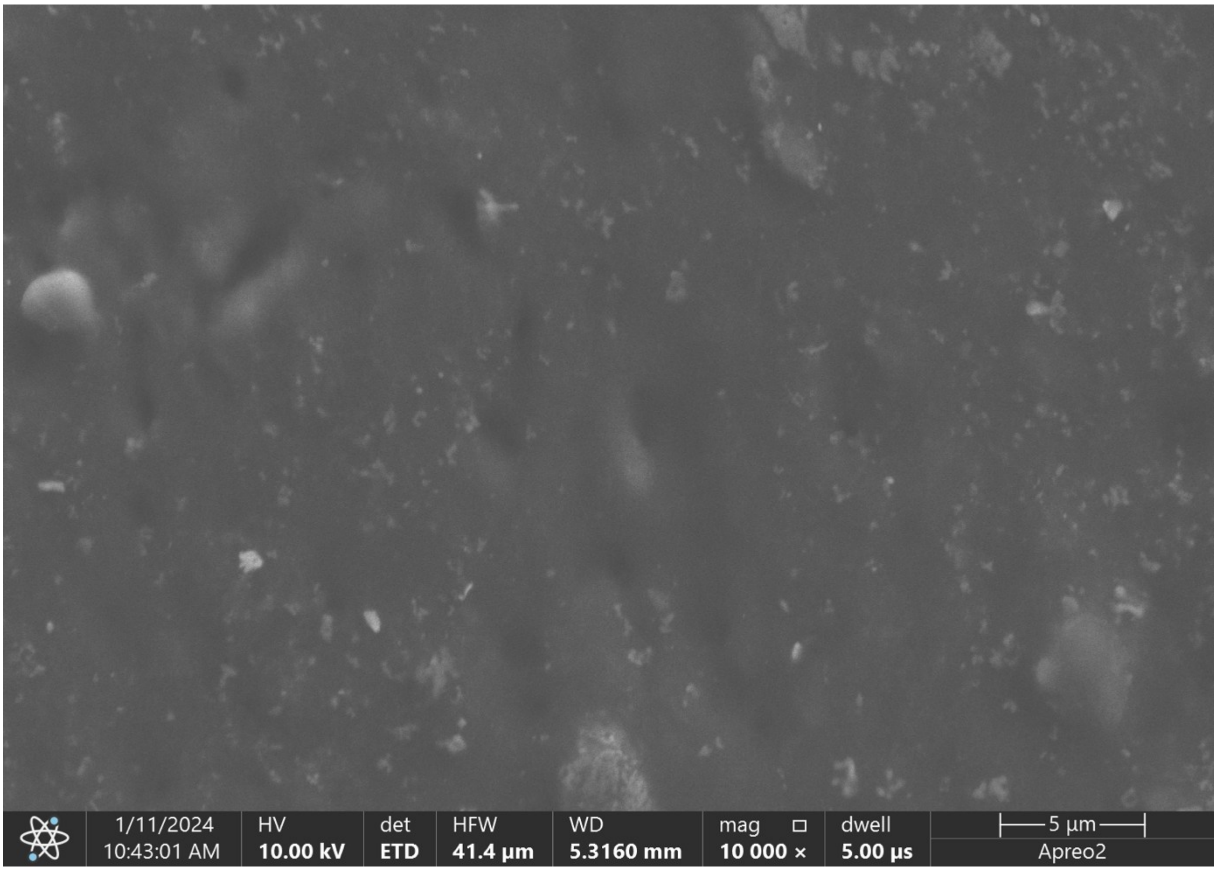

Figure S33. SEM graph of P3HT:DBSA 1:10 mass ratio pellet cross-section (ETD)

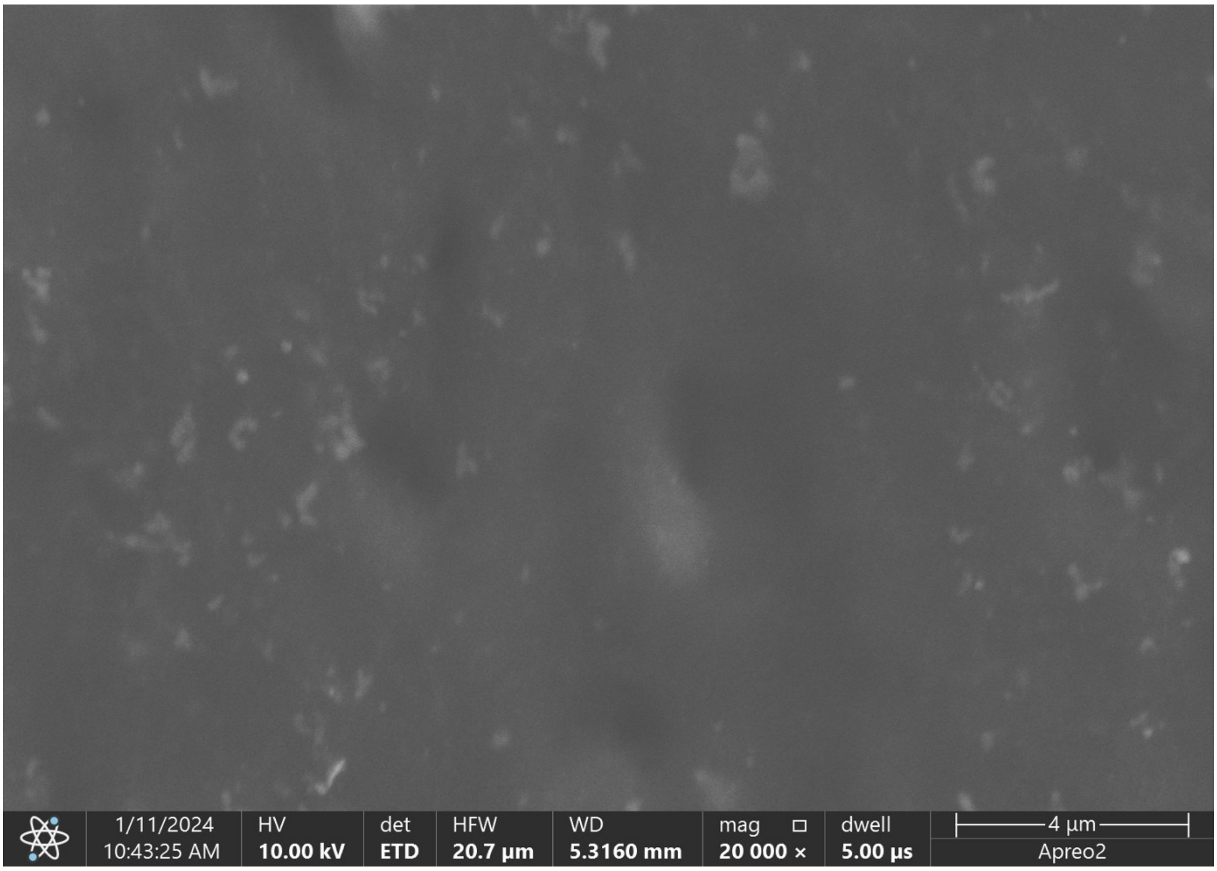

Figure S34. SEM graph of P3HT:DBSA 1:10 mass ratio pellet cross-section (ETD)

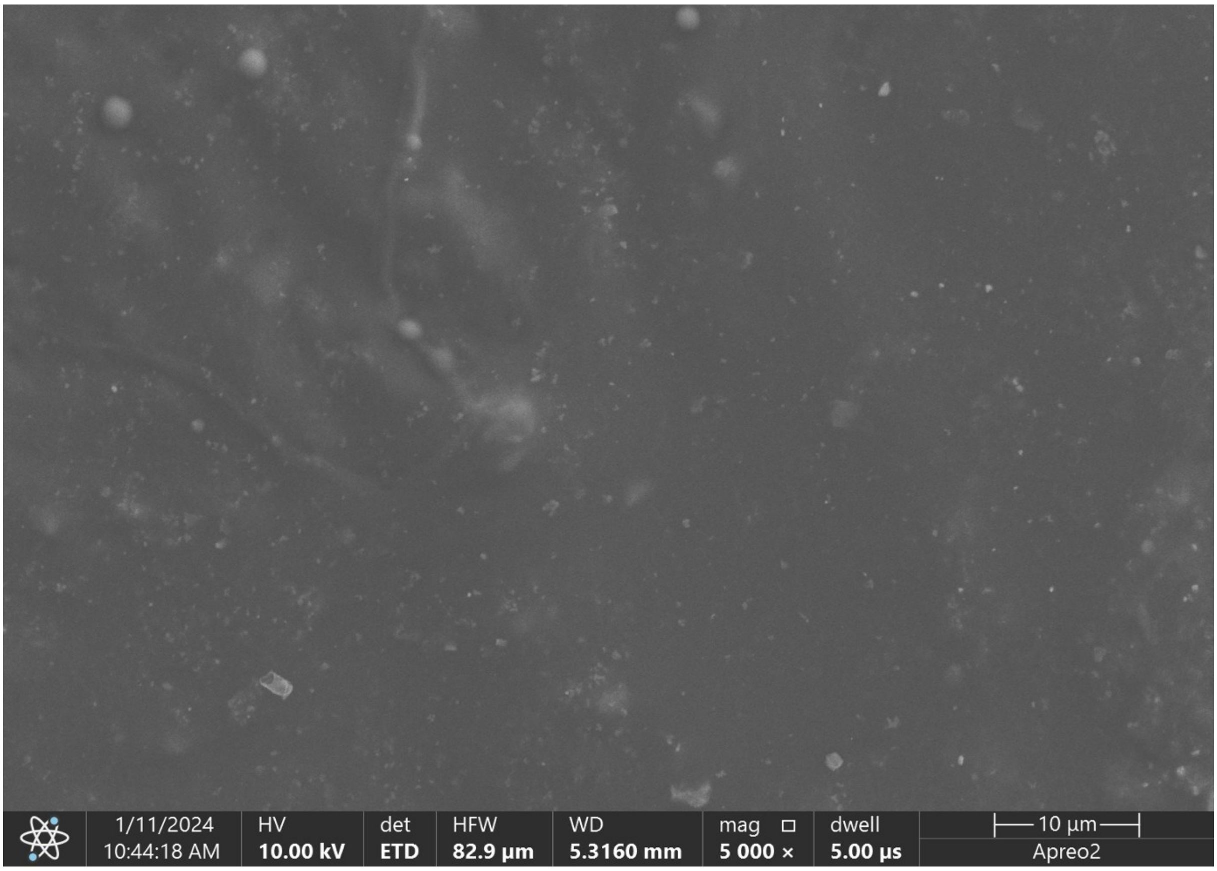

Figure S35. SEM graph of P3HT:DBSA 1:10 mass ratio pellet cross-section (ETD)

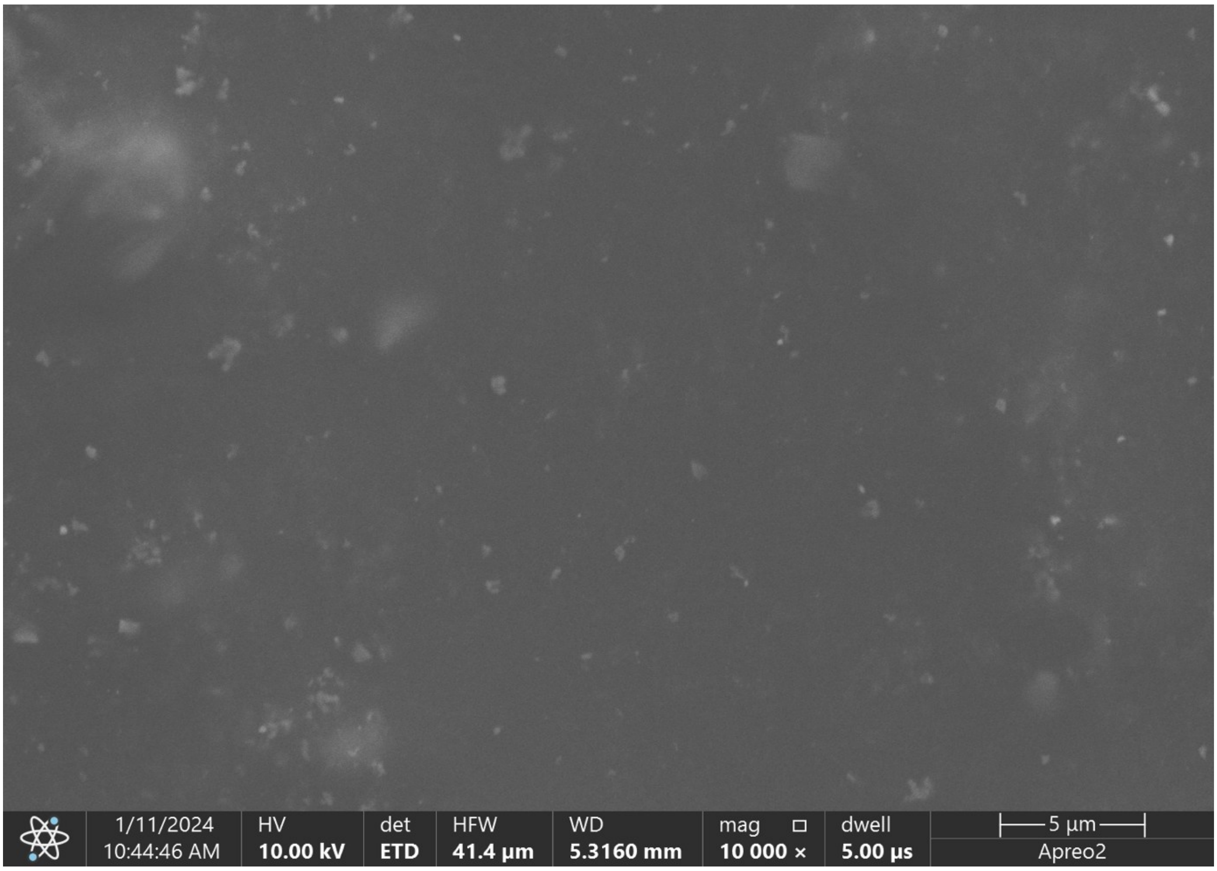

Figure S36. SEM graph of P3HT:DBSA 1:10 mass ratio pellet cross-section (ETD)

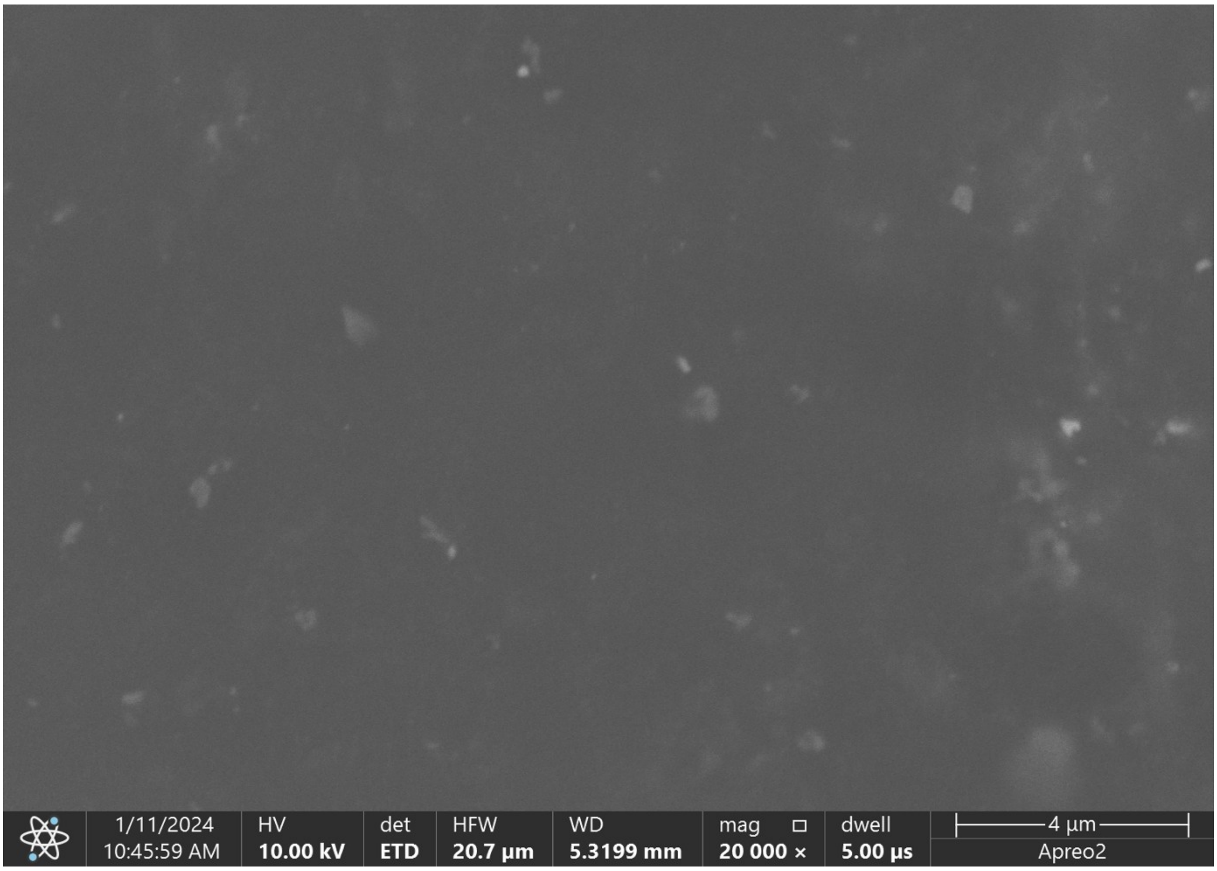

Figure S37. SEM graph of P3HT:DBSA 1:10 mass ratio pellet cross-section (ETD)

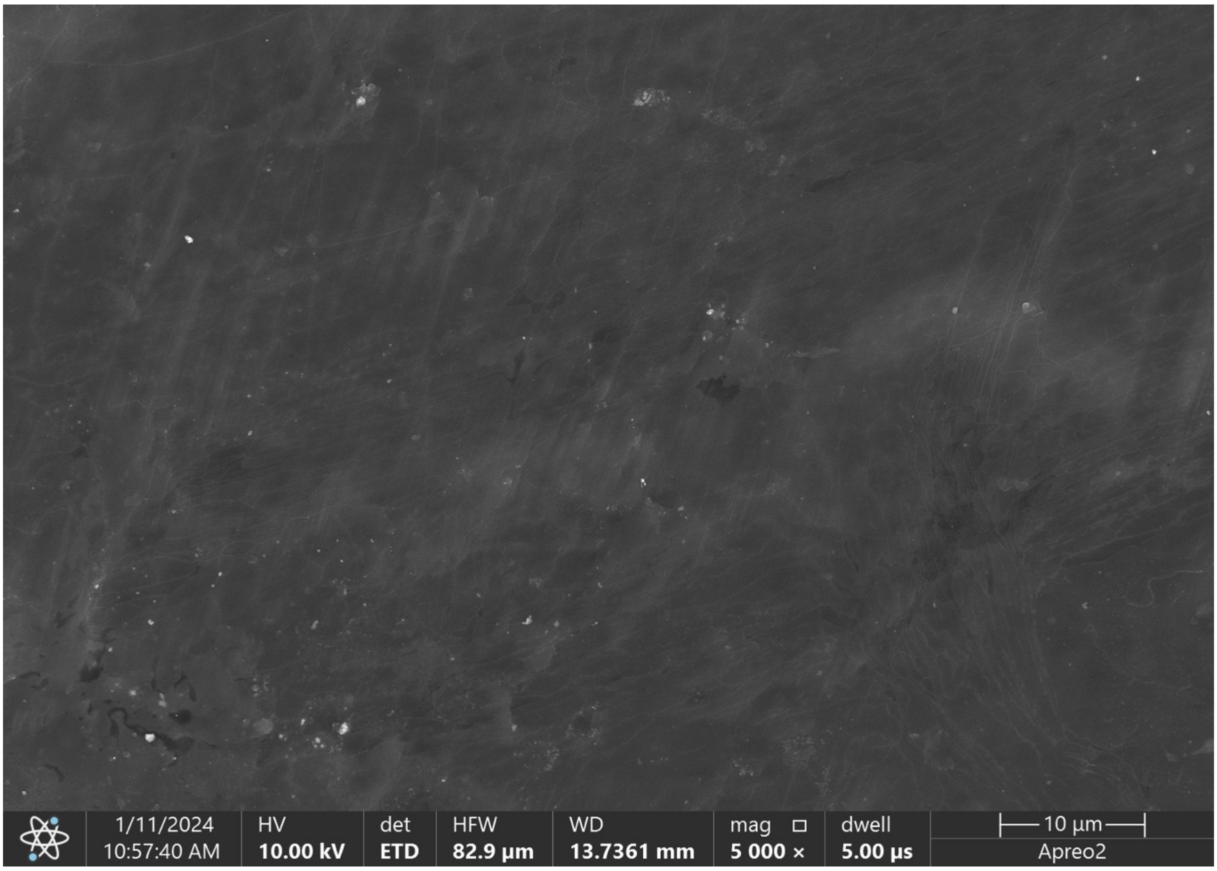

Figure S38. SEM graph of P3HT:DBSA 10:1 mass ratio pellet cross-section (ETD)

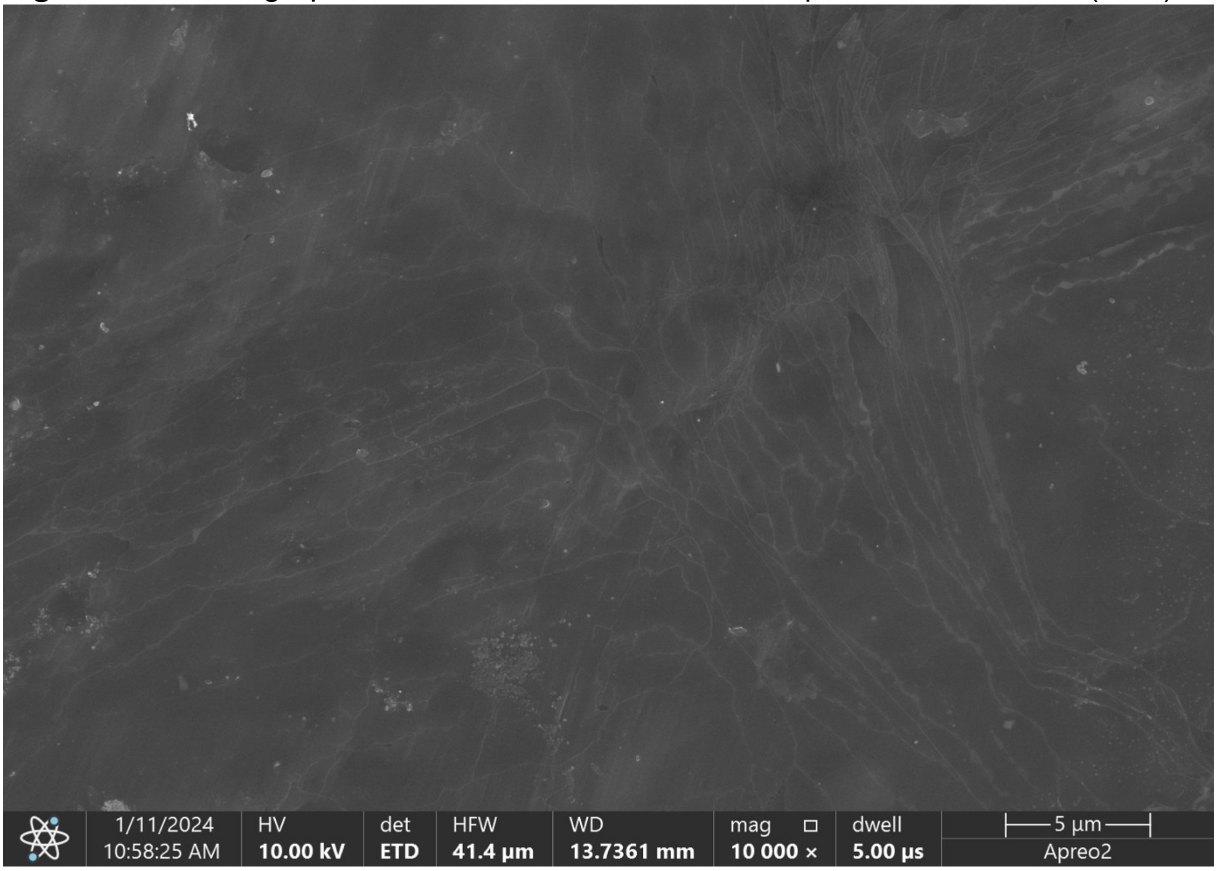

Figure S39. SEM graph of P3HT:DBSA 10:1 mass ratio pellet cross-section (ETD)

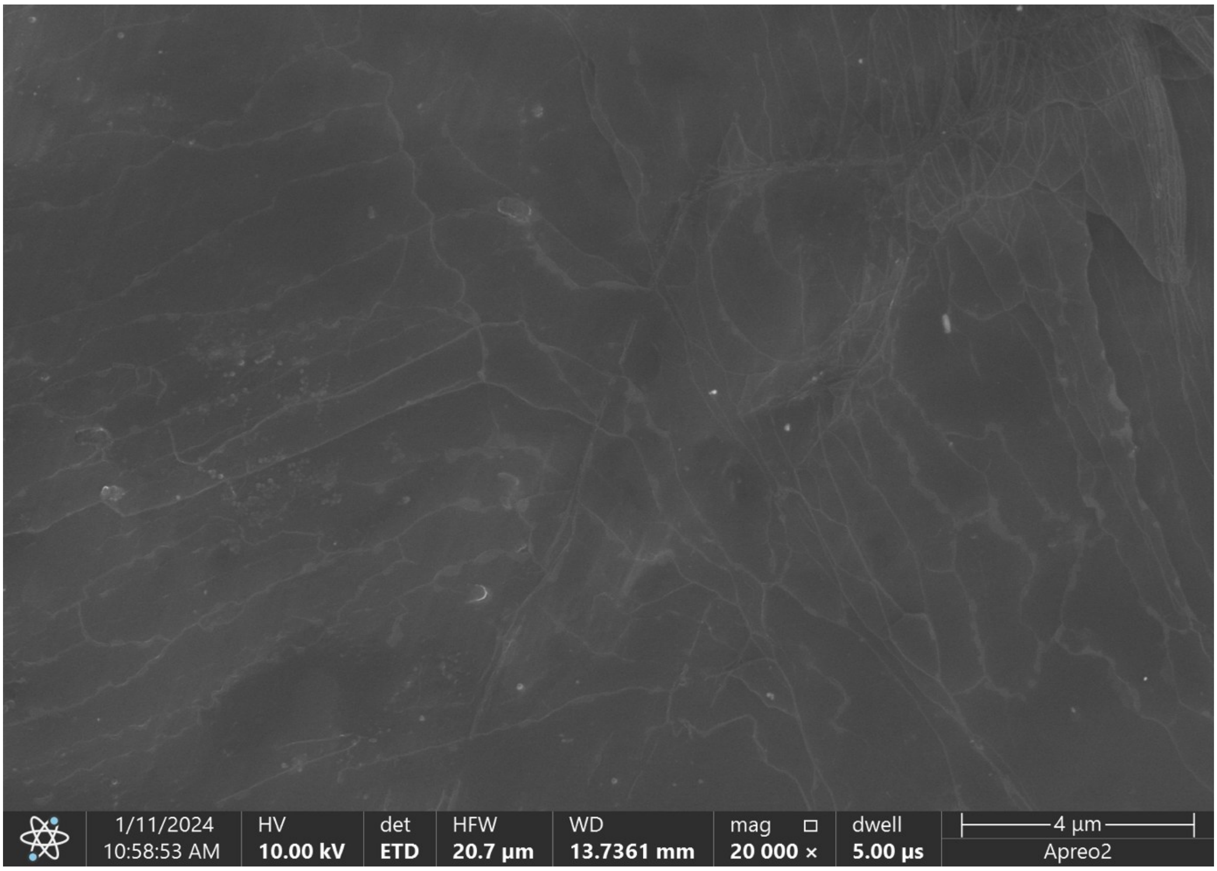

Figure S40. SEM graph of P3HT:DBSA 10:1 mass ratio pellet cross-section (ETD)

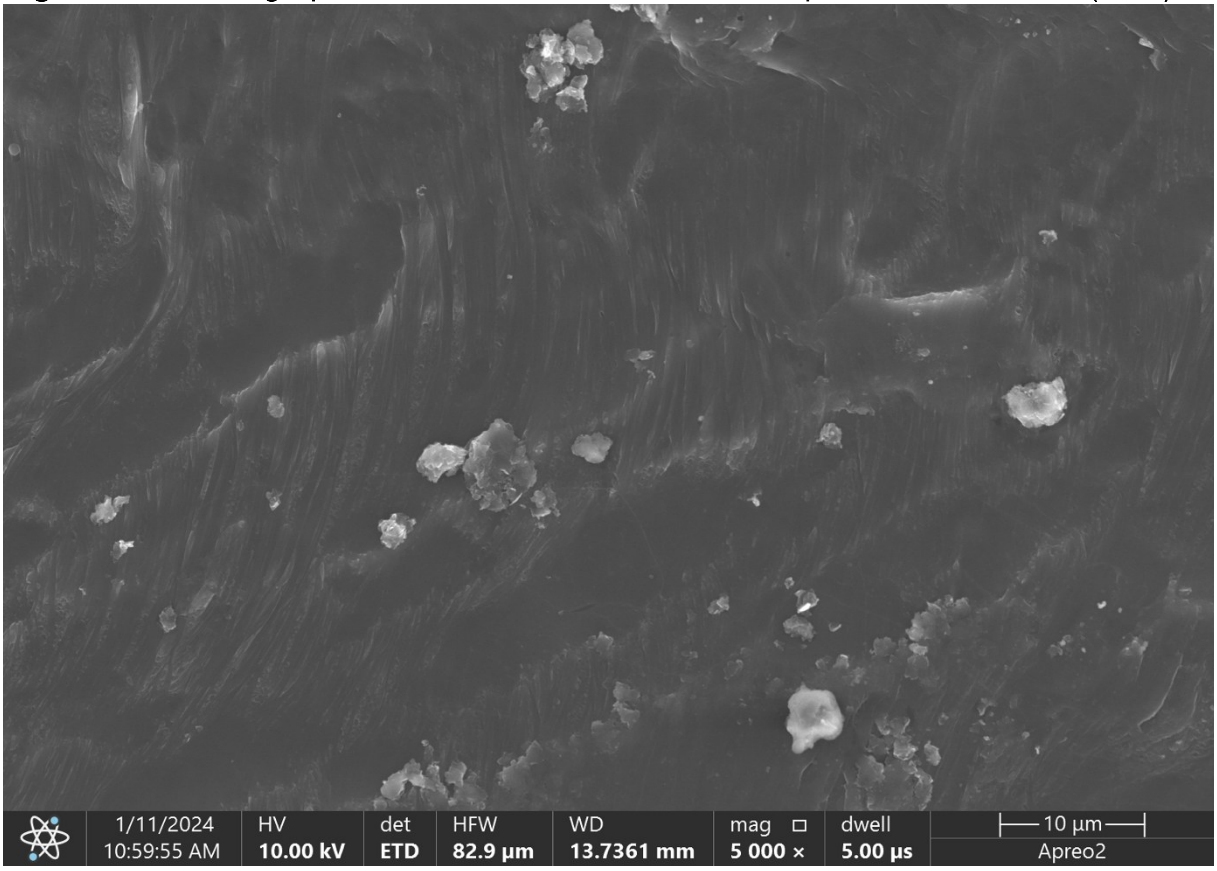

Figure S41. SEM graph of P3HT:DBSA 10:1 mass ratio pellet cross-section (ETD)\

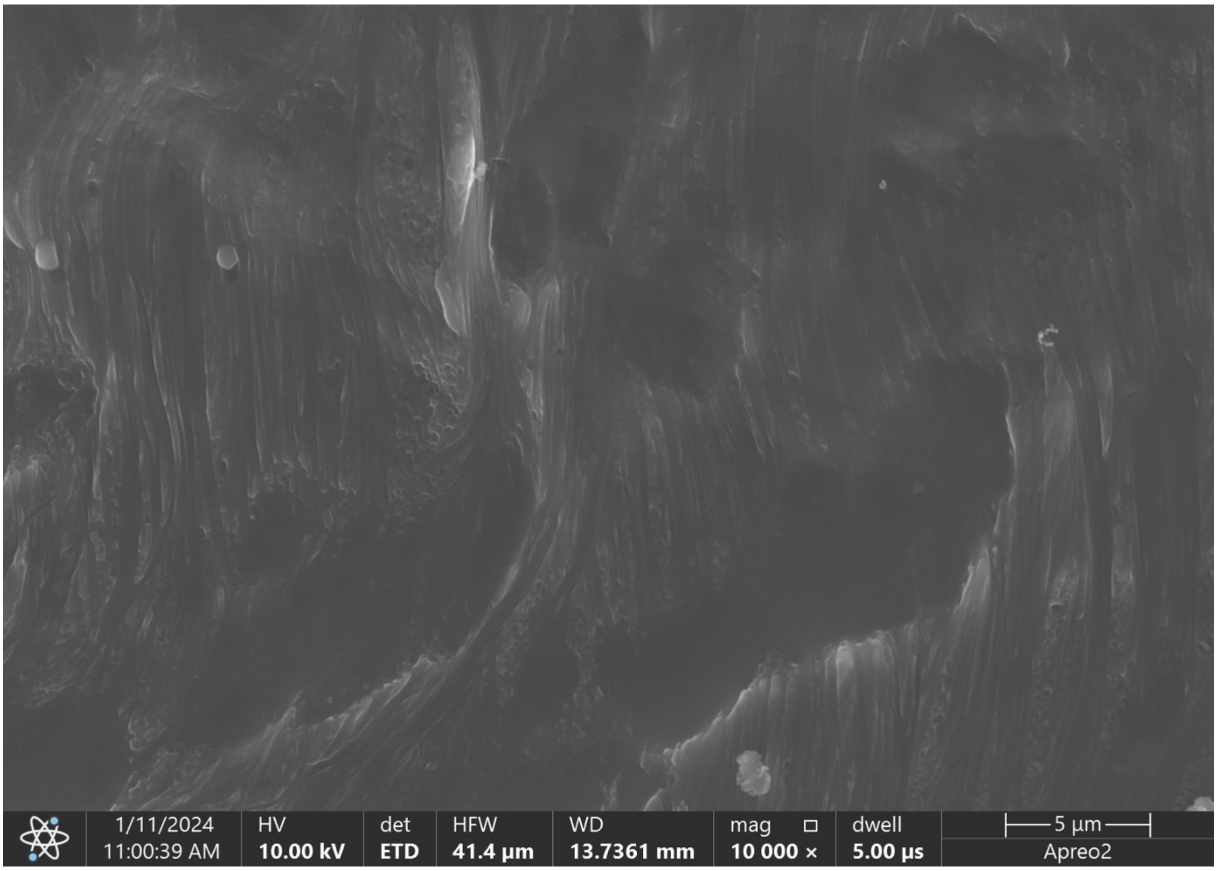

Figure S42. SEM graph of P3HT:DBSA 10:1 mass ratio pellet cross-section (ETD)

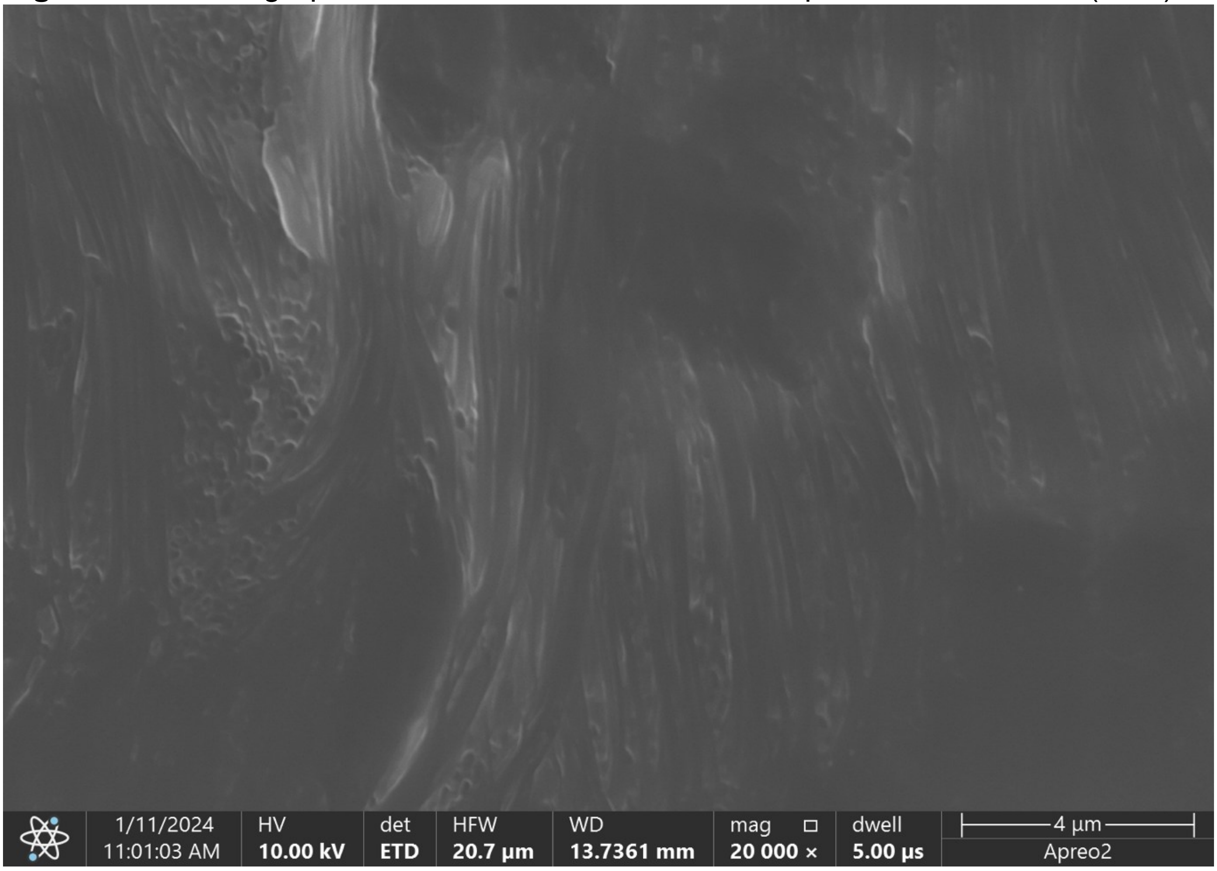

Figure S43. SEM graph of P3HT:DBSA 10:1 mass ratio pellet cross-section (ETD)

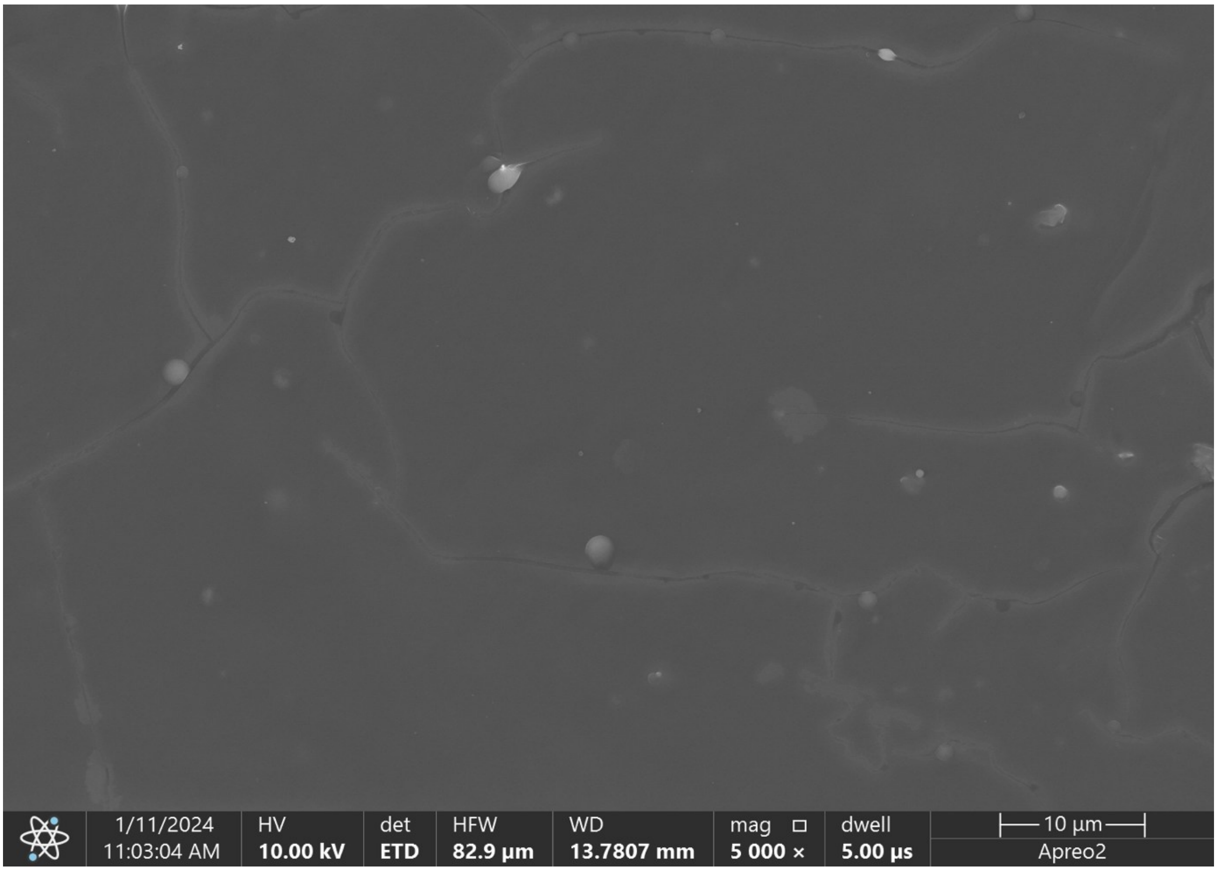

Figure S44. SEM graph of P3HT:DBSA 10:1 mass ratio pellet cross-section (ETD)

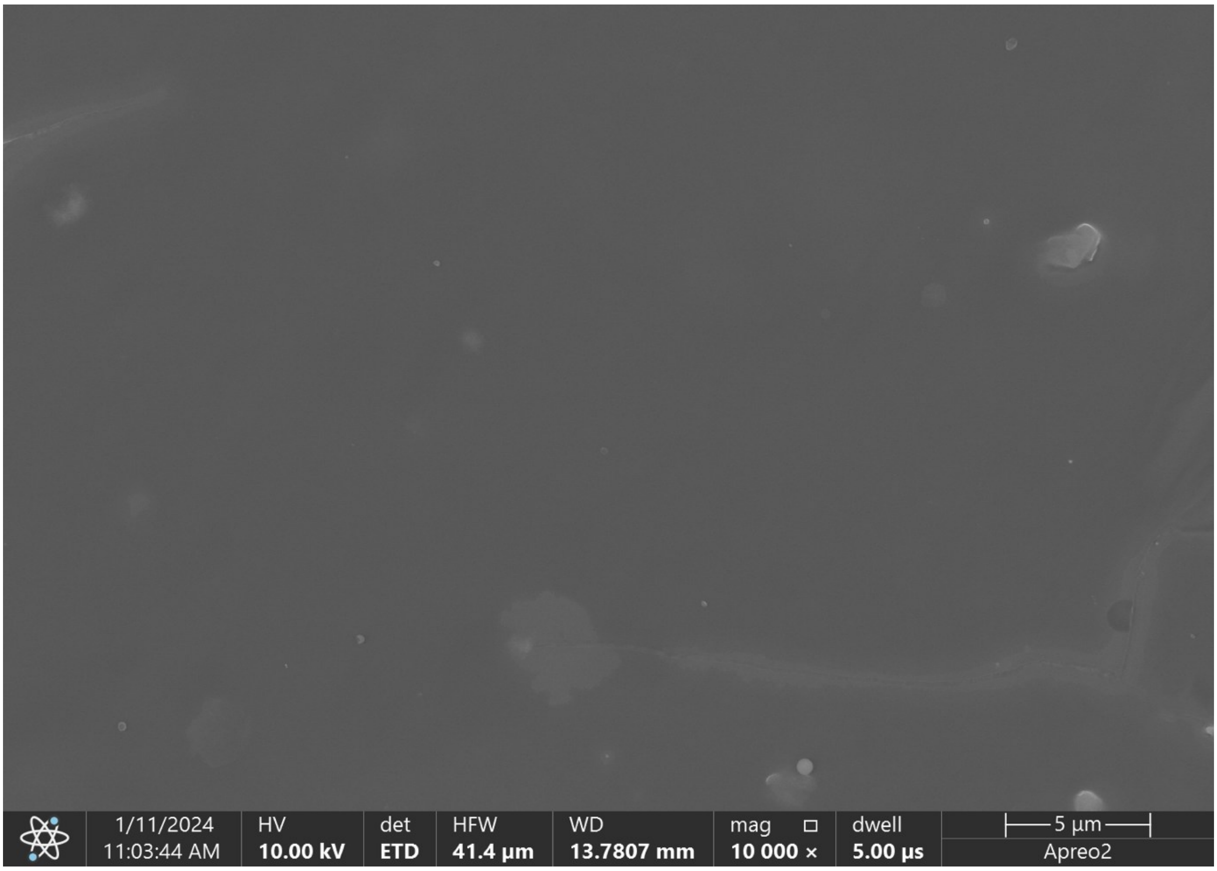

Figure S45. SEM graph of P3HT:DBSA 10:1 mass ratio pellet cross-section (ETD)

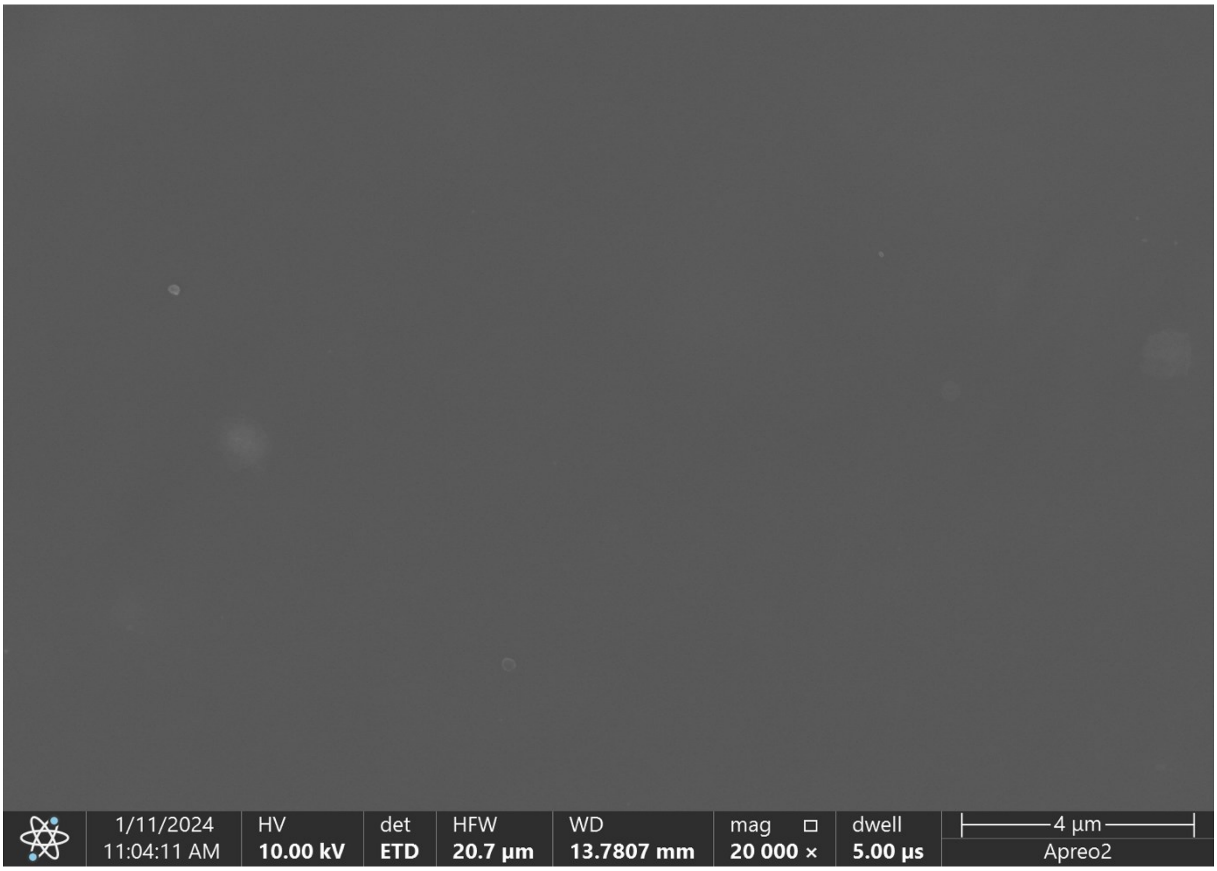

Figure S46. SEM graph of P3HT:DBSA 10:1 mass ratio pellet cross-section (ETD)

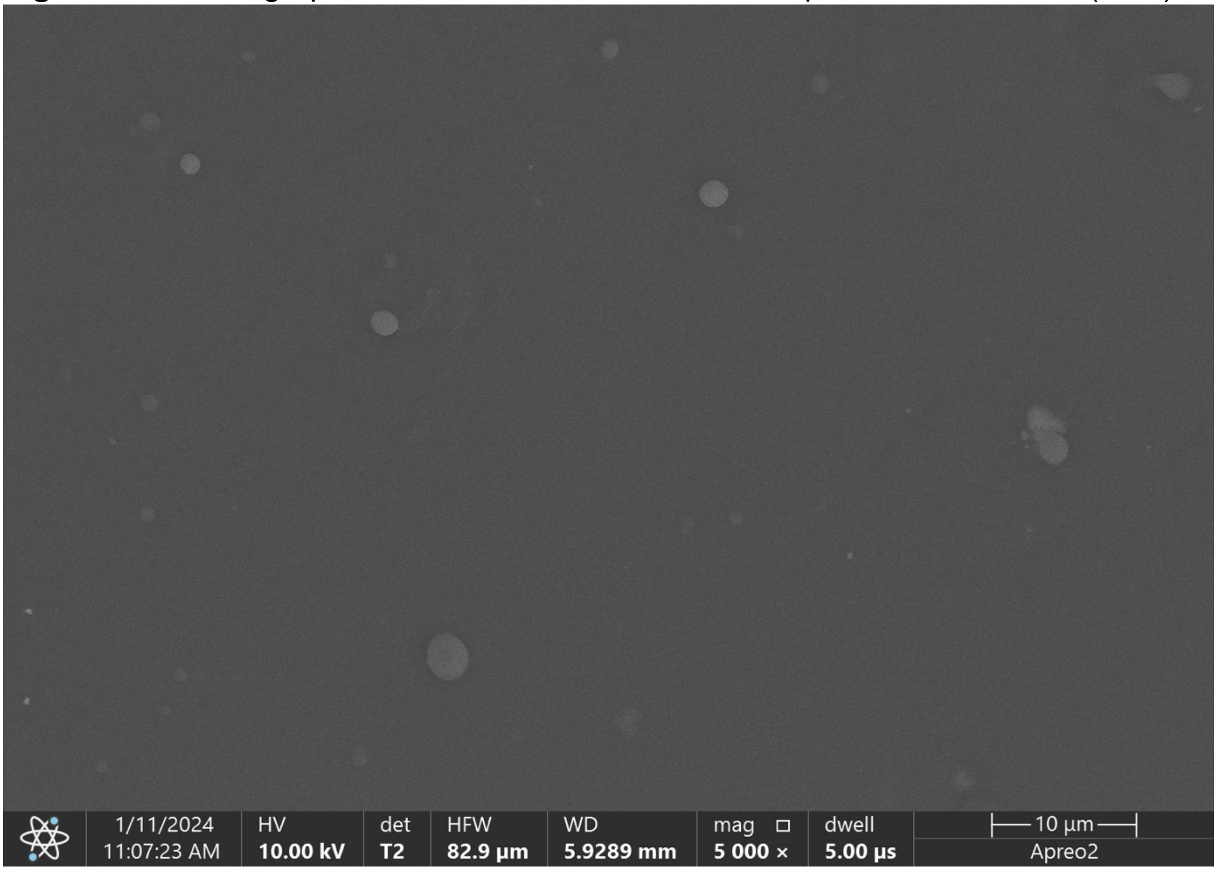

Figure S47. SEM graph of P3HT:DBSA 10:1 mass ratio pellet cross-section (T2)

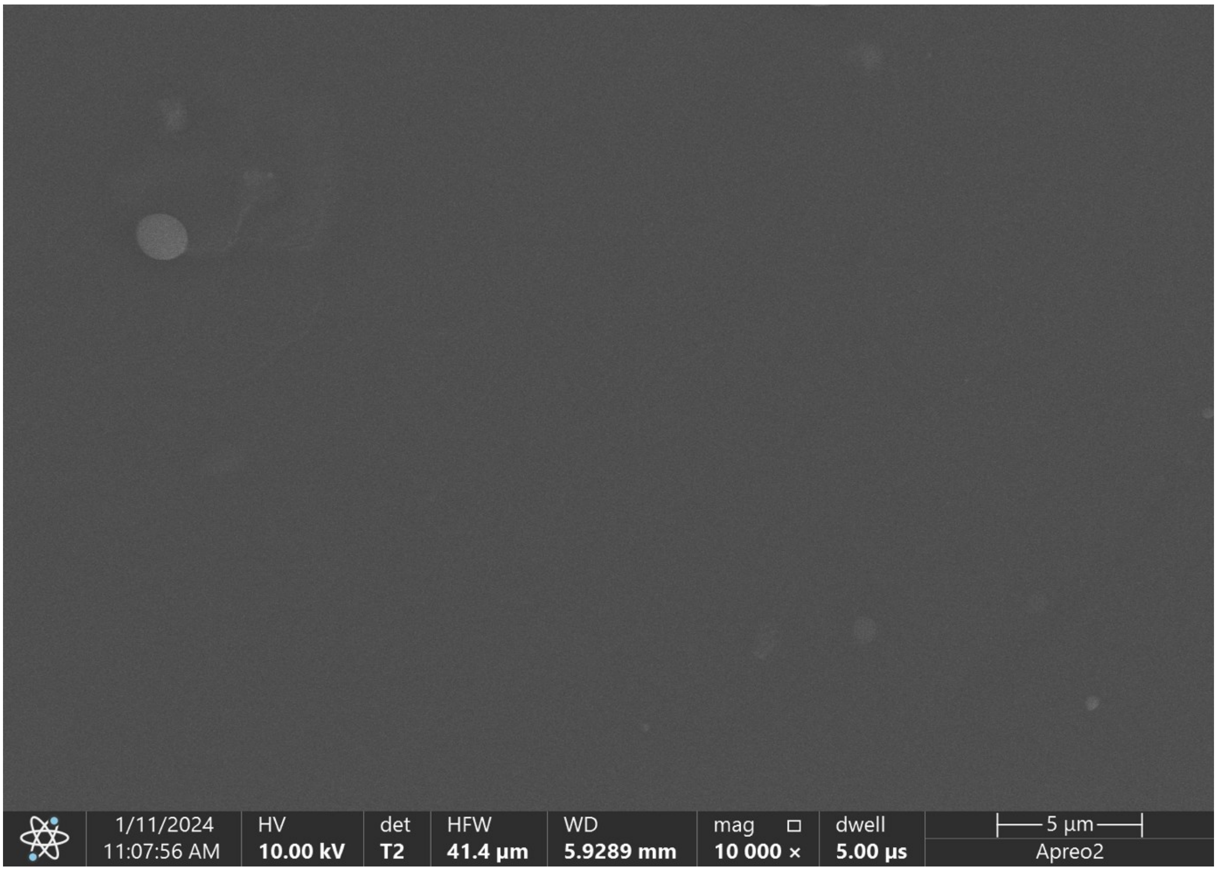

Figure S48. SEM graph of P3HT:DBSA 10:1 mass ratio pellet cross-section (T2)

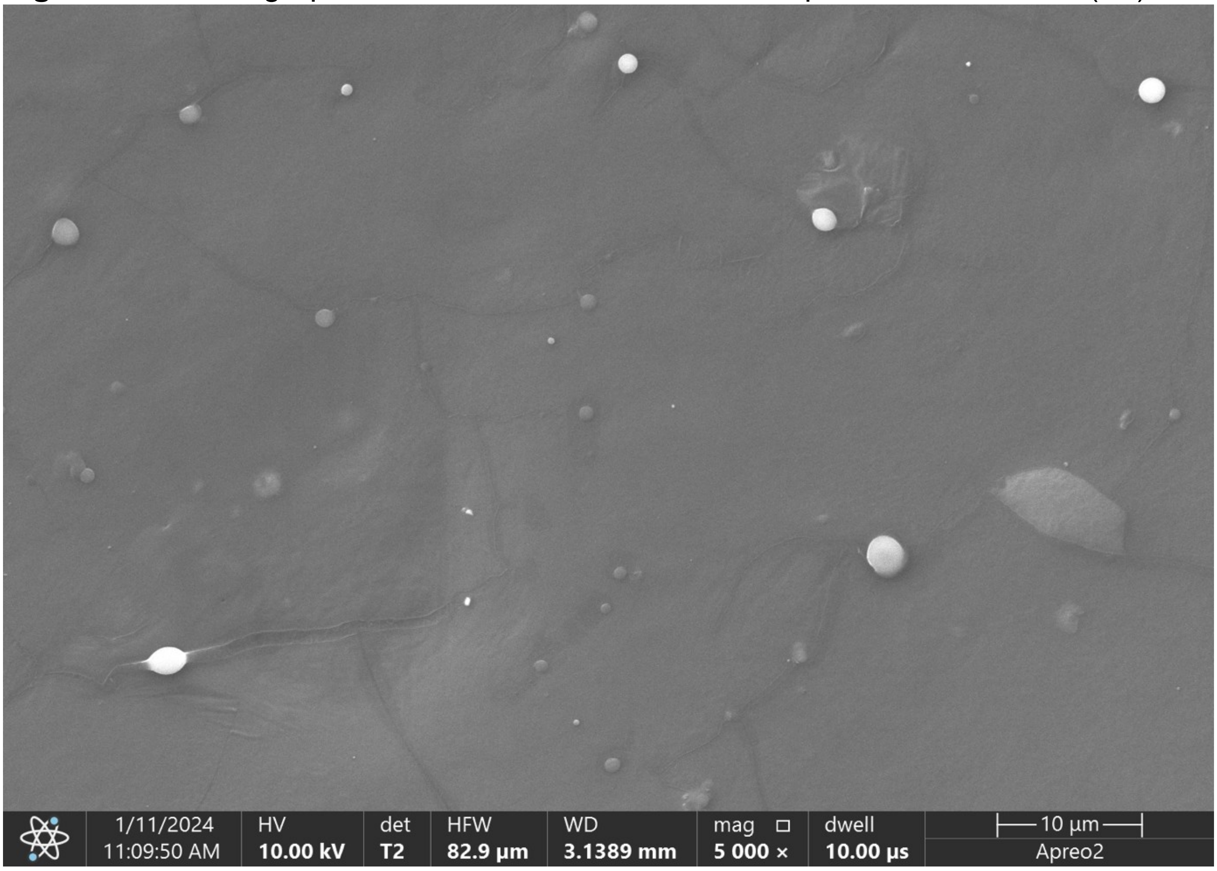

Figure S49. SEM graph of P3HT:DBSA 10:1 mass ratio pellet cross-section (T2)

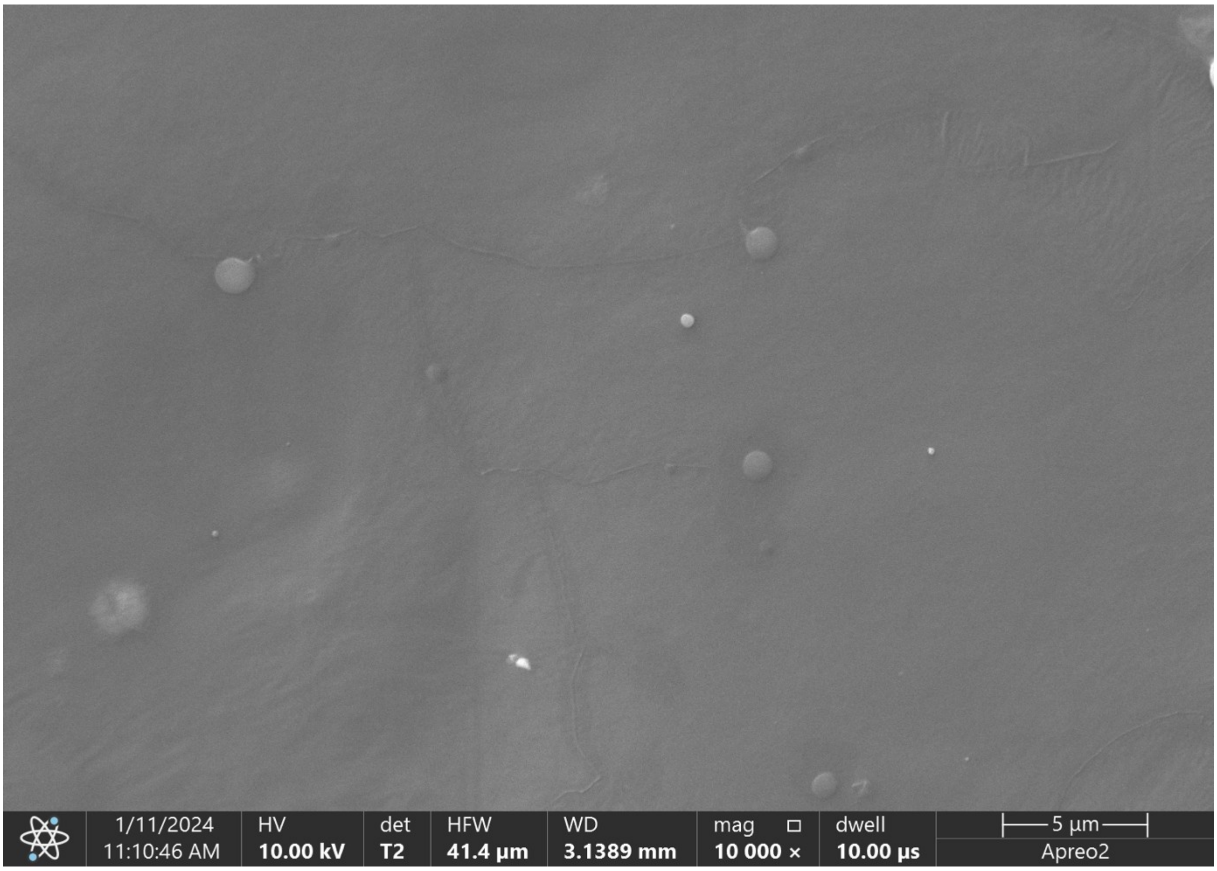

Figure S50. SEM graph of P3HT:DBSA 10:1 mass ratio pellet cross-section (T2)

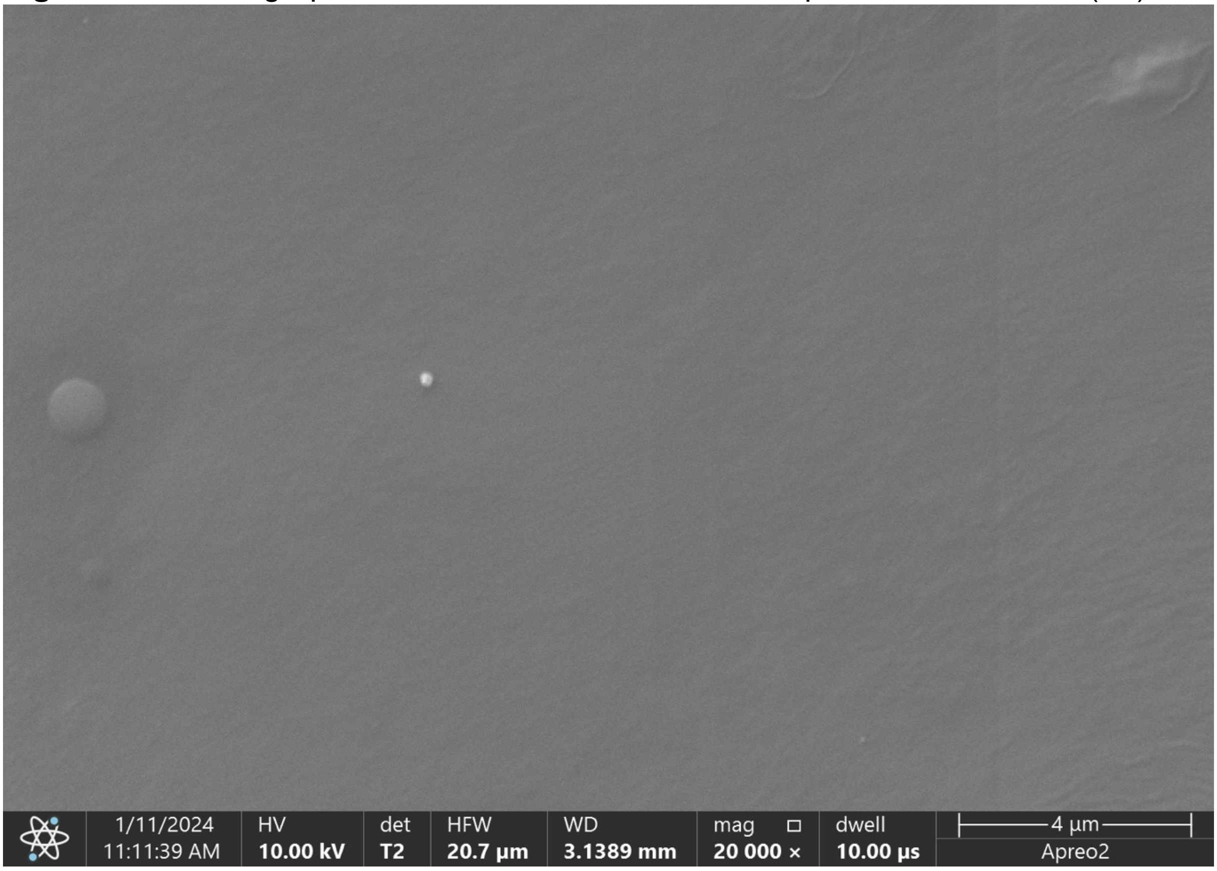

Figure S51. SEM graph of P3HT:DBSA 10:1 mass ratio pellet cross-section (T2)

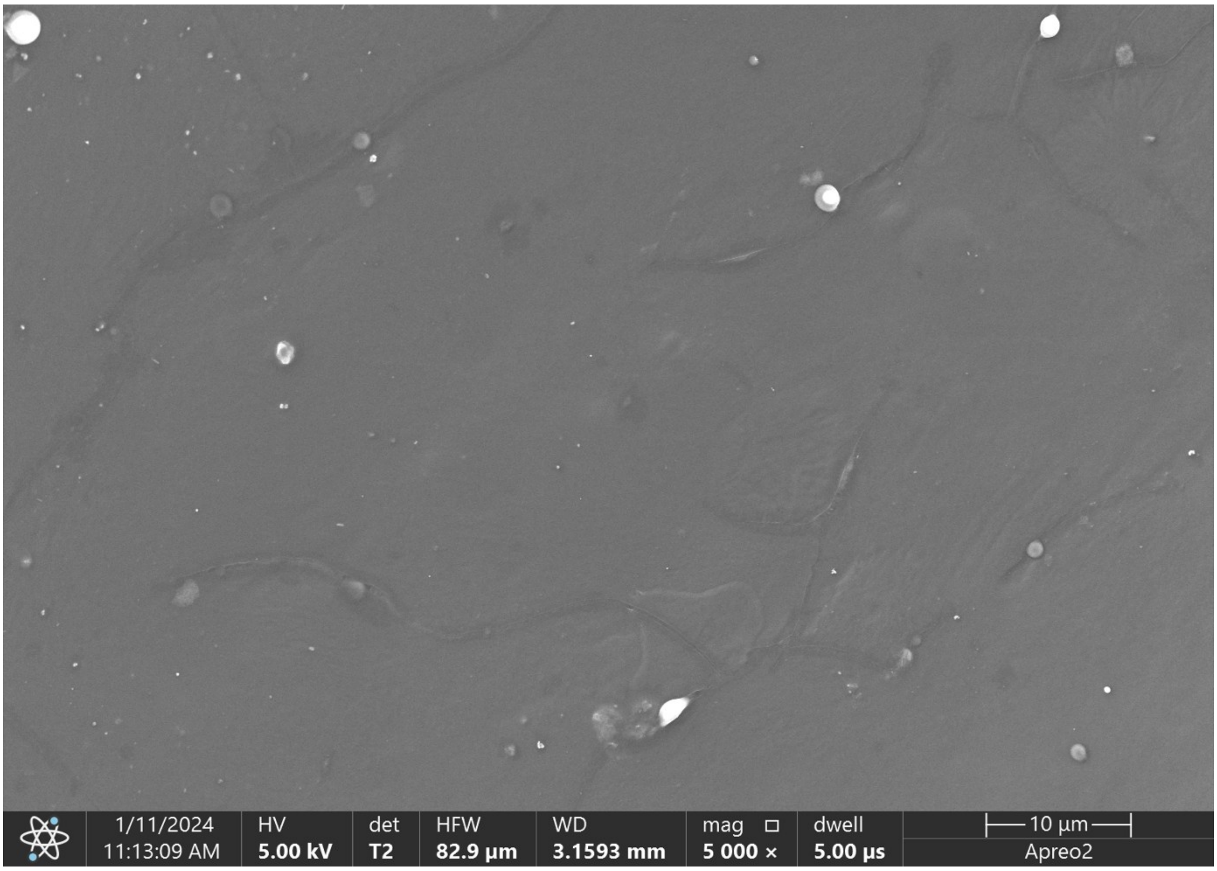

Figure S52. SEM graph of P3HT:DBSA 10:1 mass ratio pellet cross-section (T2)

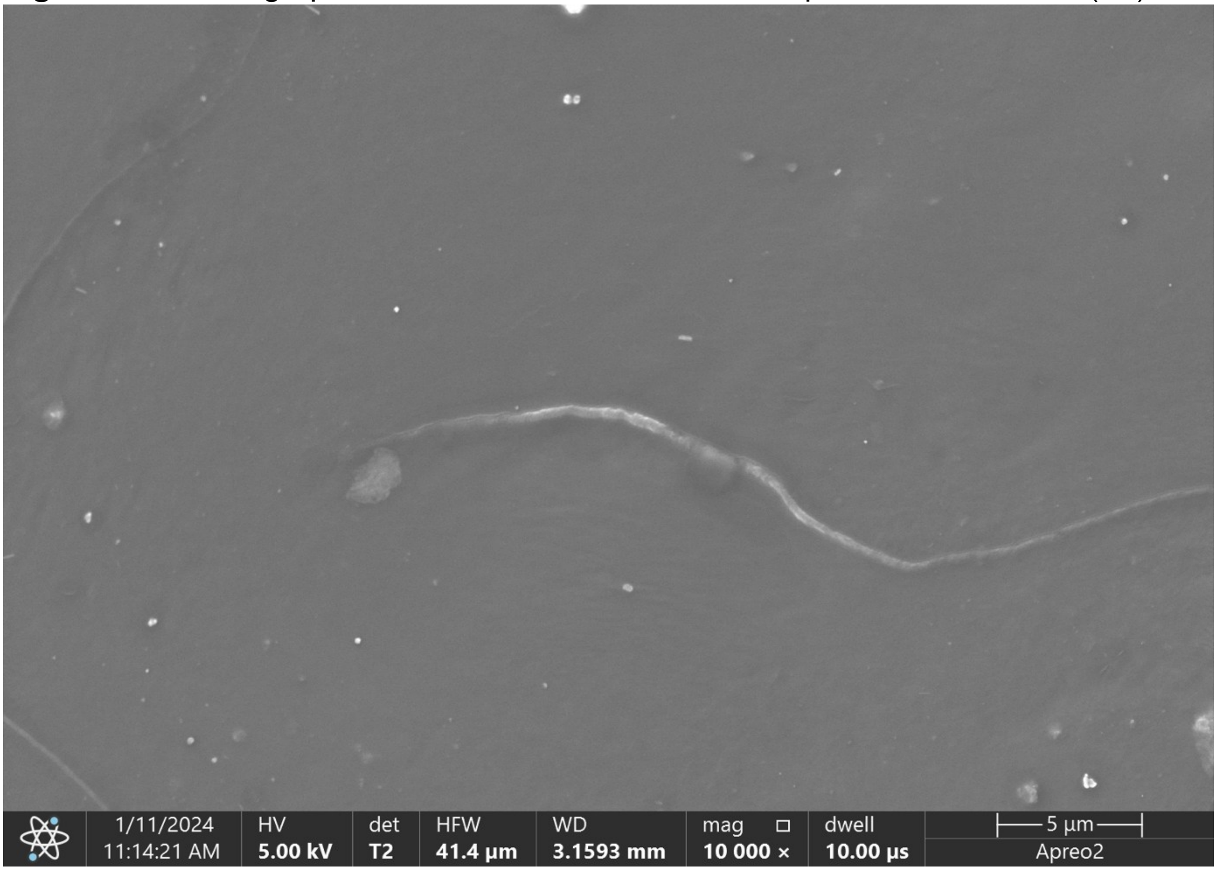

Figure S53. SEM graph of P3HT:DBSA 10:1 mass ratio pellet cross-section (T2)

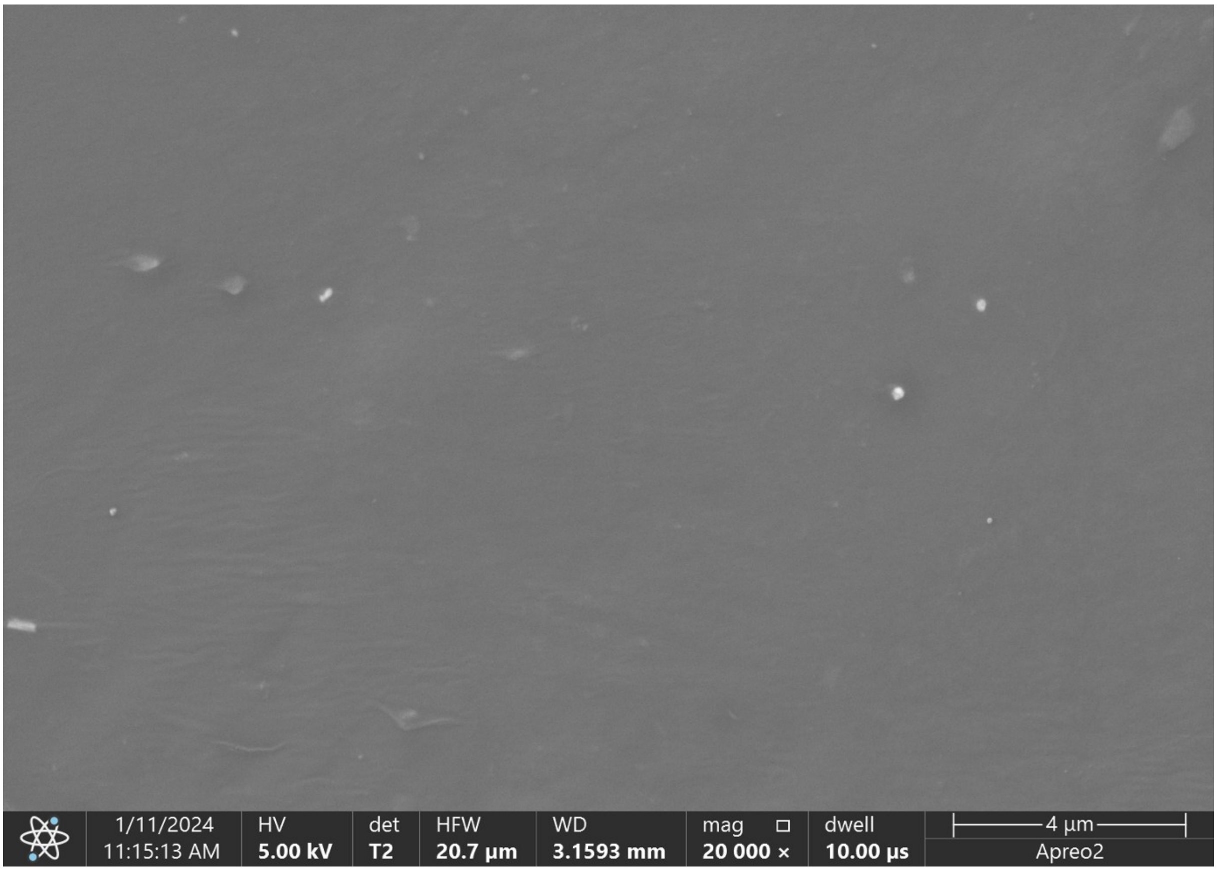

Figure S54. SEM graph of P3HT:DBSA 10:1 mass ratio pellet cross-section (T2)

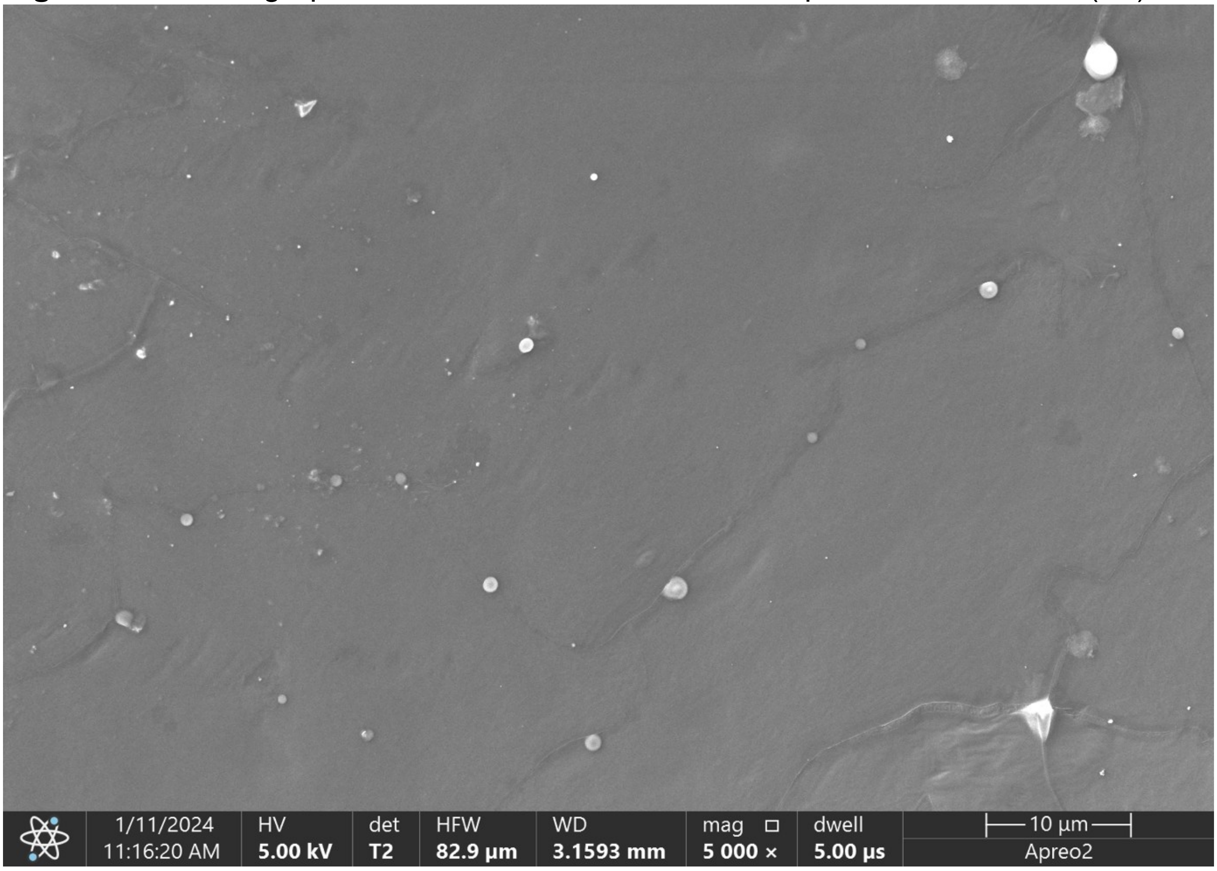

Figure S55. SEM graph of P3HT:DBSA 10:1 mass ratio pellet cross-section (T2)

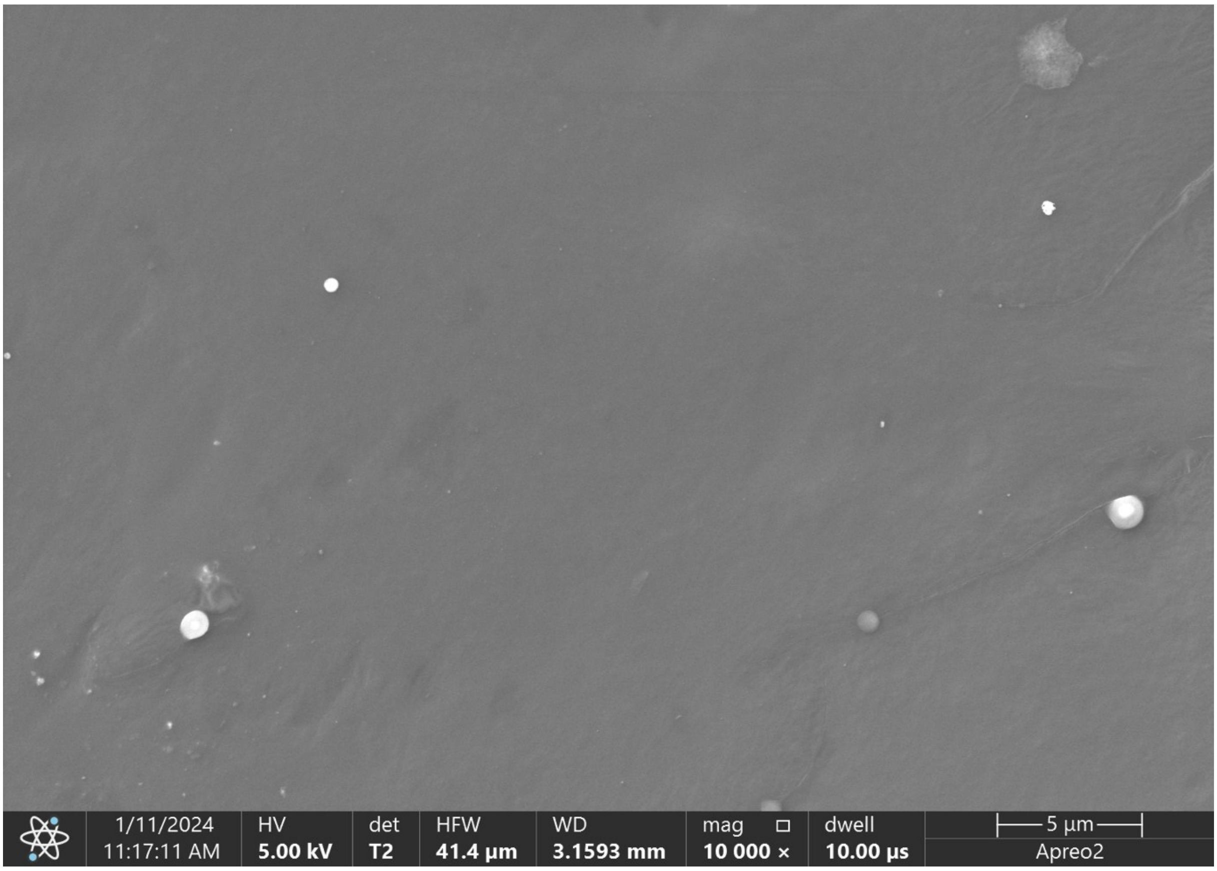

Figure S56. SEM graph of P3HT:DBSA 10:1 mass ratio pellet cross-section (T2)

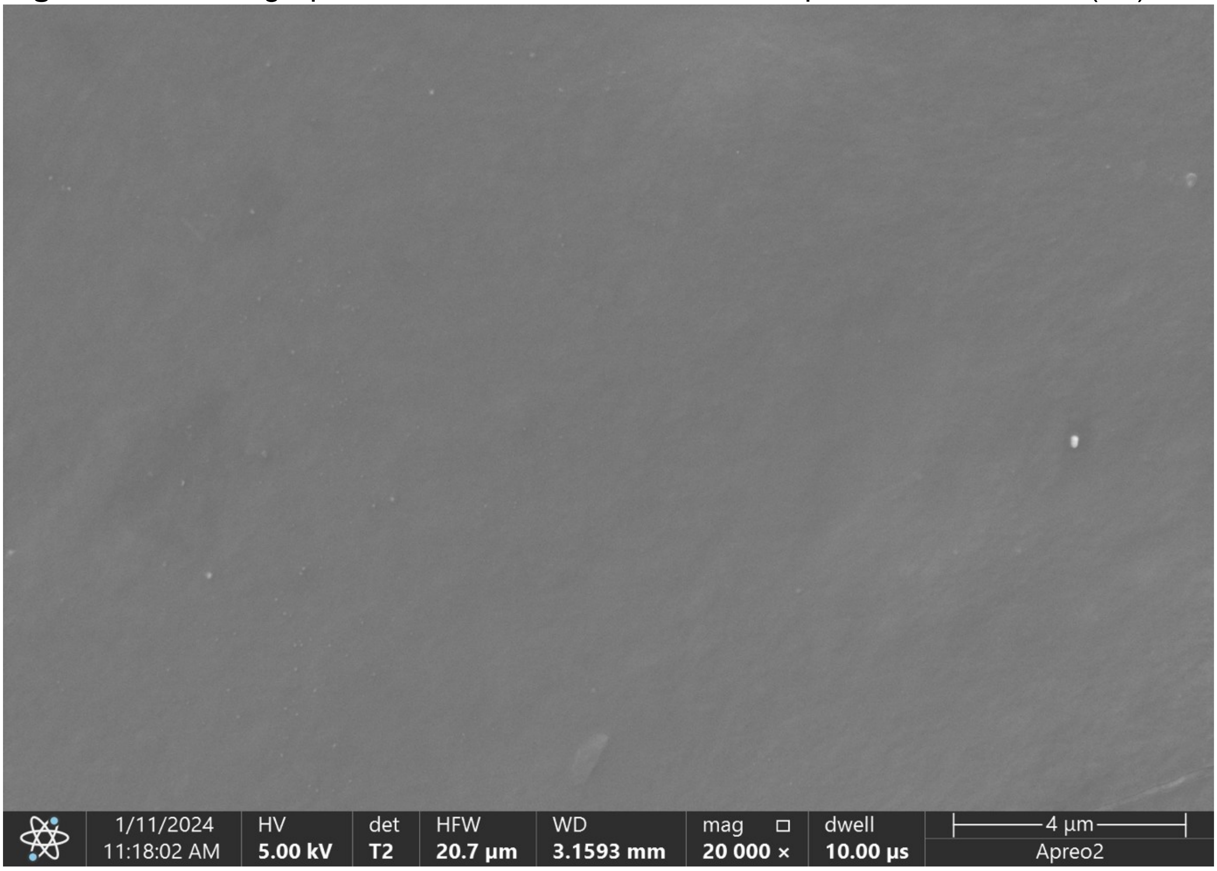

Figure S57. SEM graph of P3HT:DBSA 10:1 mass ratio pellet cross-section (T2)

3. Measurements of ZT

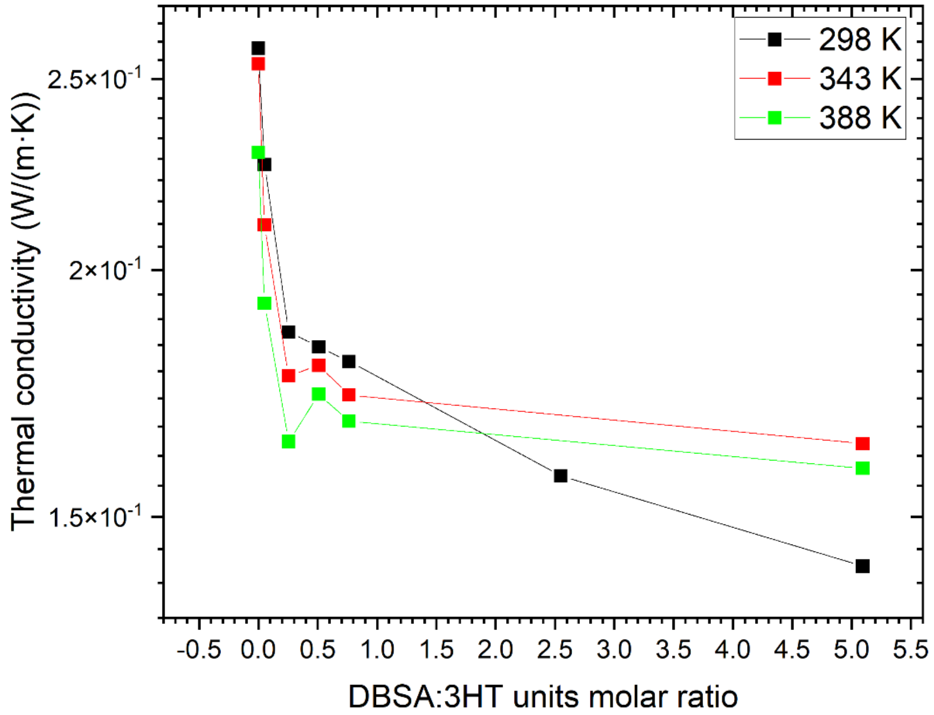

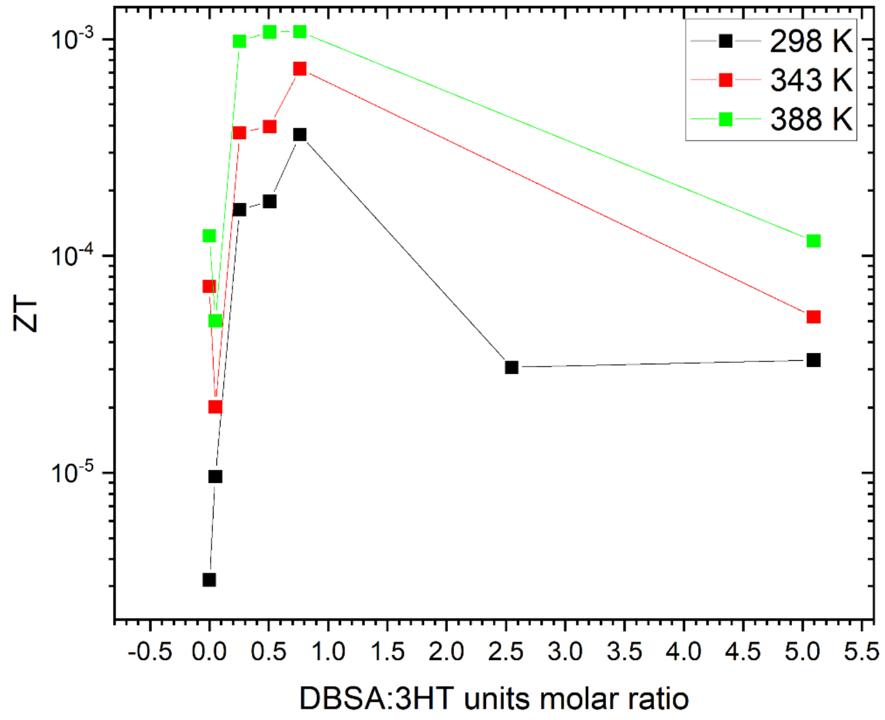

Figure S58. Thermal conductivity and ZT relationship with DBSA and 3-hexylthiophene (3HT) mers molar ratio

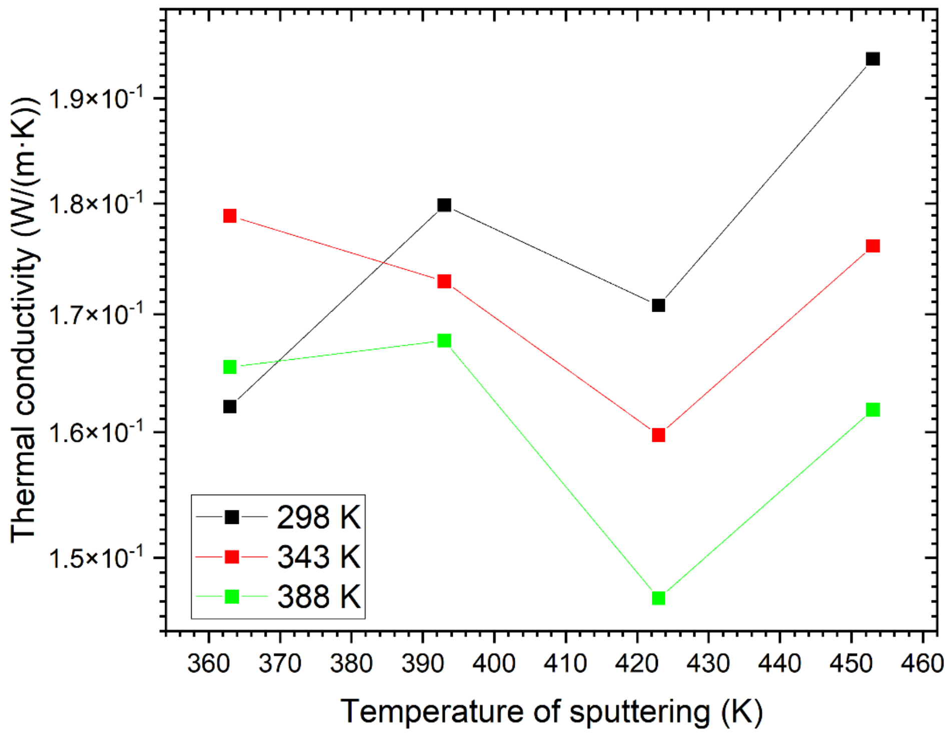

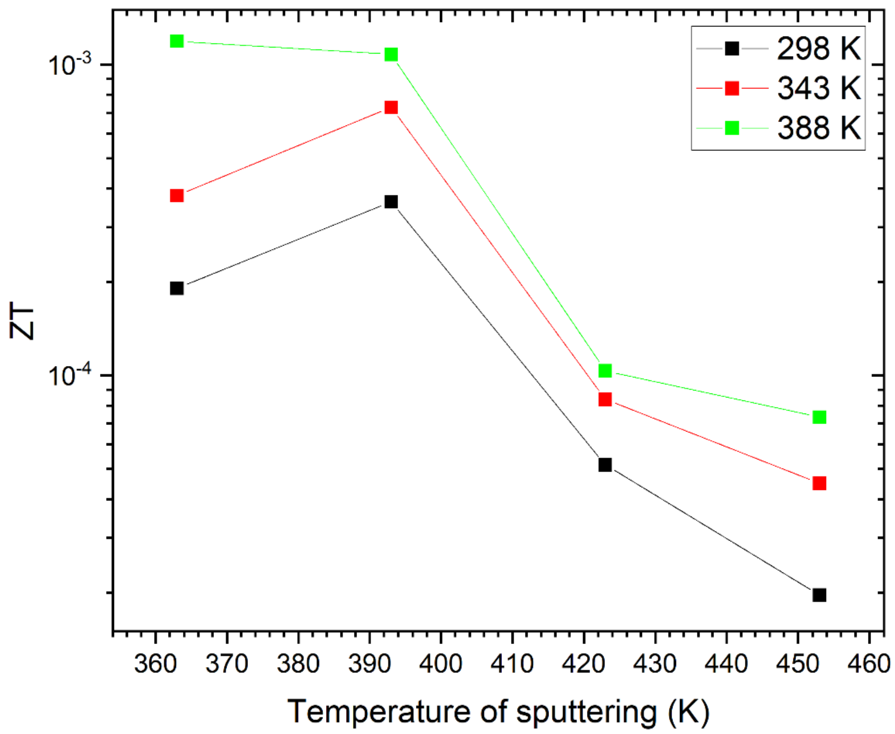

Figure S59. Thermal conductivity and ZT relationship with temperature of sputtering

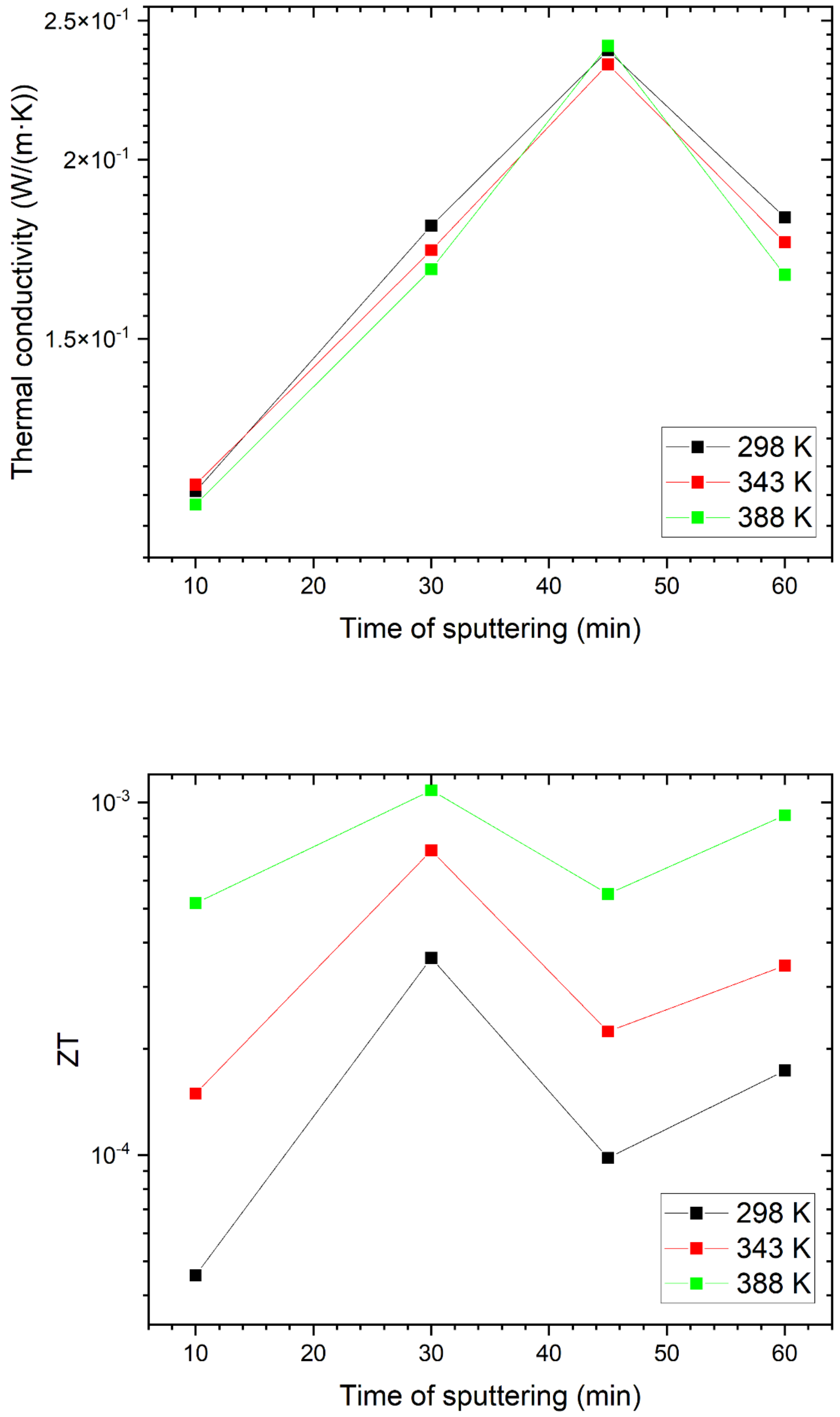

Figure S60. Thermal conductivity and ZT relationship with sputtering time

Table S1. Results of thermoelectric measurements of P3HT:DBSA based pellets in 298 K.

Pellet (mass ratio, sputtering time, and temperature)	Conductivity σ [S·cm^{-1}]	Seebeck coefficient [μV·K^{-1}]	Power factor $\sigma\alpha^2$ [μW·m^{-1}·K^{-2}]	Thermal conductivity κ [μW·m^{-1}·K^{-1}]	ZT
P3HT:DBSA 1:0, 30 min, 120 ºC	$4.98 \cdot 10^{-5}$	745	0.0028	0.259	$3.208 \cdot 10^{-6}$
P3HT:DBSA 10:1, 30 min, 120 ºC	$6.64 \cdot 10^{-4}$	558	0.0207	0.226	$9.62 \cdot 10^{-6}$
P3HT:DBSA 2:1, 30 min, 120 ºC	0.0079	357	0.101	0.186	$1.63 \cdot 10^{-4}$
P3HT:DBSA 1:1, 30 min, 120 ºC	0.016	263	0.108	0.183	$1.78 \cdot 10^{-4}$
P3HT:DBSA 2:3, 30 min, 120 ºC	0.103	145	0.22	0.180	$3.62 \cdot 10^{-4}$
P3HT:DBSA 1:5, 30 min, 120 ºC	0.0029	235	0.016	0.157	$3.063 \cdot 10^{-5}$
P3HT:DBSA 1:10, 30 min, 120 ºC	0.0022	265	0.016	0.142	$3.303 \cdot 10^{-5}$
P3HT:DBSA 2:3, 30 min, 90 ºC	0.035	204	0.103	0.162	$1.908 \cdot 10^{-4}$
P3HT:DBSA 2:3, 30 min, 150 ºC	0.0022	366	0.029	0.1708	$5.16 \cdot 10^{-5}$
P3HT:DBSA 2:3, 30 min, 180 ºC	$9.907 \cdot 10^{-4}$	357	0.013	0.194	$1.96 \cdot 10^{-5}$
P3HT:DBSA 2:3, 10 min, 120 ºC	$6.23 \cdot 10^{-4}$	534	0.018	0.117	$4.56 \cdot 10^{-5}$

P3HT:DBSA 2:3, 45 min, 120 ºC	0.0106	270	0.078	0.238	$9.805\cdot10^{-5}$
P3HT:DBSA 2:3, 60 min, 120 ºC	0.022	219	0.105	0.182	$1.74\cdot10^{-4}$

Table S2. Results of thermoelectric measurements of P3HT:DBSA based pellets in 343 K.

Pellet (mass ratio, sputtering time, and temperature)	Conductivity σ [$S\cdot cm^{-1}$]	Seebeck coefficient [$\mu V\cdot K^{-1}$]	Power factor $\sigma\alpha^2$ [$\mu W\cdot m^{-1}\cdot K^{-2}$]	Thermal conductivity κ [$\mu W\cdot m^{-1}\cdot K^{-1}$]	ZT
P3HT:DBSA 1:0, 30 min, 120 ºC	$1.67\cdot10^{-5}$	125	$2.60\cdot10^{-5}$	0.254	$7.24\cdot10^{-5}$
P3HT:DBSA 10:1, 30 min, 120 ºC	0.0023	460	0.049	0.2109	$2.011\cdot10^{-5}$
P3HT:DBSA 2:1, 30 min, 120 ºC	0.021	300	0.19	0.177	$3.70\cdot10^{-4}$
P3HT:DBSA 1:1, 30 min, 120 ºC	0.0408	225	0.206	0.179	$3.95\cdot10^{-4}$
P3HT:DBSA 2:3, 30 min, 120 ºC	0.20	136	0.37	0.173	$7.30\cdot10^{-4}$
P3HT:DBSA 1:5, 30 min, 120 ºC	0.022	172	0.065		
P3HT:DBSA 1:10, 30 min, 120 ºC	0.00503	222	0.025	0.163	$5.23\cdot10^{-5}$
P3HT:DBSA 2:3, 30 min, 90 ºC	0.059	183	0.20	0.179	$3.79\cdot10^{-4}$

P3HT:DBSA 2:3, 30 min, 150 ºC	0.0030	363	0.039	0.160	$8.37 \cdot 10^{-5}$
P3HT:DBSA 2:3, 30 min, 180 ºC	0.0018	357	0.023	0.176	$4.50 \cdot 10^{-5}$
P3HT:DBSA 2:3, 10 min, 120 ºC	0.0032	405	0.052	0.119	$1.49 \cdot 10^{-4}$
P3HT:DBSA 2:3, 45 min, 120 ºC	0.026	241	0.15	0.233	$2.24 \cdot 10^{-4}$
P3HT:DBSA 2:3, 60 min, 120 ºC	0.042	203	0.18	0.175	$3.44 \cdot 10^{-4}$

Table S3. Results of thermoelectric measurements of P3HT:DBSA based pellets in 388 K.

Pellet (mass ratio, sputtering time, and temperature)	Conductivity σ [$S \cdot cm^{-1}$]	Seebeck coefficient [$\mu V \cdot K^{-1}$]	Power factor $\sigma\alpha^2$ [$\mu W \cdot m^{-1} \cdot K^{-2}$]	Thermal conductivity κ [$\mu W \cdot m^{-1} \cdot K^{-1}$]	ZT
P3HT:DBSA 1:0, 30 min, 120 ºC	$1.19 \cdot 10^{-5}$	-103	$1.25 \cdot 10^{-5}$	0.229	$1.24 \cdot 10^{-4}$
P3HT:DBSA 10:1, 30 min, 120 ºC	0.0076	399	0.12	0.192	$5.016 \cdot 10^{-5}$
P3HT:DBSA 2:1, 30 min, 120 ºC	0.074	235	0.41	0.164	$9.75 \cdot 10^{-4}$
P3HT:DBSA 1:1, 30 min, 120 ºC	0.16	172	0.48	0.173	0.00108
P3HT:DBSA 2:3, 30 min, 120 ºC	0.28	129	0.47	0.168	0.00108

P3HT:DBSA 1:5, 30 min, 120 ºC	0.047	159	0.12		
P3HT:DBSA 1:10, 30 min, 120 ºC	0.025	139	0.048	0.159	$1.17\cdot10^{-4}$
P3HT:DBSA 2:3, 30 min, 90 ºC	0.26	138	0.508	0.165	0.00119
P3HT:DBSA 2:3, 30 min, 150 ºC	0.0032	352	0.039	0.147	$1.035\cdot10^{-4}$
P3HT:DBSA 2:3, 30 min, 180 ºC	0.0031	314	0.0306	0.162	$7.34\cdot10^{-5}$
P3HT:DBSA 2:3, 10 min, 120 ºC	0.014	336	0.15	0.115	$5.17\cdot10^{-4}$
P3HT:DBSA 2:3, 45 min, 120 ºC	0.093	192	0.34	0.24007	$5.50\cdot10^{-4}$
P3HT:DBSA 2:3, 60 min, 120 ºC	0.15	161	0.39	0.166	$9.18\cdot10^{-4}$

4. Thermoelectric properties

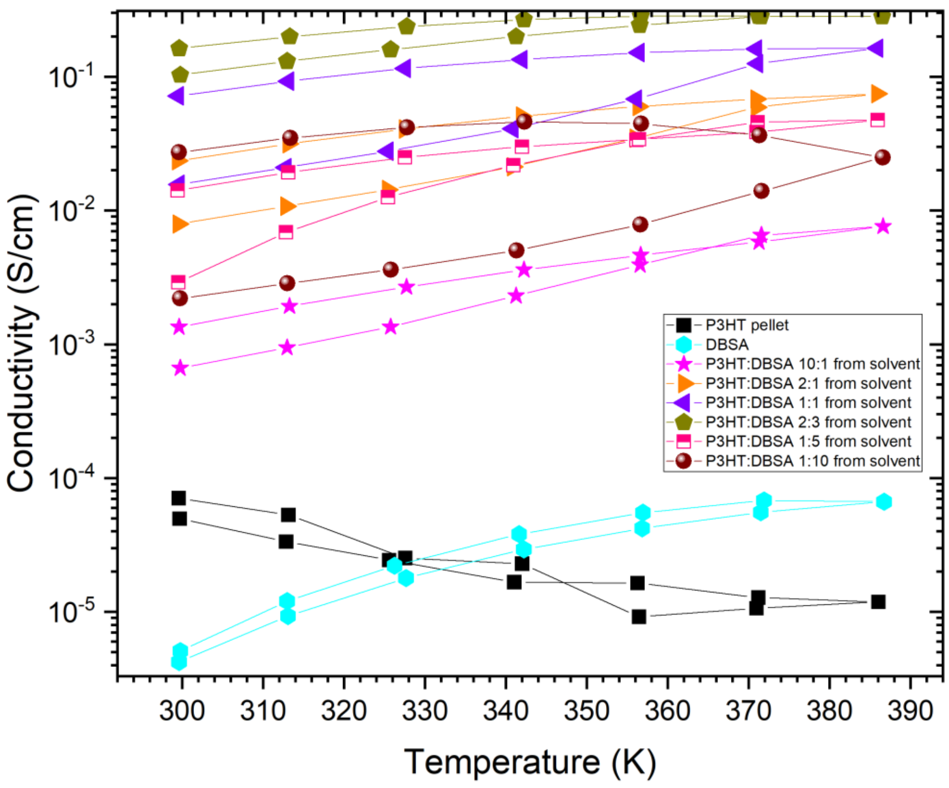

Figure S61. Relationship of pellets conductivity with temperature of measurement

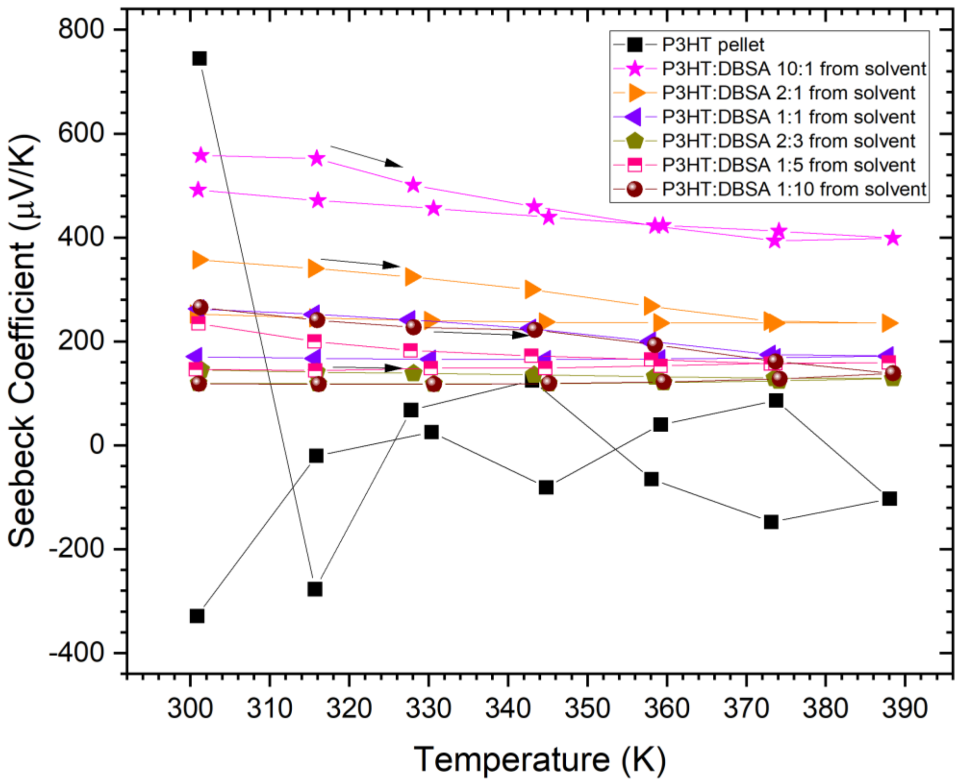

Figure S62. Relationship of pellets Seebeck coefficient with temperature of measurement

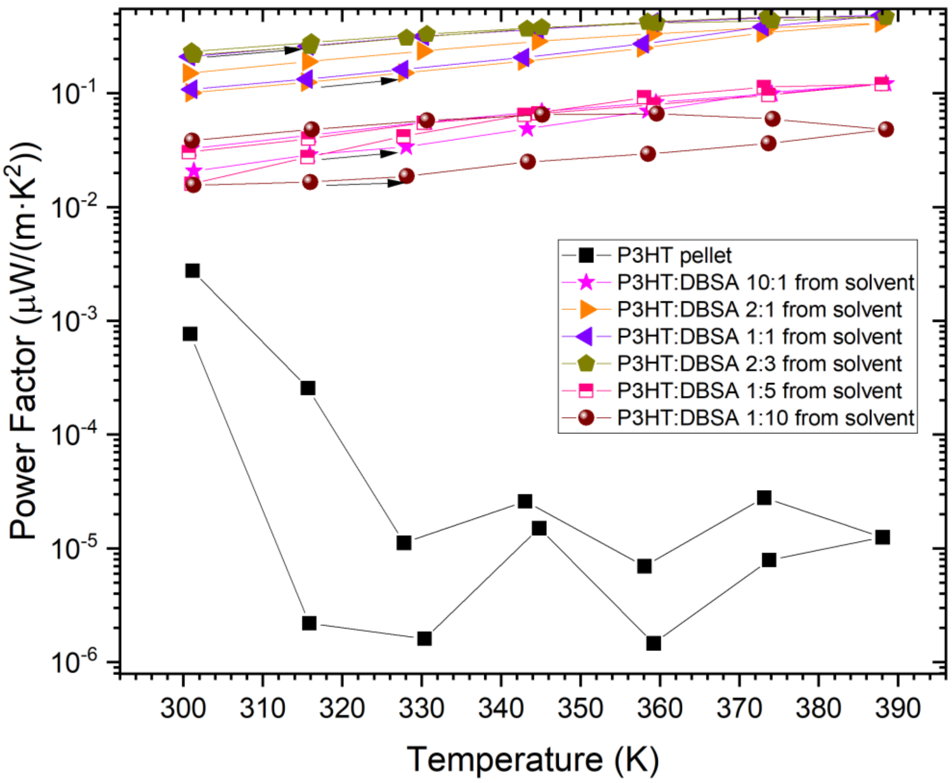

Figure S63. Relationship of pellets power factor with temperature of measurement

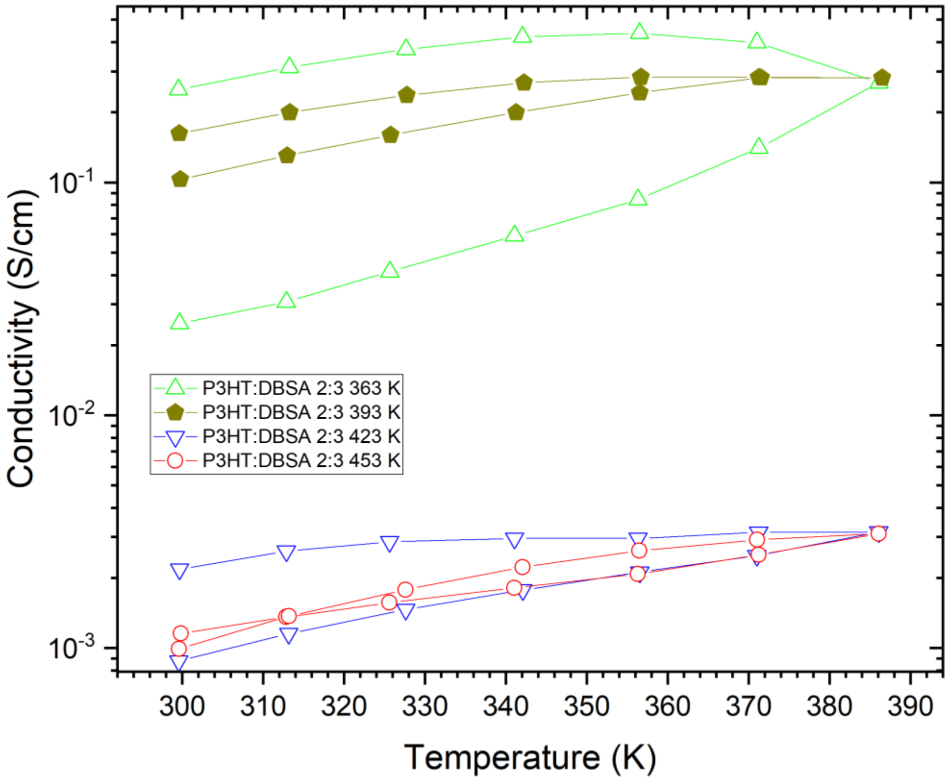

Figure S64. Relationship of pellets sputtered in different temperature conductivity with temperature of measurement

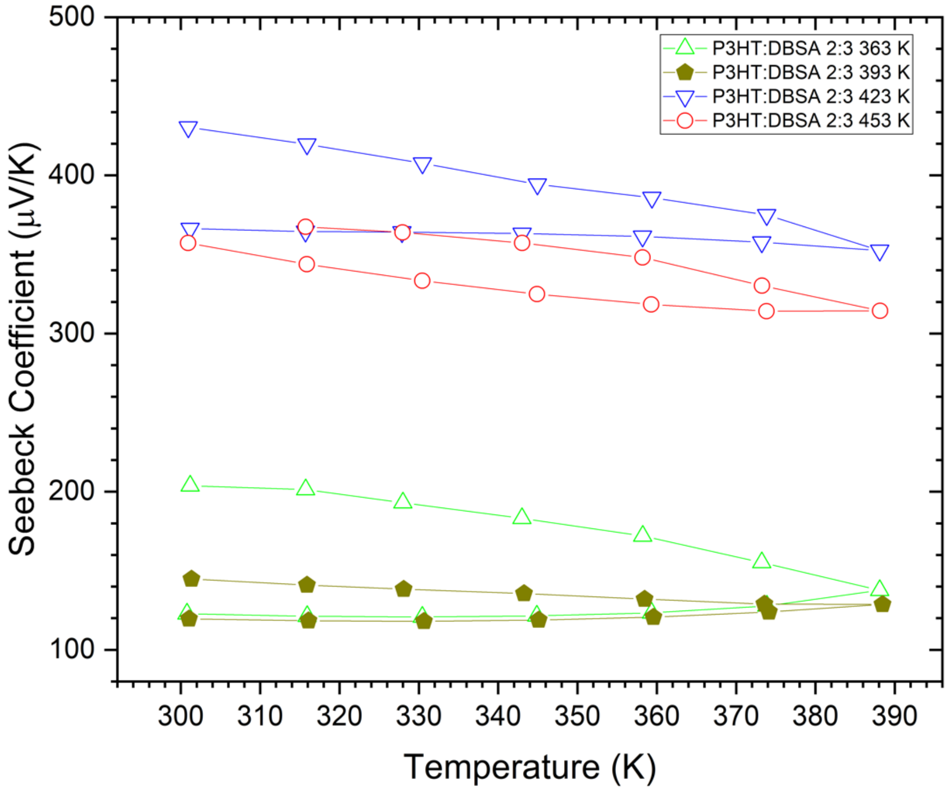

Figure S65. Relationship of pellets sputtered in different temperature Seebeck coefficient with temperature of measurement

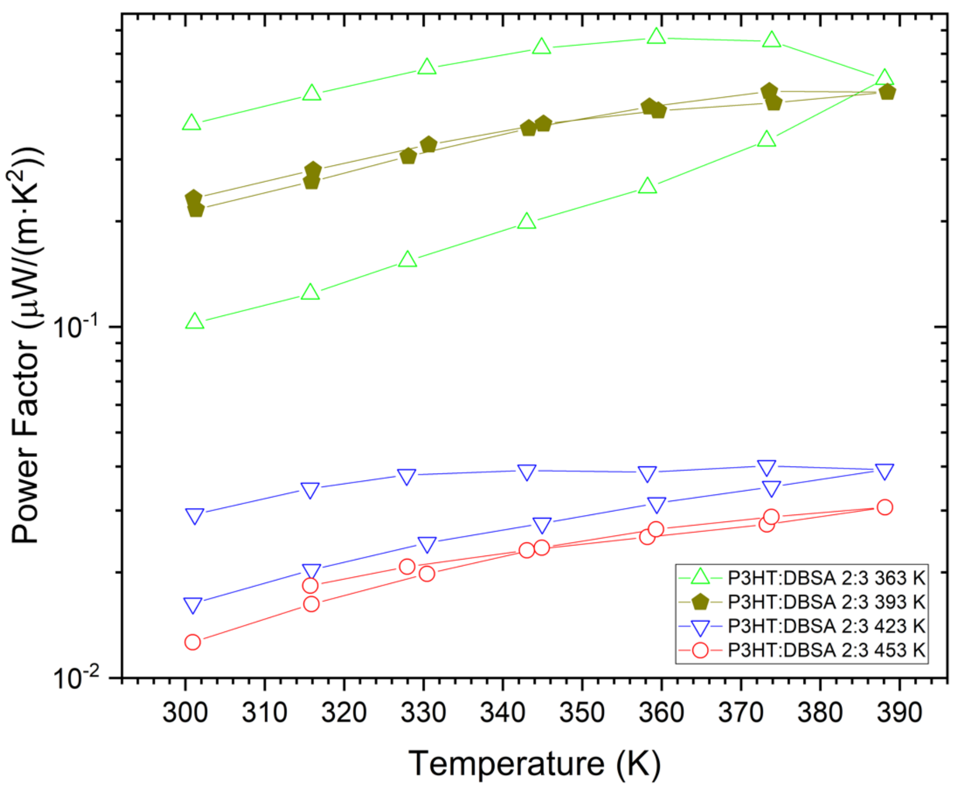

Figure S66. Relationship of pellets sputtered in different temperature power factor with temperature of measurement

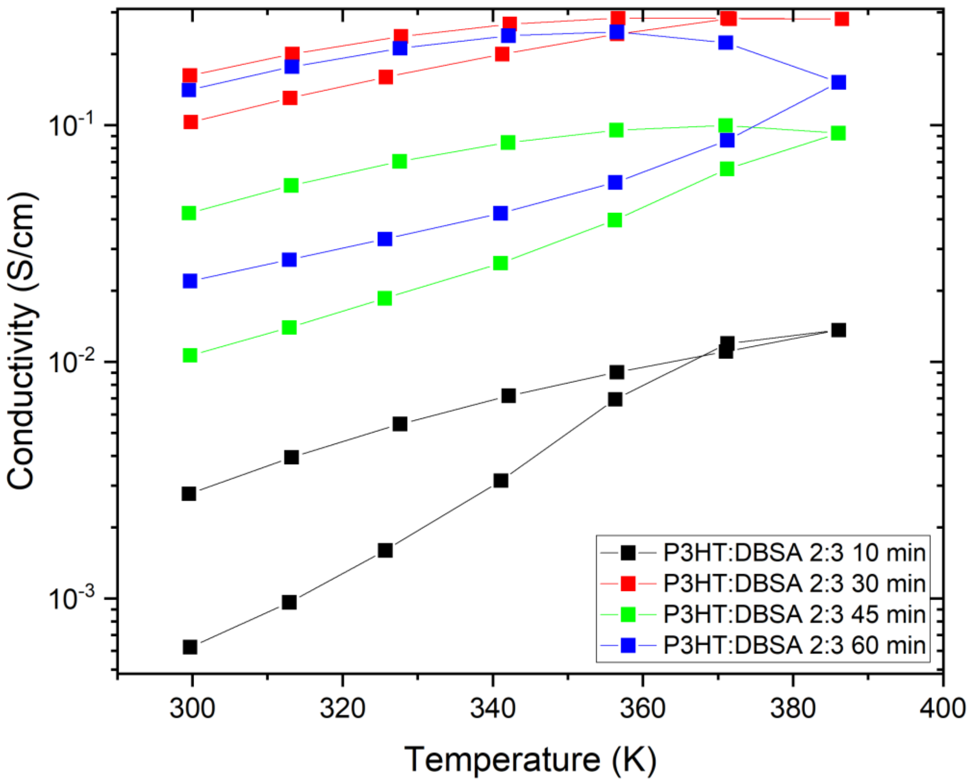

Figure S67. Relationship of pellets with different sputtering time conductivity with temperature of measurement

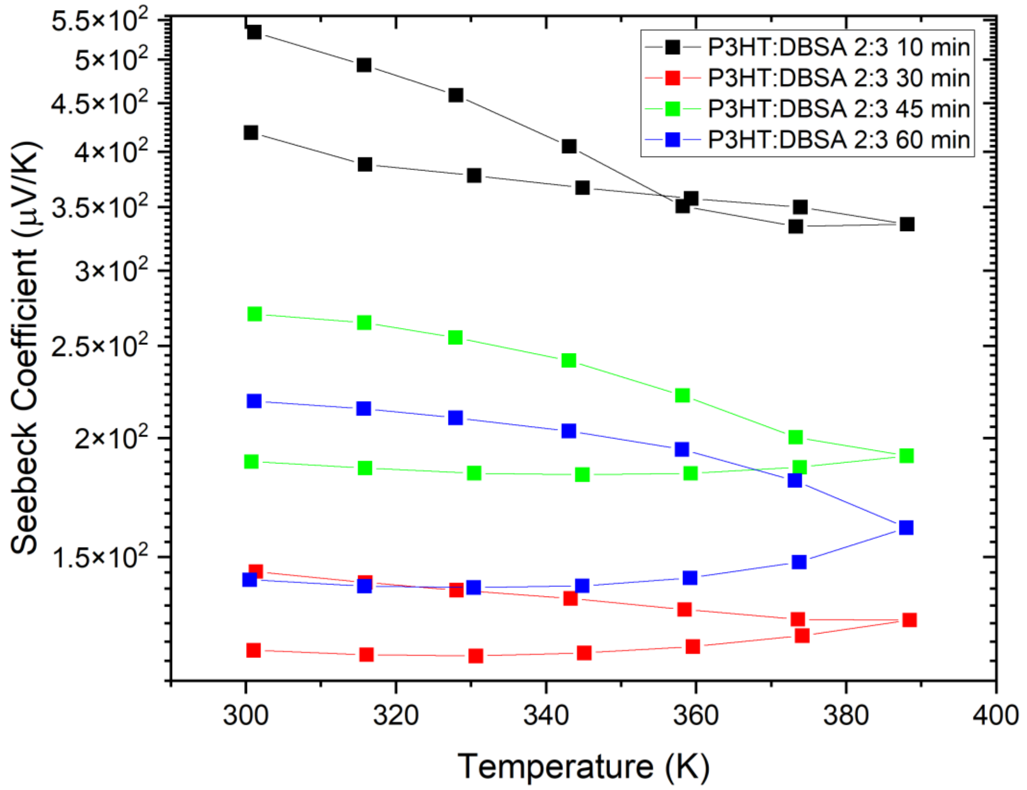

Figure S68. Relationship of pellets with different sputtering time Seebeck coefficient with temperature of measurement

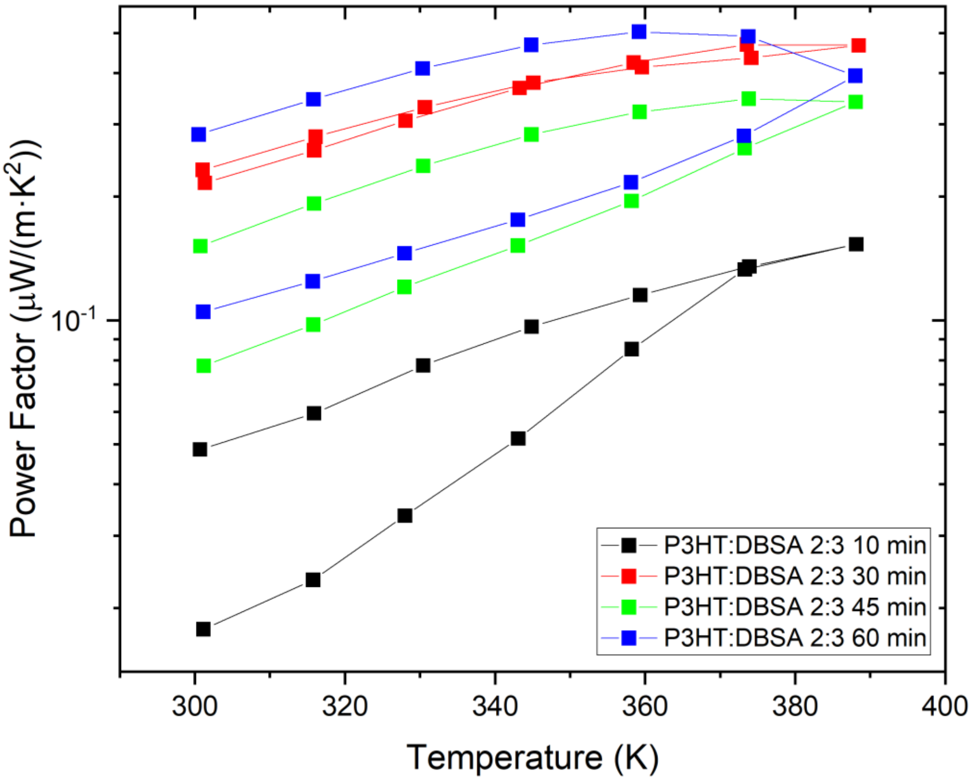

Figure S69. Relationship of pellets with different sputtering time power factor with temperature of measurement

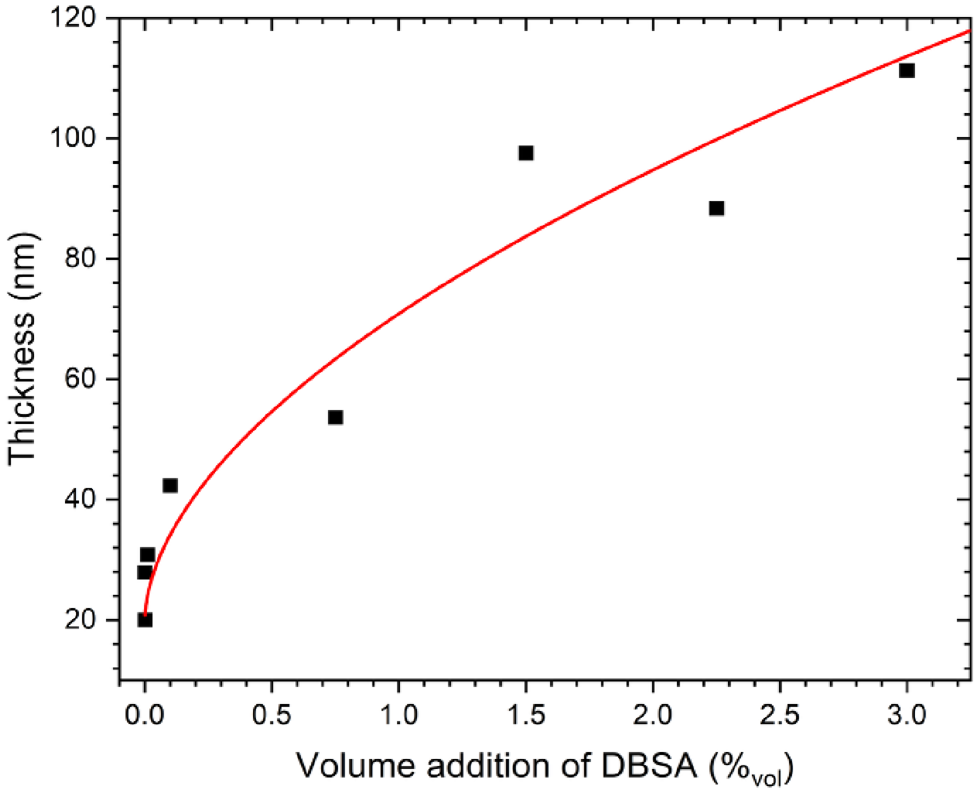

Figure S70. Relationship of thickness with increasing addition of DBSA in sample
